# Center for Interdisciplinary Research in Health (CIIS) National Meeting 2023

**DOI:** 10.1186/s12919-023-00269-8

**Published:** 2023-08-21

## Paulo J. G. Bettencourt^1,2^, Ana Mineiro^1,3^, Paulo Alves^1,4^, Nuno Rosa^1,5^, André Correia^1,5^, Marlene Barros^1,5^

### ^1^ Universidade Católica Portuguesa, Center for Interdisciplinary Research in Health, Portugal; ^2^ Universidade Católica Portuguesa, Faculty of Medicine, Lisboa, Portugal; ^3^ Universidade Católica Portuguesa, Instituto de Ciências da Saúde, Lisboa, Portugal; ^4^ Universidade Católica Portuguesa, Instituto Ciências da Saúde, Escola Enfermagem (Porto), Portugal; ^5^ Universidade Católica Portuguesa, Faculty of Dental Medicine (FMD), Viseu, Portugal

#### **Correspondence:** Paulo J. G. Bettencourt (pbettencourt@ucp.pt)

*BMC Proceedings 2023,* **17(9):**

The Center for Interdisciplinary Research in Health (CIIS) is the research center of the Universidade Católica Portuguesa (UCP) focused on health care. The Center is organized in five platforms, and distributed in four geographies across Portugal: Lisbon, Porto, Viseu and Sintra (Table 1). The center has currently 155 active researchers and attracted funds exceeding 10M€.

For the first time ever, CIIS has organized a National Event that included researchers from all platforms and disciplines, in a truly interdisciplinary and translational scientific event, counting 117 registered participants and 120 abstracts. The meeting took place at the Faculty of Medicine, in the Sintra campus, on the 31^st^ March and 1^st^ April 2023.

The Scientific Committee of the CIIS National Meeting decided that the theme for the meeting is *Interdisciplinary Health Care*. Rather than clustering researchers by platform or discipline, we decided to create three working sessions that are inclusive to everyone and not restricting the presentations by discipline, being therefore, interdisciplinary. These are: 1 – *Translational Care*; 2 – *Clinical Care*; and 3 – *Community Care*.

The meeting was held in the presence of the Universidade Católica Portuguesa Rector Professor Isabel Capeloa Gil, the Vice-Rector Professor Peter Hanenberg, the Director of the CIIS, Professor Marlene Barros, the Director of the Faculty of Medicine, Professor António Almeida and the guest speaker Professor Tomáš Zima, Charles University, Prague, Czech Republic, and hosted by the Deputy Director of the CIIS, Professor Paulo J. G. Bettencourt.

For two days, papers were presented by invited speakers within each session, and posters were presented by CIIS researchers and students, in a highly anticipated poster session. All abstracts were peer-reviewed. To bring further excitement to the poster session, the Meeting´ Scientific Committee selected the best poster from each platform to receive the Best Poster Award. Finally, the CIIS platform coordinators presented their plans and vision for the future.

Following the success of this meeting, the Scientific Committee of the National Meeting, decided to implement yearly meetings of the Center.

We would like to acknowledge all CIIS members, staff and students that accepted the challenge of participating in this event, presenting their most recent data, sharing their knowledge, and making this truly an interdisciplinary health care event.

We hope this meeting has contributed to share the latest scientific achievements of all members and promoted the beginning of new collaborations for the future, keeping in mind the main goal of improving health care with an interdisciplinary view, to ultimately improve quality of life, with humanity and spirituality at the center of all scientific quests.

**Acknowledgements**

The authors acknowledge the funding from Fundação para a Ciência e a Tecnologia (FCT), under the project UIDP/04279/2020 and UIDB/04279/2020.

**Table 1:** Platforms of the Center for Interdisciplinary Research in Health

Name	Location	Head
Neurosciences	Lisbon and Porto	Prof. Ana Mineiro
Nursing	Lisbon and Porto	Prof. Paulo Alves
CatólicaMed	Sintra	Prof. Paulo Bettencourt
SalivaTec	Viseu	Prof. Nuno Rosa
Precision Dental Medicine	Viseu	Prof. André Correia

## Oral Presentations

### Day 1 – 31^st^ March 2023

#### O1 - Molecular & Immunological approaches in oral inflammatory diseases: a bridge to precision medicine

##### Karina Mendes^1,2^, Ana T. P. C. Gomes^1,2^, Marla Pinto^2^, Tiago Marques^1,2^, Maria Correia^1,2^, Nuno Rosa^1,2^

###### ^1^ Universidade Católica Portuguesa, Center for Interdisciplinary Research in Health, Lisboa, Portugal; ^2^ Universidade Católica Portuguesa, Faculdade de Medicina Dentária, Viseu, Portugal

####### **Correspondence:** Karina Mendes (kmendes@ucp.pt)

*BMC Proceedings 2023,* **17(9):**O1

Oral infections are caused by diverse bacterial, viral and fungal pathogens, which in many cases are associated with a negative impact on patient's QoL*.* Indeed, infections trigger an immune response to respond effectively to a pathogen that can result in inflammatory diseases within the oral cavity.

In this context, periodontitis, the most common chronic inflammatory disease of human is caused by interactions between periodontopathic bacteria, host immune responses and environmental factors (e.g. smoking), representing a major burden on healthcare. Thus, it is important to gain further insight about molecular mechanisms involved in periodontitis development and progression into different stages (I, II, III and IV) and grades (a, b and c), a new classification scheme proposed in 2018.

Several signaling pathways have been implicated in periodontitis like MAPK Mitogen-activated protein kinases, nuclear factor kappa B (NF-κB), Janus kinase (JAK)-signal transducer and activator of transcription (STAT), TAM receptor tyrosine kinases (RTKs) and the Wnt pathway. However, studies performed in saliva and associating most of these signaling pathways with human periodontitis pathogenesis and severity are limited.

Because of that, its essential to identify and quantify specific biomarkers related to these signaling pathways at distinct stages of periodontitis, as a potential tool to support prognosis and clinical management of periodontitis cases, contributing towards a Predictive, Participatory, Preventive, and Personalized medicine.

In this work, a pilot study including molecular data generated from saliva samples of patients diagnosed at different stages of periodontitis will be presented, inferring about the involvement of specific signaling pathways in human periodontitis progression.

This work has been approved by the Comissão de Ética para a Saúde of Universidade Católica Portuguesa [project CES157 – Estratégias Moleculares e Imunológicas em doenças inflamatórias: a ponte para a Medicina de Precisão (OralPreciseMed)].

**Funding**

This work is financially supported by National Funds through FCT – Fundação para a Ciência e a Tecnologia, I.P., under the project UIDP/04279/2020. Thanks, are also due to FCT and UCP for the CEEC institutional financing Ana T.P.C. Gomes (CEECINST/00137/2018/CP1520/CT0022) and Karina Mendes (CEECINST/00070/2021-CIIS-Júnior).

#### O2 - Validation of salivary biomarkers for Inflammatory Bowel Disease diagnosis and monitoring

##### Ana T. P. C. Gomes^1,2^, Pedro Pereira^2^, Rúben Martins^2^, Karina Mendes^1,2^, Maria Correia^1,2^, Nélio Veiga^1,2^, Pedro Lopes^1,2^, Claúdio Rodrigues^3^, Paula Ministro^3^, Nuno Rosa^1,2^

###### ^1^ Universidade Católica Portuguesa, Center for Interdisciplinary Research in Health, Lisboa, Portugal; ^2^ Universidade Católica Portuguesa, Faculdade de Medicina Dentária, Viseu, Portugal; ^3^ Department of Gastroenterology, Tondela-Viseu Hospital Centre, Viseu, Portugal

####### **Correspondence:** Ana T. P. C. Gomes (apgomes@ucp.pt)

*BMC Proceedings 2023,* **17(9):**O2

Inflammatory bowel disease (IBD) is a chronic inflammatory disorder of the gastrointestinal tract with a rising incidence worldwide, imposing a considerable burden on health services. IBD etiopathogenesis of is partly understood and includes both genetic and environmental factors which induce an abnormal immune response. The disease can be present 2 forms: Crohn’s disease (localized in the terminal ileum affecting all layers of the intestine) and ulcerative colitis (localized in the rectum and the colon and limited to the mucosa). The guidelines for IBD diagnosis in adults require a comprehensive physical examination and a review of the patient’s history. Various tests, including blood tests, stool examination, endoscopy, colonoscopy, biopsies, and imaging studies help exclude other causes and confirm the diagnosis.

Colon biopsy and blood samples represent a powerful source of novel biomarkers supporting differential diagnosis. In addition to their potential in diagnosis, novel biomarkers such as miRNAs, inflammatory biomarkers, fecal and mucosal microbiota may play a critical role in predicting therapeutic efficacy as well as disease recurrence and severity.

The identification, quantification and/or validation of biomarkers is primarily performed in tissue, blood and fecal samples but only few studies have been done with saliva, which reflects the same type of biomarkers, allowing a noninvasive sample collection.

In this work, an extensive oral characterization, and the quantification of salivary biomarkers such as calprotectin and total bacterial load from IBD patients recently diagnosed and/or undergoing biological therapy were evaluated. Our preliminary results showed an increased periodontitis predisposition, and an increased salivary calprotectin and total bacterial load in those patients. These results open new perspectives to improve the understanding of the potential of saliva as a powerful tool to evaluate IBD progression/therapeutic success and generates molecular data supporting the use of saliva in diagnosis, prognosis and disease/treatment monitoring towards a Predictive, Preventive, and Personalized medicine.

This work has been approved by the Comissão de Ética para a Saúde of Universidade Católica Portuguesa (project CES133 – Microbioma Oral Humano).

**Funding**

This work is financially supported by National Funds through FCT – Fundação para a Ciência e a Tecnologia, I.P., under the projects UIDP/04279/2020. Thanks are also due to FCT and UCP for the CEEC institutional financing of Ana Gomes (CEECINST/00137/2018/CP1520/CT0022), Karina Mendes (CEECINST/00070/2021-CIIS-Júnior).

#### O3 - Targeting lysosomal proteases for a host-directed therapy for tuberculosis

##### David Pires^1,2^

###### ^1^Universidade Católica Portuguesa, Católica Medical School, Center for Interdisciplinary Research in Health, Rio de Mouro, Sintra, Portugal; ^2^ Instituto de Investigação do Medicamento - iMed.ULisboa, Faculdade de Farmácia da Universidade de Lisboa, Lisbon, Portugal

####### **Correspondence:** David Pires (dpires@ucp.pt)

*BMC Proceedings 2023,* **17(9):**O3

Tuberculosis is a disease caused by the bacteria *Mycobacterium tuberculosis* (Mtb) which latently infects one-quarter of the human population and is the leading cause of death by an infectious agent. Contributing to the challenge of this old disease is the lack of (1) an effective vaccine, (2) reliable biomarkers for latent infection, (3) the limited number of effective antimicrobial drugs, (4) a prolonged therapeutical regime, and (5) the evolution of multi-drug resistant strains. This increasingly foments the need for novel therapies that target the bacterial niche or improve the host response, alone and in combination with the current conventional therapy. We have been probing the intracellular niche of Mtb, the macrophage, for how these bacteria survive and replicate inside them while impairing their bactericidal response. In that search, we found that Mtb induces a decrease in the expression of a group of proteolytic enzymes, the cathepsins, that participate in key cellular processes regulating homeostasis, cell death, inflammation, antigen presentation, and microbial killing. Regarding Mtb infection, this downregulation results in improved bacterial survival and replication inside macrophages as well as poor lymphocyte priming by the infected cells. To address this problem, we have been exploring the different pathways by which cathepsin activity is regulated in our cells. So far, we found three levels of cathepsins regulation that can be manipulated to our advantage: One is by targeting miRNAs to restore cathepsins gene expression; another by targeting cystatins, the natural inhibitors of cathepsins, to restore their activity; and finally, by using saquinavir, a repurposed inhibitor of the HIV protease that unexpectedly improves the activity of some human cathepsins. Together, these strategies were shown to improve the intracellular killing of Mtb by macrophages, as well as enhance the ability of these cells to prime CD4^+^ T-lymphocytes and induce their proliferation and IFNγ secretion. Our approach suggests a potential host-targeted strategy that can be developed as a complementary therapy to current antibiotics.

Human monocytes were isolated from buffy-coats of healthy human donors provided by the National Blood Institute (Instituto Português do Sangue e da Transplantação, IP, Lisbon, Portugal).

This study was supported by the grants from National Foundation for Science, FCT (Fundação para a Ciência e Tecnologia), Portugal, PTDC/SAU-INF/28182/2017 and EXPL/SAU-INF/0742/2021.

#### O4 - Exploring the biological properties and regenerative potential of biomaterials using cell culture models

##### Ana Sofia Duarte^1,2^, Bruna L. Correia^1,2^, Maria Bartolomeu^1,2^, Karina Mendes^1,2^, Ana T.P.C. Gomes^1,2^

###### ^1^ Universidade Católica Portuguesa, Center for Interdisciplinary Research in Health, Viseu, Portugal; ^2^ Universidade Católica Portuguesa, Faculdade de Medicina Dentária, Viseu, Portugal

####### **Correspondence:** Ana Sofia Duarte (asduarte@ucp.pt)

*BMC Proceedings 2023,* **17(9):**O4

Life expectancy has improved significantly and, along with the declining birthrate, has contributed to the aging of populations, especially in industrialized countries. Alas, aging is intrinsically associated with the incidence of health problems including bone and tooth loss that require suitable solutions to support the quality of life. To meet these demands, significant research efforts have been undertaken to develop novel biomaterials, both orthopedic and dental implants.

The field of biomaterials for bone tissue engineering is increasingly evolving. The most recent generations of biomaterials have increasingly more activity and interaction with the biological environment and stimulate the regeneration of functional tissue.

Natural polymers and compounds have been combined with each other to improve workability and are strategically integrated with ceramics or bioactive glasses to reinforce the structure of the final system, thus producing composites with a better mechanical performance.

Our research group has been focused on the biological characterization of different added-value materials and composites, namely by evaluating their antimicrobial, biocompatibility, and regenerative properties.

Some of our recent work results allowed us to conclude that marine fungal extracts, as well as sol–gel-derived bioactive glass nanoparticles, have inhibitory effects on the growth of *C. albicans* and *E. faecalis* (main pathogens in persistent root canal infections). Additionally, we have characterized cuttlefish bone powders for endodontic applications.

We are also committed to developing strategies for monitoring cell response to these biomaterials at the molecular level that could be used to follow inflammation and osteoconduction.

**Funding**

This work is financially supported by National Funds through FCT – Fundação para a Ciência e a Tecnologia, I.P., under the project UIDB/04279/2020 and by Programa Operacional Capital Humano e Fundo Social Europeu (FSE), under the project Indig (POCH-02-53I2-FSE-000025). Maria Bartolomeu and Bruna L. Correia thank the UCP for the Junior Researcher position and the Research grant, respectively, under the project Indig. Thanks are also due to FCT and UCP for the CEEC institutional financing of Ana T.P.C. Gomes (CEECINST/00137/2018/CP1520/CT0022), Karina Mendes (CEECINST/00070/2021-CIIS-Júnior) and Ana Sofia Duarte (CEECINST/00137/2018/CP1520/CT0013).

### Day 2 – 1^st^ April 2023

#### O5 - Psychological autonomy and central pattern generator activation in a spinal cord injury patient during immersive motor imagery BMI-Driven VR System

##### Carla Pais-Vieira^1^, Miguel Pais-Vieira ^1,2^

###### ^1^ CIIS - Centro de Investigação Interdisciplinar em Saúde Porto, Universidade Católica Portuguesa, Porto, Portugal; ^2^ iBiMED - Instituto de Biomedicina, Departamento de Ciências Médicas, Universidade de Aveiro, Aveiro, Portugal

####### **Correspondence:** Carla Pais-Vieira

*BMC Proceedings 2023,* **17(9):**O5

**Background**

Previous studies demonstrate that motor imagery -based Brain Machine Interface (BMI) and Virtual Reality (VR) rehabilitative training improve neural plasticity in Spinal Cord Injury (SCI) patients even after several years of injury. We have recently described that mentally imagine a walking action within a multimodal highly realistic BMI enhanced the illusory sensation of having a control one's own actions in an SCI patient and generated a self-reported unique pleasurable experience and lower limb movements, even though the patient was not aware of them.

**Materials and methods**

Here, we have analyzed the subjective patient experience with Electroencephalogram (EEG)-based BMI-VR system from qualitative data collected through unstructured interview. Additionally, we have analyzed patterns of lower limb movements in a previously video recorded session to describe their main characteristics.

**Results**

A SCI patient participant gave written informed consent for a protocol approved by the Hospital Senhora da Oliveira Ethics Committee; n° 15/2020. Firstly, a conventional content analysis was used for data analysis which results led to the extraction of four categories for sensations: self-determined acts, those that reflect one's will; acts that are fully endorsed by the self, volition, and retrieval of pre-injury memories. Those categories address the basic psychological need of autonomy reinforced by a sense of integrity personalized into the patient's autobiographical past. Moreover, lower limb movements in one limb were dorsiflexion occurred were compared with surface muscle movements in the opposite limb. Analysis of muscle patterns revealed a pattern of muscle movements that alternated between the two limbs, occurring at ~0.07Hz (i.e., once every 14 seconds). When the patient was placed in the same position, but VR was not used, no movements were generated.

**Conclusions**

Taking together, these results suggest that the use of a BMI combined with highly realistic virtual reality as well as tactile and thermal feedback can produce an experience of psychological autonomy, which is critical and predictive of quality of life, and recruit a central pattern generator that originates gait-like muscle movement patterns.

#### O6 - Nutrition in Cancer: into a growing future

##### Paula Ravasco^1^

###### ^1^ Universidade Católica Portuguesa, Catolica Medical School and Center for Interdisciplinary Research in Health, Lisbon, Portugal

####### **Correspondence:** Paula Ravasco (pravasco@ucp.pt)

*BMC Proceedings 2023,* **17(9):**O6

Muscle wasting and cachexia in cancer, derive from a negative balance of protein and energy caused by various combinations of reduced food intake and metabolic abnormalities. The main features are a strong tendency toward catabolism and a negative protein–energy balance that is difficult to restore. The reversal or prevention of cancer cachexia and muscle wasting represent a major clinical challenge, thus urging an adequate, early and integrated nutritional intervention throughout the disease course and treatments.

The main objective of nutritional intervention is to optimize your energy intake, with the purpose of improving your well-being, quality of life and better tolerate of antineoplastic treatments. To achieve this goal, it is mandatory to carry out the nutritional assessment to detect the needs of the patient and later a personalized food plan, aiming at: the objective of antineoplastic treatment, expected toxicities, energy needs, symptoms in need of modulation, swallowing capacity, psychosocial factors and above all the wishes and preferences of the patient.

Early nutritional support has the potential to reduce the risk for therapy-threatening adverse events and optimise the likelihood of treatment success and long-term survival. Although the optimal nutrient content for “an anti-cachexia diet” is still not defined, ESMO and ESPEN guidelines stress the need for maintaining calorie and protein intake. Clinical studies do show potential benefits for some specific nutrients, especially when combined with exercise training.

*Protein*: a range of protein intake between 1.5-2.0 g/kg/day seems needed to promote muscle mass balance, with beneficial effects in patients’ body composition.

*Fish oil and eicosapentaenoic acid (EPA)*: could help to improve appetite, food intake, body weight, and muscle mass in individuals at risk for body composition alterations. High protein oral nutrition supplements enriched with EPA may help ameliorate weight and muscle mass loss to a greater extent than isocaloric control supplements.

*Vitamins and minerals* during the disease trajectory, there is risk of micronutrient deficiency. To date there is insufficient evidence to support the use of vitamin or mineral supplements. Studies showed that side effects of therapy such as vomiting or diarrhea might deplete vitamins A and E. Zync supplementation has been studied in the context of dysgeusia with possible positive impact in improving intake. Sufficient vitamin D might be needed for other supplements to be effective. Ensuring adequate levels may be advantageous in the prevention or treatment of low muscle mass.

#### O7 - Clinical Wound Support I Decision-making in wound monitoring and treatment

##### Paulo Alves^1,2^, Raquel Silva^1,2^; Paulo Ramos^1,3^, Irene Oliveira^1,2^, Luís Sá^1,2^, João Amado^1^, João Neves-Amado^1,2^, Paula Teixeira^4^, Maria Vasconcelos^5^, Pedro Salgado^6^

###### ^1^ Universidade Católica Portuguesa, Center for Interdisciplinary Research in Health, Portugal; ^2^ Universidade Católica Portuguesa, Instituto Ciências da Saúde, Escola Enfermagem (Porto), Portugal; ^3^ USF Corino Andrade, Administração Regional de Saúde do Norte IP, Portugal; ^4^ Unidade Local de Saúde de Matosinhos, Hospital Pedro Hispano, Portugal; ^5^ Fraunhofer AICOS Portugal, Porto, Portugal; ^6^ Health Solutions Manager, F3M Information Systems, SA, Braga, Portugal

####### **Correspondence:** Paulo Alves (pjalves@ucp.pt)

*BMC Proceedings 2023,* **17(9):**O7

**Background**

The World Health Organization considers wounds and all their problems as the new hidden epidemic. Ageing associated with chronic diseases and other morbidities are a common factor in this phenomenon, considered a national and international public health problem and a concern for the safety of patients, with clinical and socio-economic impact. Health professional assistance tools to manage information and support their decision are seen as an important resource because they reduce time, reduce costs, provide security to the professional and improve their decisions.

**Objective**

Build a digital and portable system to support monitoring and clinical decision-making in prevention and treatment of chronic wounds.

**Methods**

Qualitative and quantitative approach with a series of different methodologies: first a prospective observational multicentric study to develop a tool for capturing the image of the wounds/dressings applied, with the ability to focus and detect the wound/dressings, as well as to identify the perilesional skin, edges and measures of the wound, types of tissue on the wound bed and the areas of exudate transfer (dressings); Develop algorithms for semi-automatic determination of wound properties based on acquired images; and build decision trees for the monitoring and treatment of chronic wounds; second with a qualitative approach was a based on individual interviews, Focus group, usability tests, ideation and co-creation sessions with experts with the goal of identifying system actors, guidelines, best practices in wound monitoring, and algorithm development to diagnosis and treatment; the third the validation of clinical algorithms through a prospective observational multicentric cohort study. At the end a system of clinical decision-making support can be created in the diagnosis and treatment of chronic wounds.

**Results**

Validation of the different components built for clinical algorithms, alerts, recommendations for the treatment and a wound imaging tool with ability to focus, detection, acquisition, and automatic image correction, speeding up the semi-automatic recording of wound characteristics (size, types of tissue, perilesional skin).

**Conclusion**

With the use of this technology will be made possible to achieve better care to people with chronic wounds, predictably faster healings and, consequently, greater satisfaction and quality of life of people.

**Funding**

This work is financially supported by project ClinicalWoundSupport: Wound Analysis to Support Clinical Decision (POCI-01-0247-FEDER-048922; LISBOA-01-0247-FEDER-048922)

**Keywords**

Chronic wounds; decision making; prognosis; treatment; wounds and injuries.

#### O8 - HOPE2BRAIN+: Evaluating the effectiveness of a hope-promoting multilevel intervention program for increasing executive functions and quality of life in children with cancer disease

##### Joana Rato^1,2^, Zaida Charepe^1,2^

###### ^1^ Institute of Health Sciences, Universidade Católica Portuguesa, Lisboa, Portugal; ^2^ Center for Interdisciplinary Health Research (CIIS), Universidade Católica Portuguesa, Lisboa, Portugal

####### **Correspondence:** Zaida Charepe (zaidacharepe@ucp.pt)

*BMC Proceedings 2023,* **17(9):**O8

**Background**

Hope2Brain+ is a research project of Hope2Care integrated into the Nursing Research Platform in association with the Translational Neuroscience Platform - Brain and Behavior Research Lab. Studies conducted in Portugal have highlighted the fragility of the health condition and general well-being of children with cancer disease and their parents^1-3^. The project evaluates an approach supported by Hope Therapy (HT). It has as its objectives: i) test the effect of a hope therapy program for parents operationalized in individual and group intervention on the levels of hope, comfort, quality of life, and the burden of parents of the child with oncologic disease; i) test the effect of the HT program on children operationalized in group intervention (storytelling - Storytime) on the dimensions of executive functioning and anxiety; iii) test the effect of the combination of the two programs of HT.

**Materials and methods**

Descriptive and comparative study targeting children aged 6 to 10 and their parents. The different phases of the project include stage I (pre-experimental study), stage II (experimental study), and stage III (qualitative study). In stage II, we plan to implement and test the effect of HT operationalized in individual and group interventions. The group intervention carries eight weekly (2-month) sessions, each lasting 02h00 (duration of the intervention foreseen for a group of 12 participants). To the experimental group (G1), hope therapy applies to the parents. The experimental group (G2) will receive the children's HT (also every week) using storytelling with stories selected for this purpose. For the experimental group (G3), the HT will simultaneously apply to parents and children. For the control group (G4), a group intervention based on the "free" sharing of experiences among the participants (therapy in use) in different periods. The review of this study by the Ethics Commission is ongoing.

**Results**

By evaluating the effectiveness of a multilevel program based on HT, we hope to improve well-being in the dimensions of comfort, quality of life, and prevention of emotional overburdening of parents. The storytelling intervention with children is also expected to empower them to solve problems. Children receive reassurance from their parents and are accompanied by guided reflections on the obstacles of the characters in the stories worked on, will lead to an increase in resilience during their hospital stay.

**Conclusions**

The research will provide an opportunity to improve emotional support care and the general well-being of this population.

**References**

1. Charepe, Z. O impacto dos grupos de ajuda mútua no desenvolvimento da esperança dos pais de crianças com doença crónica: Construção de um modelo de intervenção colaborativa. 2011; 578 pp. Lisboa: UCP. Tese apresentada para obtenção do grau de doutor em enfermagem à Universidade Católica Portuguesa.

2. Marques, R.; Dixe, M.; Querido, A. O Impacto do Cuidar da Pessoa com Doença Crónica e Avançada na Qualidade de Vida dos Cuidadores. Referência. III Série. Suplemento. Actas e Comunicações da XI Conferência Ibero Americana de Educação em Enfermagem, 2011; 1:323.

3. Querido, A. A Promoção da Esperança em Fim de Vida - Avaliação da Efetividade de um Programa de Intervenção em Pessoas com Doença Crónica Avançada e Progressiva, 2013; 312pp. Lisboa: UCP. Tese apresentada para obtenção do grau de doutor em enfermagem à Universidade Católica Portuguesa.

#### O9 - Cognition in psychiatric disorders

##### Frederico Simões do Couto^1^

###### ^1^ Católica Medical School, Universidade Católica Portuguesa, Lisboa, Portugal

*BMC Proceedings 2023,* **17(9):**O9

Excluding dementia, cognition is usually not considered a core feature of psychiatric disorders. However, it is one of the most important factors associated with short- and long-term impairment.

Acute cognitive changes, a concept similar to “hot cognition”, are related to emotional and mood symptoms, e.g., depressed patients tend to have cognitive distortions towards pessimism [1] or OCD patients have a different pattern of risk assessment when compared to nondepressed and non-OCD persons, respectively.

Persistent cognitive deficits (“cold cognition”) are extremely important because they can be preventable, either through the treatment of psychiatric disease or through specific interventions on the underlying biological mechanisms. Depression is a risk factor for dementia and is amenable to prevention. However, only some types of depression have been associated with dementia, probably due to its biological and clinical heterogeneity [2]. Understanding the mechanism of this cognitive impairment has been one of our targets. We are now trying to understand the roles of the endocannabinoid system and NMDA receptors in the persistent cognitive impairment of depressive disorders. Cognitive impairment in another psychiatric disorder, schizophrenia, has also been associated with NMDA receptors. The modulation of these receptors can attenuate some cognitive deficits, especially those related to the frontal lobe, in animals [3]. Clinical trials targeting this receptor have already started.

In alcohol use disorders, cognitive deficits are well known, and they can hamper alcohol dependence treatment. The pattern of cognitive impairment can guide the choice of treatment of the disorder, as some patients remain longer without drinking if a certain pattern is dealt specifically with cognitive rehabilitation.

Although cognitive symptoms are not the core symptoms of most psychiatric disorders, we are very interested in how they can be prevented.

**References**

1. Mendes T, et al. Memory awareness in patients with Major Depressive Disorder. J Psychiatr Res. 2021; 137: 411-418.

2. Simões do Couto F, et al. Depression with melancholic features is associated with higher long-term risk for dementia. J Affect Disord. 2016; 202: 220-9.

3. Tanqueiro SR, et al. Sustained NMDA receptor hypofunction impairs brain-derived neurotropic factor signalling in the PFC, but not in the hippocampus, and disturbs PFC-dependent cognition in mice. J Psychopharmacol. 2021; 35(6): 730-743.

#### O10 - MAIEC Project- Research on Community empowerment at the service of the sustainable development goals

##### Pedro Melo^1^

###### ^1^ Universidade Católica Portuguesa, Center for Interdisciplinary Research in Health, 4169-005 Porto, Portugal

####### **Correspondence:** Pedro Melo (pmelo@ucp.pt)

*BMC Proceedings 2023,* **17(9):**O10

**Background**

The Community Assessment, Intervention and Empowerment Model (MAIEC) was developed in 2016 in the context of the PhD in Nursing at the Catholic University of Portugal. In 2017, the project MAIEC: Community Empowerment and Nursing decision-making" was created, based at the Centre for Interdisciplinary Research in Health of the Universidade Católica Portuguesa. The aim of this project is to evaluate the impact of using MAIEC in improving community management and empowerment from different communities in different issues.

**Methodology**

The project has four work packages (WP), where the MAIEC research protocol is developed. The protocol begins with assessing the level of community empowerment using the Portuguese version of the Empowerment Assessment Rating Scale (with Focus Group as methodological strategy). Then, the diagnostic activity proposed by the MAIEC clinical decision matrix is applied and the interventions prescribed by the same matrix are developed and the results are assessed. With an interval of at least 1 year, the level of community empowerment is reassessed. WP1 relates to school communities, WP2 relates to hospital communities, WP3 relates to Epidemiological Surveillance of Nursing Diagnoses and WP4 relates to environmental processes. Each WP has multiple projects.

**Results**

MAIEC project made it possible to identify the level of community empowerment and the diagnosis of community management in various communities and different issues. It also made it possible to identify the improvement in the level of community empowerment regarding MAIEC application. In the set of 4 WP, the project responds to 10 of the 17 Goals for Sustainable Development of the UN.

**Conclusion**

MAIEC project is a multidisciplinary, innovative project that responds to the production of evidence, particularly in Community Health and Public Health Nursing and contributes to respond to more than half of the Goals for Sustainable Development proposed by the UN.

This article includes information that result of the project HAC4CG- Heritage, Art, Creation for Climate change. Living the city: catalyzing spaces for learning, creation and action towards climate change (NORTE-01-0145-FEDER-000067), supported by Norte Portugal Regional Operational Programme (NORTE 2020), under the PORTUGAL 2020 Partnership Agreement, through the European Regional Development Fund (ERDF), and submitted for the approval of the ethics committee of Technology, Social Sciences and Humanities of the Catholic University of Portugal.

#### O11 - Epigenetics in hematological malignancies

##### António de Almeida^1,2^

###### ^1^ Católica Medical School, Universidade Católica Portuguesa, Lisbon, Portugal; ^2^ Department of Hematology, Hospital da Luz, Lisbon, Portugal

*BMC Proceedings 2023,* **17(9):**O11

The concepts of classical genetics predict that the phenotype of the organism is dictated by the genetic sequence of its DNA, the genotype. However, this does not explain the phenotypic differences observed in organisms and cells with identical genetic codes.

These differences are partially explained by epigenetics. Epigenetics is defined as the modulation of gene expression through the addition or removal of small molecules to DNA or the proteins attached to it, such as histones and transcription factors.

The best known epigenetic alteration is DNA methylation. This modification occurs mainly in the promoter regions of the genes, silencing their expression. Associated with DNA methylation, there are changes in proteins closely linked to DNA, especially histones. Through acetylation, methylation and phosphorylation of these nuclear proteins, gene expression can be increased or decreased. Thus, in normal tissues, epigenetic changes allow constant differential regulation of gene expression.

There are more and more data that indicate the relevance of epigenetics in oncogenesis. Normal epigenetic patterns are altered in neoplastic cells, affecting the expression of tumor suppressor genes, apoptosis, angiogenesis and DNA repair.

In vitro studies have shown that the treatment of neoplastic cells with epigenetic agents, namely histone deacetylase inhibitors and demethylating agents, can lead to increased apoptosis or normalization of the differentiation of the same cells. These data point to the antineoplastic potential of epigenetic therapy.

In the context of clinical trials, Vorinostat, a histone deacetylase inhibitor, produced responses in 30% of patients with cutaneous T lymphoma, thus becoming a therapeutic option in these patients.

In the treatment of myelodysplastic syndromes, epigenetic therapies reduced the need for transfusions and improved the quality of life of patients, also doubling the life expectancy of patients with high-risk myelodysplastic syndromes when compared to conventional therapies.

The knowledge of epigenetics and the identification of its alterations in hematological neoplasias opened new doors in these pathologies. The introduction of new agents and new combinations may produce better responses with less toxicity.

#### O12 - From biostatistics to AI in biomedical sciences: a vision for the future of research

##### J. Pereira^1^

###### ^1^ Universidade Católica Portuguesa, Faculdade de Medicina, Centro de Investigação Interdisciplinar em Saúde, Portugal

*BMC Proceedings 2023,* **17(9):**O12

Nothing replaces benchwork in a laboratory. But what about everything else, from the initial literature review to generate research questions and hypotheses, all the way to the analyses of the results, their discussion, and final conclusions?

For too long, machine learning algorithms have been seen as a perhaps fanciful extension to biostatistics, the basic grammar of research. Interesting to an extent, certainly useful for very large datasets, but far removed from the more practical research work in your typical biomedical laboratory. In addition, Artificial Intelligence (AI), with its ambitious promises but limited deliverables, is also usually dismissed. Recent advances, however, notably the development of the Generative Pre-trained Transformer (GPT), will affect scientific research and medical practice in profound ways.

A lot has been already written and discussed about ChatGPT, as well as about similar applications. Many more will appear though. Moreover, most comments fail to grasp the non-linear acceleration of the development of such technologies and only focus on the possibilities and limitations of what exists “now”. With years compressed into weeks, such a perspective is not only inadequate, it is profoundly limiting of what could and should be done in preparation for the future. We are already trying to catch up, but catching up will become ever more complicated.

There is a continuum, from biostatistics to machine learning, touching on blockchain and quantum/graphic processing, that leads to existing and future AI algorithms that will change our lives beyond recognition. Not in 10 or 20 years, but in 1 or 2 years. Such a building of knowledge must be acknowledged by researchers from all backgrounds in order to mitigate risks and capitalize on gains – there are plenty of both. The train has already departed the station. Are we on board?

#### O13 - Multidimensional monitorization of nutritional interventions during cancer treatment – Patient centered approach including noninvasive saliva monitoring

##### Raquel M Silva^1,2^

###### ^1^ Universidade Católica Portuguesa, Faculdade de Medicina Dentária, Viseu, Portugal; ^2^ Universidade Católica Portuguesa, Center for Interdisciplinary Research in Health, Viseu, Portugal

####### **Correspondence:** Raquel M Silva (rmsilva@ucp.pt)

*BMC Proceedings 2023,* **17(9):**O13

Despite the optimization of diagnosis and treatments, cancer continues to take a huge toll on society and remains a challenge in healthcare. It is recognized that further improvements in understanding, preventing, diagnosing, and treating cancer, but also in patients’ quality of life, are needed. Our work is dedicated to the identification of cancer-induced alterations that can be used for diagnosis, prognosis, or development of therapeutic targets, with a special focus on saliva as a source of biomarkers.

Malnutrition is common in cancer patients, as a consequence of the cancer itself but also of the anticancer treatments. This condition has a negative impact on the quality of life and is a predictor of poor clinical outcomes such as increased chemotherapy toxicity and decreased survival. Nutritional interventions have been designed and evaluated to identify and treat malnutrition, and to prevent metabolic and nutritional alterations that influence recovery and survival. However, the impact of these nutritional interventions is not well studied at the molecular level.

We propose a patient centered multidimensional monitorization of nutritional interventions during cancer treatment. Our strategy applies and extends the liquid biopsy concept to oral fluids such as saliva, to assess nutritional, inflammatory, (epi)genetic and microbial biomarkers. The development of objective, non-invasive methodologies based on molecular markers, will strengthen the assessment of the interventions and result in protocols and guidelines for improved treatment efficacy and monitoring, and better quality of life of cancer patients.

The project has been approved by the Health Ethics Committee from Universidade Católica Portuguesa (CES-UCP, nr. 243).

**Funding**

This work is financially supported by National Funds through FCT – Fundação para a Ciência e a Tecnologia, I.P., under the projects UIDB/04279/2020 and UIDP/04279/2020.

Thanks are also due to FCT and UCP for the CEEC institutional funding of Raquel M Silva (CEECINST/00137/2018/CP1520/CT0012).

#### O14 - Clinical research in dentistry - new approaches

##### Patrícia Fonseca^1^, André Correia^1^

###### ^1^ Universidade Católica Portuguesa, Faculty of Dental Medicine (FMD), Center for Interdisciplinary Research in Health (CIIS), Viseu, Portugal

####### **Correspondence:** Patrícia Fonseca (pafonseca@ucp.pt)

*BMC Proceedings 2023,* **17(9):**O14

**Background**

Clinical research in dentistry is essential for application and development of new techniques and technologies with the aim of improving patient outcomes, deliver care more efficiently and support research and education.

The aim of this work is to show the new approaches to the patient in the University Dental Clinic of the Faculty of Dental Medicine (FMD) of the *Universidade Católica Portuguesa* (UCP), with the used of newly introduced technologies, as a fundamental link in the dynamics of oral health care.

**Materials and methods**

An inventory of all the technologies used in the University Dental Clinic was used to proceed with a selection of the ones acquired in the last 5 years and establish a classification based on its supporting technology. Also, the integration of this technologies was analysed.

**Results**

A total of twelve technologies were achieved as being the ones most currently used in the University Dental Clinic of FMD-UCP. They were divided in the following categories: a) Devices/Hardware – *Cone*-*beam computed tomography* (*Planmeca ProMax*® *3D Mid*), Intraoral Scanners (3Shape® Trios 4, iTero^TM^ Element 5D plus Mobile), 3D Printer (Phrozen Sonic Mini 8k), Ostell® Beacon, and Magnification systems; b) Software: practice management system (Newsoft DS Imaginasoft); implant planning software (coDiagnostiX® Dental Wings), imagiology software (Romexis® Planmeca); c) WEB-based tools: risk analysis in implant dentistry (SAC Assessment Tool® ITI) and open access periodontology tools (University of Bern, Switzerland) - Implant diseases risk assessment (IDRA), Periodontal Chart, Periodontal Risk Assessment (PRA).

All these technologies show a basic integration level (hardware-hardware; hardware-software; software-software).

**Conclusions**

The clinical set of the Precision Dental Medicine Platform of the Center for Interdisciplinary Research in Health (CIIS) of the *Universidade Católica Portuguesa* has focused researchers in an increasingly personalized clinical care both in undergraduate and postgraduate education.

Several technologies are being used in clinical dentistry and there is still room for improvement to a task-oriented integration level in a natural/clinical workflow.

The application, validation, and creation of tools for precision care is the goal of the teaching/research dynamic as students/teachers/researchers at FMD.

It is evident that university clinical research is an important source of know-how and a way of applying and developing new technologies for a precision dentistry as well as a way of stimulating the acquisition of competencies by the students.

#### O15 - Community-based research: the pathway to improve health knowledge

##### Nélio Veiga^1,2^

###### ^1^ Faculty of Dental Medicine, Universidade Católica Portuguesa, Viseu, Portugal; ^2^ Center for Interdisciplinary Research in Health, Universidade Católica Portuguesa, Viseu, Portugal

*BMC Proceedings 2023,* **17(9):**O15

The conference will aim in giving an overview of the various research outcomes in public health and community oral health developed at the Precision Dental Medicine Platform of the Center for Interdisciplinary Research in Health (CIIS) of the UCP.

It is increasingly important to understand the distribution and determinants of diseases to be able to establish the best strategies of health promotion in different communities. Research in the community has been fundamental for guiding health programs, associated with the increase of interdisciplinarity that must exist in scientific research, and which is reflected in CIIS.

Some of the projects under development in the community will be described, focusing on research in the fields of literacy in general health and oral health and quality of life, as well as some of the main results obtained in recent years regarding the characterization of oral health in the Central region of Portugal.

This conference will also address the application of Service-Learning strategies that have allowed the identification of community needs and the active participation of students in community interventions and scientific research in oral health.

#### O16 - The Bayesian Brain Hypothesis and pain perception in migraine

##### Rita Canaipa^1^, Raquel Gil Gouveia^2^

###### ^1^ CIIS, Centre for Interdisciplinary Health Research, Institute of Health Sciences, Universidade Católica Portuguesa, Lisbon, Portugal; ^2^ CIIS, Centre for Interdisciplinary Health Research, Faculty of Medicine, Universidade Católica Portuguesa, Lisbon, Portugal

####### **Correspondence:** Rita Canaipa

*BMC Proceedings 2023,* **17(9):**O16

According to one of the most recent approaches to pain research, the Bayesian Brain Hypothesis (BBH), the brain functions as an "inferential machine," making continuous predictions about the internal and external world to better cope with its challenges. The perception of pain ("posteriors" in the BBH terminology) thus results from (1) the ascending sensory signals ("likelihood") and (2) the expectations and conditionings ("priors"), which are based on the previous experiences of the individual. This reciprocal action between the descending priors and the ascending likelihood is constantly weighed according to the individual's confidence in each factor. It has been proposed that chronic pain may be related to biased processing towards the priors. Migraine is a debilitating lifelong pain condition in which disease activity, measured by the frequency and intensity of migraine attacks, oscillates over time. Despite the growing research on this condition the reasons why some patients significantly improve after medication while others do not respond to the generally used pharmacological approach are unclear. Accordingly, the current study aims to use the BBH framework to study the patients' profile and response to acute treatment in episodic and chronic migraine.

Women diagnosed with migraine with or without aura divided into four groups by headache frequency (episodic and chronic) and response to usual attack treatment (consistently insufficient and consistently adequate) were enrolled. They underwent two experimental pain paradigms aiming to measure each component of the BBH: The Focused Analgesia Selection Test (FAST), measuring the within-subject variability in pain intensity reports, and a pain-cued conditioning task assessing acquisition, extinction, and placebo response. Clinical, cognitive, and emotional data were assessed via questionnaires. Differences in the FAST outcome measures (R2 and ICC) and the average conditioned pain intensity are calculated and compared for all patients with adequate and inadequate responses to acute migraine treatment, and controls. We hypothesize that patients that are non-responsive to medication might have lower within-subject variability of pain intensity reports and demonstrate difficulties in the extinction of previous pain conditioning. Preliminary data from healthy controls indicates that, at least in healthy individuals, there are no relations between the two measures, but anxiety and expectations are related to conditioned pain intensity. Further results will be presented and discussed.

#### O17 - DemenPrev: Adaptation of a multidomain intervention for dementia prevention to the Portuguese Context

##### Maria Vânia Silva Nunes^1^

###### ^1^ Universidade Católica Portuguesa, Institute of Health Sciences, Center for Interdisciplinary Research in Health

####### **Correspondence:** Maria Vânia Silva Nunes (mnunes@ics.lisboa.ucp.pt)

*BMC Proceedings 2023,* **17(9):**O17

Dementia is considered a public health priority by WHO. The possibility of its prevention is increasingly recognized. There is a window of opportunity to intervene in risk factors from middle age, possibly preventing at least 40% of the risk. This high prevention potential may be even more significant in low- and middle-income countries. There are many modifiable lifestyle factors, particularly those focused on vascular aspects. In this scenario, aggravated by the effects of the COVID-19 pandemic on this age group, it is a priority to explore various approaches for the primary and secondary prevention of dementia. DemenPrev aims to develop an intervention methodology for dementia prevention in the Portuguese context, comparable to international interventions, and to test its feasibility, both in terms of intervention and in terms of some of the outputs, in an innovative area of enormous potential relevance. As far as recipients are concerned, we hope to maintain or even improve cognitive, biomedical, and motor fitness parameters. We hope to leave intervention capacity, involving and training technicians whenever possible. Integration in the international network of reference in the area, World-Wide-FINGERS Network and their guidance in planning, harmonizing methods and application, helps to align the study with the international standards for dementia prevention.

In this communication we intend to present the final protocol that resulted from the adaptation of the methodology to the Portuguese context, as well as the adaptation of the intervention to the expected duration of 6 months of intervention of the feasibility study.

#### O18 - Research on spirituality in health: Internationalization, integrality, innovation, and implementation

##### Sílvia Caldeira^1^

###### ^1^ Institute of Health Sciences, Universidade Católica Portuguesa, Lisbon, Portugal

####### **Correspondence:** Sílvia Caldeira (scaldeira@ucp.pt)

*BMC Proceedings 2023,* **17(9):**O18

**Background**

From a multidisciplinary perspective, this project – *Spirit in Health* - arises from a nursing background, the holistic approach to patients’ responses toward health conditions or life transitions, across the lifespan, based on dignity-preserving care. This research project explicitly concerns spirituality, a critical dimension of life and, in times of illness, defined as a dynamic and individual dimension related to the search for meaning, transcendence, and connectedness, regardless of being religious or non-religious.

**Case report**

This research project is designed in five dimensions: A (assessment and diagnosis), E (education and theory), I (Interventions), O (Outcomes), and U (Using resources and management). Specific goals of each dimension are regularly defined and updated according to new dissertations and international evidence. In this communication, the rationale, theoretical background, and structure will be disclosed, based on the “four I’s” of the project, as follows: internationalization – all international networking will be listed in terms of expertise for advisory, collaboration in funded or nonfunded projects, and publication outputs; integrality – this project merges two critical areas within the Catholic University of Portugal (CUP), such as spirituality and health and, in this regard, the collaborative projects and other opportunities for collaboration among different faculties, research centers, projects, or researchers will be listed opening new paths for the future, aiming at responding to the strategic plan of CUP, mission, and values, and strengthening funding; innovation – spirituality is not innovative in health. Still, the need to work on new ways of understanding spirituality and implementing it requires innovative approaches. Some outputs related to this critical topic will be listed, which are getting international attention; implementation – four implementation examples will be displayed: three in education (one in Portuguese and two international) and another in a Portuguese clinical setting.

**Conclusion**

This project aims to nurture a broader and interdisciplinary project at Católica that may respond to the mission and create value in research, teaching, and implementation.

#### O19 - Diagnosis and risk prevention of Covid 19 in homeless people in Lisbon

##### Amélia Simões Figueiredo^1,2^, Ana Resende^2^, Cândida Ferrito^2^, Sérgio Deodato^1,2^, João Neves-Amado^1,3^, Dina Manso^4^, António Almeida^1,5^, Amélia Feliciano^5^, Nuno Rosa^1,6^, Marlene Barros^1,6^

###### ^1^ Centro de Investigação Interdisciplinar em Saúde, Universidade Católica Portuguesa, Lisboa, Portugal; ^2^ Instituto de Ciências da Saúde, Universidade Católica Portuguesa, Lisboa, Portugal; ^3^ Instituto de Ciências da Saúde, Universidade Católica Portuguesa, Porto, Portugal; ^4^ Núcleo de Planeamento e Intervenção Sem-Abrigo, Lisboa, Portugal; ^5^ Faculdade de Medicina, Universidade Católica Portuguesa, Sintra, Portugal; ^6^ Faculdade de Medicina Dentária, Universidade Católica Portuguesa, Viseu, Portugal

####### **Correspondence:** Amélia Simões Figueiredo (simoesfigueiredo@ucp.pt)

*BMC Proceedings 2023,* **17(9):**O19

**Background**

The study is part of a broader research project – Public Bathouse Nursing - dedicated to the study of vulnerable populations that use the Public Bathouse in the city of Lisbon [1]. When SARS-CoV-2 pandemic situation started, structures for homeless people, at that time counted as 3029, were reinforced in Lisbon city.

These people do not manage health autonomously, thus requiring definition of strategies for testing and early diagnosis, to promote isolation and quick endorsementto Portuguese National Health Service.

The project was approved by the Health Ethics Committee of the Regional Health Administration of Lisbon and Tagus Valley (Opinion 2776/CES/2021).

**Materials and methods**

Tracking SARS-CoV-2 lab tests were performed to the people from Temporary Housing Centers, Social Emergency Housing Centers and to those who, being homeless, were identified by street technical teams.

Our objectives were the detection of SARS-CoV-2 in the homeless people using the Public Bathhouse of Alcântara and of 10 structures of Planning and Intervention Centers in homelessness, such as street teams, temporary emergency shelters and cafeterias, promoting early detection and adequate endorsement of positive cases.

The process of collection of spittle was performed between November 29^th^,2021and February 7^th^, 2022.

From the initial 455 samples collected, 59 were annulled, due to poor quality and quantity. An error of 3,6% was considered, with a confidence level of 95,0%, thus making viable samples n= 396.

**Results**

Those 396 tracked people revealed an incidence rate of the disease of 2%, while, during the same period, the whole country rounded 18%. A study carried out in the United States of America did not find statistically significant relevance between the values of the incidence of the disease COVID-19 among homeless and non-homeless [2]. On the other hand, a Danish study revealed that the homeless population seems not to have been affected by COVID-19 in the first wave [3]. The positive cases of COVID-19 who were lodged temporarily in Social Emergency Housing Centers, were isolated there, upon indication of Regional and Local Authorities, that were properly informed.

Positive cases of homelessness people identified and tracked on the streets, were endorsed to the Rear Support Structure, to guarantee appropriate isolation and dignified convalescence.

**Conclusions**

The conclusion was that the incidence rate of COVID-19 in homeless people is below general population in Portugal.

**References**

1. Simões-Figueiredo, A., Seabra, P., Sarreira-Santos, A., Vollrath, A., Medeiros-Garcia, L., Vidal, T., & Neves-Amado, J. .Nursing Consultation in a Public Bathhouse: a community resource for the vulnerable population in a European Capital. Issues in Mental Health Nursing. 2019; 40.1: 28-32.

2. Keller, M., Shreffler, J., Wilmes, K., Polites, A., &Huecker, M.. Equal incidence of COVID-19 among homeless and non-homeless ED patients when controlling for confounders. The American journal of emergency medicine. 2022; 53: 286-e5-286-e7.

3. Storgaard, S. F,Eiset, A. HAbdullahi, F., &Wejse, C. . First wave of COVID-19 did not reach the homeless population in Aarhus. Dan Med J. 2020; 67.12: 1-7.

## Poster Presentations

### Session 1 - Translational Care

#### P1 - Executive functioning training in typically developing adolescents: data review from the last 10 years

##### Jorge Amorim^1^, Soraia Saramago^1^, Joana R. Rato,^1^ and Alexandre Castro-Caldas^1^

###### ^1^ Center for Interdisciplinary Research in Health, Universidade Católica Portuguesa, Lisboa, Portugal

####### **Correspondence:** Jorge Amorim (s-jomiamorim@ucp.pt)

*BMC Proceedings 2023,* **17(9):**P1

**Background**

Executive functions (EF) are top-down cognitive processes that affect different life aspects, such as academic success, health management, and, at a last level, public safety. If, on the one hand, literature accumulated data about the possibility of training EF, on the other, adolescence opens a window of opportunity for intervention due to brain, mind-body, and social transformations. Researching effective ways to promote EF in adolescents has a scientific and social value, which motivated this review work.

**Methods**

Through a systematic review, we highlighted the evidence of training EF during adolescence and searched for connections with academic success. Our search gathers knowledge about 1) the tasks (computerized and non-computerized) used to improve EF in adolescents, 2) the program’s effectiveness, and 3) the conditions and settings of training EF.

**Results**

We reviewed studies between 2011 and 2021 across six databases to search for empirical studies with a control group that studied at least one core EF training in typical development adolescents (13-19 y). We only considered peer-review papers published in English, with more than one training session and more than 8 participants. From a pool of 4.002, 14 articles were included in the final analysis. As the main results, we highlight that no single training program was repeated, and measurements vary across multiple tasks and self-reports. The effectiveness lies in low and medium, but no study registered long-duration effects. Computerized training programs have the potential to measure with low bias; however, the only comparison study shows that non-computerized training got promising results.

**Conclusions**

Evidence points to the need for more robust evidence in EF training for adolescents with typical development. Future research should follow open science methodology (i.e., registration of protocols and interventions; open datasets) to reinforce clarity about EF theoretical framework and (non)standardized outcome measures options.

**Keywords**

Executive functions, training, adolescents, inhibitory control, working memory, cognitive flexibility, academic achievement.

#### P2 - Liposomal delivery of repurposed antiviral drug saquinavir to macrophages as a host-directed therapy for tuberculosis

##### Manoj Mandal^1#^, David Pires^1,2#^, Jacinta Pinho^3^, Maria João Catalão^1^, António José Almeida^3^, José Miguel Azevedo-Pereira^1^, Maria Manuela Gaspar^3^ and Elsa Anes^1^

###### ^1^ Host-Pathogen Interactions Unit, Research Institute for Medicines - iMed-ULisboa, Faculty of Pharmacy, Universidade de Lisboa, Lisbon, Portugal; ^2^ Universidade Católica Portuguesa, Católica Medical School, Center for Interdisciplinary Research in Health, Rio de Mouro, Sintra, Portugal; ^3^ Advanced Technologies for Drug Delivery, Research Institute for Medicines - iMed-ULisboa, Faculty of Pharmacy, Universidade de Lisboa, Lisbon, Portugal

####### **Correspondence:** Elsa Anes (eanes@ff.ulisboa.pt)

*BMC Proceedings 2023,* **17(9):**P2

^#^ These authors contributed equally to this work

*Mycobacterium tuberculosis* (Mtb) latently infects approximately a quarter of the world’s population and 10 % of these will develop the disease tuberculosis. Mtb infects macrophages, manipulating the proteolytic mechanisms, particularly, by decreasing the expression and activity of lysosomal cathepsins. Consequently, Mtb survives and even replicates inside macrophages concomitant with poor priming of the adaptive immune response. Our group found that the protease inhibitor used in antiretroviral therapy for HIV infection, saquinavir (SQV), restores and further improves the overall activity of cathepsins in Mtb-infected macrophages and more specifically, that of cathepsin S [1]. In this study, we tested the incorporation of SQV in liposomes to establish an improved delivery method for SQV to human monocyte-derived macrophages. Using fluorophore-tagged liposomes we demonstrated the efficiency of SQV-loaded liposome internalization by human macrophages. Additionally, using a general fluorescent substrate of human cathepsins we could observe improved proteolytic activity in treated macrophages. When applying this treatment to Mtb-infected macrophages these effects resulted in better control of the infection. Furthermore, liposomal delivery of SQV reduced the cytotoxicity of the treatment and allowed the usage of higher concentrations without impacting cell viability. By using this strategy, we overcame the cathepsin activity blockade that is induced by the pathogen [2]. The results further demonstrate the efficacy of SQV-loaded liposomes to help control infections by Mtb clinical strains susceptible or resistant to the current antibiotic therapy. Our results suggest the use of liposomal delivery of SQV as a potential complementary therapy against Mtb infection.

Human monocytes were isolated from buffy-coats of healthy human donors provided by the National Blood Institute (Instituto Português do Sangue e da Transplantação, IP, Lisbon, Portugal).

This study was supported by grants from National Foundation for Science, FCT (Fundação para a Ciência e Tecnologia – Portugal), PTDC/SAU-INF/28182/2017 to EA and EXPL/SAU-INF/0742/2021 to DP and the fellowship 2021.07978.BD to MM.

**References**

1. Pires D, Valente S, Calado M, Mandal M, Azevedo-Pereira JM, Anes E. Repurposing saquinavir for host-directed therapy to control *Mycobacterium tuberculosis* infection. Front Immunol. 2021 Mar 26;12:647728. doi: 10.3389/fimmu.2021.647728.

2. Pires D, Marques J, Pombo JP, Carmo N, Bettencourt P, Neyrolles O, et al. Role of cathepsins in *Mycobacterium tuberculosis* survival in human macrophages. Sci Rep. 2016 Aug;6(1):32247. doi: 10.1038/srep32247.

#### P3 - Development of a new mRNA vaccine platform for tuberculosis

##### Laura Matarazzo^1,2^, Laura Taina-González^3,4^, Ricardo Pinheiro^2^, David Pires^1,2,5^, María de la Fuente^4,6,7^, Paulo J. G. Bettencourt^1,2^

###### ^1^ Center for Interdisciplinary Research in Health, Universidade Católica Portuguesa, Lisboa, Portugal; ^2^ Universidade Católica Portuguesa, Faculty of Medicine, Rio de Mouro, Portugal; ^3^ Universidad de Santiago de Compostela (USC), 15782 Santiago de Compostela, Spain; ^4^ DIVERSA Technologies, Santiago de Compostela, Spain; ^5^ Host-Pathogen Interactions Unit, Research Institute for Medicines, iMed-ULisboa, Faculty of Pharmacy, Universidade de Lisboa, Lisboa, Portugal; ^6^ Nano-Oncology and Translational Therapeutics Group, Health Research Institute of Santiago de Compostela (IDIS), SERGAS, Santiago de Compostela, Spain; ^7^ Cancer Network Research (CIBERONC), Madrid, Spain

####### **Correspondence:** Paulo J. G. Bettencourt (pbettencourt@ucp.pt)

*BMC Proceedings 2023,* **17(9):**P3

**Background**

Tuberculosis (TB), caused by *Mycobacterium tuberculosis* (*M.tb*), is the first cause of death by an infectious disease worldwide, killed 1.6 million people in 2021. Bacillus Calmette-Guerin (BCG) is the only approved vaccine for TB to date. However, while BCG is effective in preventing severe forms in children, its efficacy in adults is inconsistent and it does not prevent transmission, highlighting the need for new vaccine development [1]. The recent success of COVID-19 vaccines raised the interest for mRNA-based vaccines, as they are effective, safe and easy to produce. This project aims to develop a new mRNA vaccine platform for TB, based on mRNA coding for antigenic peptides from BCG and *M.tb* identified by immunopeptidomics [2], and formulated with a patented technology of lipid nanoemulsions (NE) (WO2019138139A1), adapted for efficient intracellular delivery of mRNA [3].

**Materials and methods**

We tested different prototypes of NE-mRNA formulations, coding for EGFP, *in vitro.* Human alveolar basal epithelial cells (A549), human monocytic cells (THP-1), and primary human monocyte-derived macrophages, were transfected with NE-mRNA formulations. Transfection efficiency was assessed by measuring the percentage of transfected cells, and the intensity of GFP fluorescence. The cytotoxicity of the formulations was evaluated using AlamarBlue, and by 7-AAD viability staining.

**Results**

*In vitro* preliminary data using EGFP-mRNA-NE formulations indicate that NE formulations can efficiently deliver mRNA and induce expression of the encoded protein in different cell types, with low cytotoxicity.

**Conclusions**

The NE technology presented here is safe, stable, and can efficiently deliver mRNA to various cell types. Selected NE formulations will be used as a carrier for a new vaccine candidate against TB, based on mRNA encoding relevant antigenic peptides. These will be tested in mice for safety, immunogenicity, efficacy and dose optimization in order to generate an effective and sustained humoral and cellular immune response against TB. The mRNA vaccines are rapid and relatively simple to produce. The vaccine platform described here could be adapted to develop vaccines against other infectious diseases, particularly to quickly respond to emerging pathogens.

**Ethical statement**

Human monocyte-derived macrophages were obtained from buffy-coats of healthy donors provided by the national blood institute (Instituto Português do Sangue e da Transplantação, Lisbon, Portugal).

**References**

1. Bettencourt PJG, et al. 100 years of the Bacillus Calmette-Guérin vaccine. Vaccine. 2021 Dec 8;39(50):7221-7222.

2. Bettencourt P, et al. Identification of antigens presented by MHC for vaccines against tuberculosis. NPJ Vaccines. 2020;5(1):2.

3. Taina-González L, de la Fuente M. The Potential of Nanomedicine to Unlock the Limitless Applications of mRNA. Pharmaceutics. 2022 Feb 21;14(2):460.

#### P4 - Implementation of a pre-Good Laboratory Practice management system for academic research

##### Ricardo Pinheiro^1^, Cloé Abreu^1^, Paulo J. G. Bettencourt^1,2^

###### ^1^ Universidade Católica Portuguesa, Faculty of Medicine, 2635-631 Rio de Mouro, Portugal; ^2^ Universidade Católica Portuguesa, Center for Interdisciplinary Research in Health, 1649-023 Lisboa, Portugal

####### **Correspondence:** Paulo J. G. Bettencourt (pbettencourt@ucp.pt)

*BMC Proceedings 2023,* **17(9):**P4

The implementation of quality control procedures, at an academic laboratory, relies on a system that flows information to scientists, staff, and students in a clear and accountable manner.

The organization and implementation of new methodologies, in a new laboratory, implies the definition of a work culture and structure from inception to completion. Establishing and maintaining a new work philosophy is demanding and requires constant and close supervision of all laboratory actions. Particularly, when the methods are innovative and require a significant change of work culture from users.

By establishing a system that standardizes common laboratory protocols to facilitate training while simultaneously tracking progress, we successfully implemented a pre-Good Laboratory Practices (pre-GLP) facility at the Faculty of Medicine of the Universidade Católica Portuguesa (FM).

The pre-GLP system is an adaption of the system adopted by the Jenner Institute, University of Oxford. Briefly, the new users are trained on Standard Operations Procedures (SOP), provided by a competent user. Once training is successfully completed, the user is approved and qualified as competent user. All training actions are recorded in the researcher’s internal record. The internal records are internally verified by the laboratory manager, and laboratory director, and externally audited.

The SOPs are regularly updated and improved to reflect any significant updates on procedures, equipment, and reagents. Updated SOP´s are reassessed and follow the pipeline of approval. Implementation of this laboratory management system is a step forward in quality assurance and standardization of methodologies towards good laboratorial practices, increased health, and safety, and quality data production.

Finally, the implementation of this quality assurance method at the FM, provides an additional layer of health and safety protection for users, simultaneously assuring reproducibility and reliability of protocols across the campus.

#### P5 - Mass spectrometry-based identification of peptides presented by major histocompatibility complex in macrophages

##### Hugo Mateus^1,2,3^, Ricardo Pinheiro^1^, Hugo M. Santos^3,4,5^, Paulo J. G. Bettencourt^1,6^

###### ^1^ Universidade Católica Portuguesa, Faculty of Medicine, 2635-631 Rio de Mouro, Portugal; ^2^ NOVA School of Science and Technology, FCT NOVA, Universidade NOVA de Lisboa, 2829-516, Caparica, Portugal; ^3^ BIOSCOPE Research Group, LAQV-REQUIMTE, Chemistry Department, NOVA School of Science and Technology, FCT NOVA, Universidade NOVA de Lisboa, 2829-516, Caparica, Portugal; ^4^ PROTEOMASS Scientific Society, Madan Park, 2829-516, Caparica, Portugal; ^5^ Department of Pathology, University of Pittsburgh Medical Center, Pittsburgh, PA, USA; ^6^ Universidade Católica Portuguesa, Center for Interdisciplinary Research in Health, 1649-023 Lisboa, Portugal

####### **Correspondence:** Paulo J. G. Bettencourt (pbettencourt@ucp.pt); Hugo M. Santos (hmsantos@fct.unl.pt)

*BMC Proceedings 2023*, **17(9):**P5

Immunopeptidomics is a field of research that has progressed in the last years due to advances in sophisticated analytical techniques based on mass spectrometry and bioinformatics. The ability to identify molecules to the extent of a single ion led to a step forward in immunopeptidomics. Mass spectrometry enables the identification of thousands of peptide sequences in a single sample, thus providing large-scale reliable information. The immunopeptidome is the entire group of peptides presented by the major histocompatibility complex Class-I (MHC-I), at the surface of all nucleated cells and Class II, at the surface of professional antigen presenting cells. The MHC-bound peptides are recognized by T cells and constitute the immunological synapse, leading to the initiation of the adaptive immune response. Under pathological conditions, peptides originating from the proteolysis of pathogen proteins are presented to the cells of the host immune system via MHC. Thus, the identification of pathogen peptides through immunopeptidomics is an unbiased method for understanding the generation of adaptive immune responses against pathogens.

Here we describe the establishment of a new mass spectrometry-based immunopeptidomics platform for peptide identification in physiological and pathological conditions. Using the macrophage cell line with THP-1, with a known HLA-type, we were able to identify a total of 2913 unique MHC-I bound peptides. The peptide length distribution, NetMHCpan-4.1 rank affinity, and best match HLA binding allele for each peptide will be presented.

Finally, identifying MHC-I and MHC-II peptides under physiological and pathological conditions could uncover the most relevant peptides able to stimulate the right type of T-cell response for vaccine design and development.

#### P6 - CD137 drives therapeutic resistance to JAK inhibition therapy in Myeloproliferative Neoplasms

##### Bruno Martins^1^, António Medina Almeida^1,2^, Bruno António Cardoso^1^

###### ^1^ Universidade Católica Portuguesa, Faculdade de Medicina, Centro de Investigação Interdisciplinar em Saúde, Lisbon, Portugal; ^2^ Hospital da Luz, Lisbon, Portugal

####### **Correspondence:** Bruno António Cardoso Bruno António Cardoso

*BMC Proceedings 2023,* **17(9):**P6

The BCR-ABL-negative myeloproliferative neoplasms (MPN) are clonal myeloid malignancies that rely on constitutive JAK-STAT signaling as a consequence of the JAK2^V617F^ mutation. However, despite the recent advances in understanding MPN pathophysiology and the efficacy of JAK inhibitors in the clinical practice, bone marrow transplantation remains the only curative option. Unfortunately, resistance to chemotherapy is a frequent event in myeloid malignancies and the bone marrow (BM) microenvironment provides the perfect protective milieu for leukemic cells to thrive and proliferate. Research from our own group demonstrated that the BM protects from the cytotoxic effects of JAK inhibition (Ruxolitinib) in MPN cells, and such effects rely on the activation of PI3K-Akt and JNK/SAPK signaling networks.

MPN patient derived cell lines (SET-2 and HEL) were incubated cultured *in vitro* (no stroma) alone, with HS-5 bone marrow cell line and with HS-5 conditioned media medium in the presence of Ruxolitinib and CD137 neutralizing antibody. The cellular viability was analyzed by staining with Annexin-V/7-AAD and CD45-APC (to distinguish MPN cells from the HS-5 cells) staining. Furthermore, cells were also stained with a CD137-PE antibody and lysed for RNA extraction. cDNA was synthesized and gene expression evaluated by quantitative real-time polymerase chain reaction (qPCR) and normalized to the expression levels of *HPRT1* gene.

Interestingly, in a screen to search for novel modulators of BM-mediated protection to JAK inhibition in MPN disease we identified the *TNFRSF9* gene. The *TNFRSF9* gene encodes for the CD137 receptor that receptor belongs to the Tumor Necrosis Factor Receptor Superfamily (TNFRSF) and is involved in tissue homeostasis by regulating inflammation. We found that the contact of MPN cells with BM in the presence of Ruxolitinib upregulated the *TNFRSF9* transcript levels and the surface expression of the CD137 receptor. Importantly, the inhibition of the CD137 receptor with a neutralizing antibody dampened the BM protective effect to the cytotoxic action of Ruxolitinib.

Overall, our preliminary results identify the CD137 death receptor as a putative novel regulator BM-mediated protection in the context of MPN disease and we are currently intensifying our studies to further exploit the therapeutic applications of this receptor as well as the molecular mechanisms behind it.

#### P7 - Evaluation of Extruded Material in Furcation Perforation Repair with Micro-computed Tomography

##### Miguel Cardoso^1^, Rita Noites^1^, Vitor Correlo^2^, Carlos Viegas^3^

###### ^1^ Universidade Católica Portuguesa, Faculty of Dental Medicine, Center for Interdisciplinary Research in Health, Viseu, Portugal; ^2^ 3B's Research Group-Biomaterials, Biodegradables and Biomimetics, Department of Polymer Engineering-School of Engineering University of Minho, Gandra, Portugal; ^3^ Department of Veterinary Sciences University of Trás-os-Montes e Alto Douro, Vila Real, Portugal

####### **Correspondence:** Miguel Cardoso (mabcardoso@ucp.pt)

*BMC Proceedings 2023,* **17(9):**P7

**Background**

Furcation perforations are pathological conditions of complex treatment and, currently, bioceramics are good options for furcation perforations repair. The aim of this study was to compare the volume of extruded material with micro-computed tomographic (microCT) after Furcation Perforation (FP) repair with Biodentine (BDT) or ProRoot MTA (prMTA) in dogs’ teeth.

**Materials and methods**

Forty dogs’ teeth were divided into 2 groups: prMTA (n=20, FP repaired with ProRoot MTA), BDT (n=20, FP repaired with Biodentine). All animal procedures were approved by the institutional Ethical Committee and conformed with the ethical guidelines and regulations of the national Directorate-General for Food and Veterinary (Process number 0421/000/000/2014). The animals were euthanized after 4 months. The volume of extruded material was quantified using microCT images.

Statistical analysis was performed using independent-samples t-test in SPSS™. All differences were considered significant at *P*≤0.05.

**Results**

Total volume of extruded material was significantly lower in BDT group than in prMTA group (BDT: 1.42±0.80mm3; prMTA: 2.27±1.67mm3; *P*=0.049).

In both test material groups, microCT showed continuity between the extruded repair material and the surrounding bone.

Along with the study’s included outcomes, further evaluation of microCT images allowed the identification of new mineralized tissue bridges over the remaining radicular pulp tissue in specimens of both test groups.

**Conclusions**

The greater amount of extruded material found for prMTA group is consistent with its lengthier setting time, which may contribute to the unintended compaction of the unset material into the furcation defect. Even though Biodentine presented lesser extrusion, a concomitant histologic study revealed similar results concerning mineralized tissue formation.

**Keywords**

Biodentine; Endodontics; Furcation perforation; *in vivo*; MTA.

#### P8 - Marine fungi exhibit antimicrobial activity against human oral pathogens

##### Bruna L. Correia^1,2^, Daniela Devesas^2^, Rita Noites^1,2^, Ana T.P.C. Gomes^1,2^, Ana Cristina Esteves^3^, Artur Alves^3^, Ana Sofia Duarte^1,2^

###### ^1^ Universidade Católica Portuguesa, Center for Interdisciplinary Research in Health, Viseu, Portugal; ^2^ Universidade Católica Portuguesa, Faculdade de Medicina Dentária, Viseu, Portugal; ^3^ CESAM & Departamento de Biologia, Universidade de Aveiro, Aveiro, Portugal

####### **Correspondence:** Bruna L. Correia (bcorreia@ucp.pt)

*BMC Proceedings 2023,* **17(9):**P8

The emergence of resistance to antibiotics and antimycotics has become a challenge in the treatment of infectious diseases, including infections of the oral cavity. Marine fungi are a source of novel biologically active compounds, namely in what concerns the development of antimicrobial and anticancer solutions. Our study aimed to test the antimicrobial activity and the cytotoxicity of the extracts of the two recent identified species of marine fungi, *Penicillum lusitanum* and *Aspergillus affinis. Candida* spp. and *Enterococcus faecalis* isolated from oral pathologies were included to evaluate the antimicrobial potential of the marine fungi by the disk diffusion assay. The cytotoxicity of the effective concentrations of the extract was tested using the Vero cell line (ECACC 88020401, African Green Monkey Kidney cells, GMK clone), according to the ISO 10993-5. The extracts of *P. lusitanum* and *A. affinis* were active against *C. albicans* and *E. faecalis*, respectively. *Penicillum lusitanum* active extracts are non-cytotoxic, in contrast to *A. affinis* extracts that showed high cytotoxic effects on Vero cells, for all concentrations tested. The results on the biological characterization of the *P. lusitanum* extract are promising and support the development of new disinfecting solutions that may be used during root canal therapy cleaning and shaping.

**Funding**

This work is financially supported by National Funds through FCT – Fundação para a Ciência e a Tecnologia, I.P., under the CIIS (UIDB/04279/2020) and CESAM (UIDP/50017/2020+UIDB/50017/2020+LA/P/0094/2020) projects, and by the Programa Operacional Capital Humano e Fundo Social Europeu (FSE), under the project Indig (POCH-02-53I2-FSE-000025). Bruna L. Correia thanks the UCP for the Research grant, under the project Indig. Thanks are also due to FCT and UCP for the CEEC institutional financing of Ana T.P.C. Gomes (CEECINST/00137/2018/CP1520/CT0022), and Ana Sofia Duarte (CEECINST/00137/2018/CP1520/CT0013).

#### P9 - Antimicrobial properties and bioactivity potential of smart nanoparticles for dental applications

##### Bruna L. Correia^1,2^, Moslem Malekshiri^3^, Maria Bartolomeu^1,2^, Virgília Silva^3^, Ana Oliveira^2^, Rita Noites^1,2^, Miguel Cardoso^1,2^, Karina Mendes^1,2^, Ana T.P.C. Gomes^1,2^, Ana Sofia Duarte^1,2^

###### ^1^ Universidade Católica Portuguesa, Center for Interdisciplinary Research in Health, Viseu, Portugal; ^2^ Universidade Católica Portuguesa, Faculdade de Medicina Dentária, Viseu, Portugal; ^3^ CESAM & Departamento de Biologia, Universidade de Aveiro, Aveiro, Portugal

####### **Correspondence:** Bruna L. Correia (bcorreia@ucp.pt)

*BMC Proceedings 2023,* **17(9):**P9

Tooth decay is one of the greatest causes of tooth loss in the world. This not only affects the patient's quality of life but also carries an economic burden associated with the need for multiple reinterventions. Endodontic treatment aims to preserve teeth by cleaning, disinfecting and filling/sealing the root canal. Despite the high success rate of endodontic treatment, failures do occur in a large number of cases. Several new biomaterials for dentistry have been developed, however their bioactivity is often misunderstood. Our work focuses on the biological characterization of novel bioactive glass nanoparticles, including the evaluation of their antimicrobial and biocompatibility properties. *Candida albicans* (ATCC 11225) and *Enterococcus faecalis* (ATCC 29212) were included to evaluate the antimicrobial potential by the drop plate method [1]. The cytotoxicity was tested using the MC3T3-E1 cell line, through the resazurin reduction assay. The novel bioactive glass nanoparticles demonstrated antimicrobial activity against *C. albicans* and *E. faecalis*, being able to inhibit their growth but also, in some incubation times, decreased the survival of these microorganisms. After 24 h of incubation of MC3T3-E1 osteoblast cells with bioactive glass nanoparticles conditioned medium, around 48% cell viability was achieved. These novel bioactive glass nanoparticles have shown promising properties which may find applications on different areas of clinical dentistry.

**Funding**

This work is financially supported by National Funds through FCT – Fundação para a Ciência e a Tecnologia, I.P., under the UIDB/04279/2020 and CESAM (UIDP/50017/2020+UIDB/50017/2020+LA/P/0094/2020) projects, and by Programa Operacional Capital Humano e Fundo Social Europeu (FSE), under the project Indig (POCH-02-53I2-FSE-000025). Maria Bartolomeu and Bruna L. Correia thank the UCP for the Junior Researcher position and the Research grant, respectively, under the project Indig. Thanks are also due to FCT and UCP for the CEEC institutional financing of Ana T.P.C. Gomes (CEECINST/00137/2018/CP1520/CT0022), Karina Mendes (CEECINST/00070/2021-CIIS-Júnior) and Ana Sofia Duarte (CEECINST/00137/2018/CP1520/CT0013).

**Reference**

1. Correia BL, Gomes ATPC, Noites R, Ferreira JMF, Duarte AS. New and Efficient Bioactive Glass Compositions for Controlling Endodontic Pathogens. Nanomaterials. 2022; 12(9):1577.

#### P10 - Should thermoplastic resins be used in removable dentures?

##### Beatriz Teixeira^1^, Helena Salgado^1^, André Correia^1,2^, Patrícia Fonseca^1,2^

###### ^1^ Universidade Católica Portuguesa, Faculty of Dental Medicine (FMD), Viseu, Portugal; ^2^ Universidade Católica Portuguesa, Center for Interdisciplinary Research in Health (CIIS), Viseu, Portugal

####### **Correspondence:** Patrícia Fonseca (pafonseca@ucp.pt)

*BMC Proceedings 2023,* **17(9):**P10

**Background**

Removable dentures are the most popular rehabilitation treatment for edentulous patients. However, due to the presence of retentive elements and metallic structures in the aesthetic areas, several patients have reported both aesthetic and psychological problems. In addition, this type of dentures has low ductility and, therefore, low resistance to fracture. These limitations favored the development of flexible resins for use in conventional oral rehabilitation, which provide greater comfort and aesthetics for the patient.[1-3]

The aim of this investigation is to evaluate if the fabrication of denture bases with thermoplastic flexible resins provides superior mechanical and physical results in comparison with conventional acrylic resin (polymethylmethacrylate).

**Materials and methods**

After registering the research protocol in PROSPERO, the same was conducted using the PubMed/Medline®, Cochrane® Library, Web of Science® and Scopus® databases, where a combination of MeSH and free text terms were combined with Boolean operators AND and OR. The selection of articles was carried out by two independent investigators, according to the PRISMA flowchart, and the agreement was evaluated by Cohen's kappa coefficient, being later analyzed, and evaluated according to the established inclusion and exclusion criteria.

**Results**

In the 10 analyzed studies in this systematic review, 431 specimens were evaluated, being 310 of flexible thermoplastic resin and 121 of conventional acrylic resin. The studies included are in vitro and compare the mechanical properties and physical characteristics between the different types of resin. The production of removable prosthetic bases in flexible thermoplastic resin presents excellent mechanical results, but combined with poor physical characteristics, may not present superior long-term results, compared to conventional acrylic resin.

**Conclusions**

Given the results obtained, the option for these materials may not yet be an alternative to polymethylmethacrylate. More research is needed to optimize and validate these materials for intra-oral use.

**References**

1. Carr A, Brown DT. McCracken’s Prótese Parcial Removível. 12nd Edition. Rio de Janeiro: Elsevier Ed; 2012. p.400.

2. Wada J, Fueki K, Yatabe M, Takahashi H, Wakabayashi N. A comparison of the fitting accuracy of thermoplastic denture base resins used in nonmetal clasp dentures to a conventional heatcured acrylic resin. Acta Odontol Scand. 2015;73(1):33-37.

3. Shaghaghian S, Taghva M, Abduo J, Bagheri R. Oral healthrelated quality of life of removable partial denture wearers and related factors. J Oral Rehabil. 2015;42(1):40-48.

#### P11 - 3D Printed polymethyl methacrylate resins for dentures – where is the evidence?

##### Claúdia Lourinho^1^, Helena Salgado^1^, André Correia^1,2^, Patrícia Fonseca^1,2^

###### ^1^ Universidade Católica Portuguesa, Faculty of Dental Medicine (FMD), Viseu, Portugal; ^2^ Universidade Católica Portuguesa, Center for Interdisciplinary Research in Health (CIIS), Viseu, Portugal

####### **Correspondence:** Patrícia Fonseca (pafonseca@ucp.pt)

*BMC Proceedings 2023,* **17(9):**P11

**Background**

The rehabilitation of edentulous spaces using removable dentures is a viable therapeutic option. The synergy between Dentistry and Informatics allowed the emergence of new technologies, specifically 3D printing, which led to the development of new materials to be used. Therefore, it is essential and relevant to study the properties of this materials, and particularly, its mechanical characteristics [1-3].

The main aim of this investigation was to access the scientific evidence on the mechanical properties of polymethyl methacrylate resins for 3D printing of removable dentures, comparing with conventional resins.

**Materials and methods**

A systematic review methodology was developed following the PRISMA guidelines. Keywords from natural language and controlled vocabulary were selected to be applied in three databases (PubMed/MEDLINE®, Web of Science-MEDLINE® and EMBASE®) until April 30th, 2022. Research protocol was registered in PROSPERO with ID CRD42022296181. Two researchers selected the studies independently. Quality of the papers was assessed using the checklist for quasi-experimental studies from Joanna Briggs Institute and agreement between examiners was measured using the Cohen's Kappa coefficient. Meta-analysis was performed to the variable flexural strength.

**Results**

Through the research, 93 articles were identified. After excluding duplicates, the inclusion/exclusion criteria were applied to 55 papers, to select them, first by reading the title (n=12), then abstract (n=10) and, finally, the full text (n=8). The 3D printed resin presents, in most studies, lower values of flexural strength and hardness, compared to thermopolymerizable resin. Forest plot indicates that the articles that mention the similarity of flexural strength values in both groups, from a statistical point of view, are not relevant. Regarding impact strength, the studies included in this systematic review point lower values in case of thermopolymerizable resin compared to 3D printing resin.

**Conclusions**

3D printing resins are viable materials for making prosthetic bases but need further clinical research about its performance and longevity.

**References**

1. Pillai S, Upadhyay A, Khayambashi P, Farooq I, Sabri H, Tarar M, Lee KT, Harb I, Zhou S, Wang Y, Tran SD. Dental 3D-Printing: Transferring Art from the Laboratories to the Clinics. Polymers (Basel). 2021;13(1):157.

2. Schweiger J, Edelhoff D, Güth JF. 3D Printing in Digital Prosthetic Dentistry: An Overview of Recent Developments in Additive Manufacturing. J Clin Med. 2021;10(9):2010.

3. Tian Y, Chen C, Xu X, Wang J, Hou X, Li K, Lu X, Shi H, Lee ES, Jiang HB. A Review of 3D Printing in Dentistry: Technologies, Affecting Factors, and Applications. Scanning. 2021; 2021:9950131.

#### P12 - Dentistry Under a New Light: Antimicrobial Photodynamic Therapy as Sustainable Solution for Periodontitis and Periimplantitis Treatment

##### Anna Moura^1,2^, Diogo Esteves^2^, Daniel Andreolli^2^, Ana T. P. C. Gomes^1,2^

###### ^1^ Universidade Católica Portuguesa, Center for Interdisciplinary Research in Health, Viseu, Portugal; ^2^ Universidade Católica Portuguesa, Faculdade de Medicina Dentária, Viseu, Portugal

####### **Correspondence:** Anna Moura (anna@ucp.pt)

*BMC Proceedings 2023,* **17(9):**P12

Oral health conditions can significantly impact on the quality of life. Despite the scientific progress in the understanding of the pathogenesis and oral diseases causes, these are a global public health. Poor oral health results in pain, substandard nutrition, work absence and lowered self-esteem. Chronic oral infection is a proven risk factor for diabetes, heart, and lung disease.

Periodontitis and periimplantitis are oral conditions that have an infection etiology. Despite of the current available techniques used for these diseases’ treatment, none guaranties the total eradication neither prevent (re)infection. It is urgent to find alternative treatments to mitigate these difficulties and improve the diseases’ prognosis. Antimicrobial Photodynamic Therapy (aPDT) arises as an alternative with unique features and presents advantages when compared the use of conventional antimicrobials, showing to be efficient and preventing the development of resistance. aPDT has been extensively studied to treat periimplantitis and periodontitis, but the developed protocols are restricted to phenothiazinium photosensitizers, such as methylene blue, and to the use of red lasers as light source.

It is intended to develop an effective therapeutic approach to treat periimplantitis and periodontitis based on aPDT using porphyrins already approved for clinical and the dental curing light (DCL-available in all dental clinics) as light source.

The *in vitro* photoinactivation assays of periodontopathogens (*E. faecalis* and *C. albicans*) were carried out in PBS, with the disodium salt of Protoporphyrin IX (Proto IX) as photosensitizer and DCL as a light source. The *ex vivo* antimicrobial inactivation of such periodontopathogens were also evaluated under the same aPDT protocol in teeth and dental implants.

*In vitro* assays showed an effective photoinactivation of the periodontopathogens when exposed to different concentrations of Proto IX and with DCL. *Ex vivo* assays in dental implants showed promising results, with high photoinactivations rates of *E. faecalis*. However, probably due to the complex tooth matrix, the aPDT efficiency in teeth was modest.

The aPDT protocol achieved by the combination of Proto IX and DCL showed to be efficient in the inactivation of periodontopathogens. These results open new perspectives for an efficient aPDT protocol development to treat periodontitis and periimplantitis that can be easily implemented in all dental clinics and available to entire population, contributing to the democratization of medical services.

The use of teeth for research was approved by the Comissão de Ética para a Saúde of the Universidade Católica Portuguesa (project CES160).

**Funding**

This work is financially supported by National Funds through FCT – Fundação para a Ciência e a Tecnologia, I.P., under the projects UIDP/04279/2020. Thanks are also due to FCT and UCP for the CEEC institutional financing of Ana Gomes (CEECINST/00137/2018/CP1520/CT0022).

#### P13 - HPV in the oral cavity - interatomic approach

##### Alexandre Bernardo^1^, Maria José Correia^2^, Ana Cristina Esteves^3^, Nuno Rosa^2^

###### ^1^ Universidade Católica Portuguesa, Faculty of Dental Medicine (FMD, Viseu, Portugal; ^2^Universidade Católica Portuguesa, Faculty of Dental Medicine (FMD), Center for Interdisciplinary Research in Health (CIIS), Viseu, Portugal; ^3^ CESAM, Department of Biology, University of Aveiro, Aveiro, Portugal

####### **Correspondence:** Nuno Rosa (nrosa@ucp.pt)

*BMC Proceedings 2023,* **17(9):**P13

Human papillomavirus (HPV) is a common virus that can infect the oral cavity where some types of HPV can cause cancer. However, the molecular aspects related to this infection are not yet fully understood.

This work aims to study the molecular mechanisms through which HPV interacts with the host after infection and its relationship with the clinical manifestations of the infection. This information is used to design a panel of salivary biomarkers associated with HPV infection.

The salivary proteome of HPV-infected individuals was annotated and the SalivaTecDB database was updated. A functional analysis was performed to identify a potential panel of salivary biomarkers, using PANTHER, DAVID and STRING tools. Prediction of PPIs was performed with the OralInt algorithm. Functional analysis of the PPIs was performed using the Cytoscape® and CluGO+CluePedia programs.

The functional analysis of 514 proteins led to the identification of 11 salivary proteins characteristic of HPV associated oral cancer (RPRD2, PSCA, MCM2, MCM5, CDKN2A, BAK1, HSPA1A, HSPA5, HSPA8, TANK, MAP2K1). OralInt predicted a total of 18389 HPV-host interactions, and of these, 447 were high confidence PPIs (Score ≥0.75). The functional analysis of the network allowed the identification of pathways that may be altered after HPV infection: "Parkinson disease", "Lipid and atherosclerosis", "DNA Replication", "Proteasome", "Estrogen signaling pathway".

This study permitted the proposal of 8 salivary biomarkers for oral cancer in patients previously infected with HPV (RPRD2, PSCA, MCM2, CDKN2A, BAK1, HSPA1A, TANK, MAP2K1). The interatomic analysis allowed to elucidate that HPV infection can alter “Pathways” such as “Estrogen signaling pathway”, Lipid and atherosclerosis”, “DNA Replication” and “Proteasome”, potentially associated with cancer.

#### P14 - Proposal of a salivary biomarker panel for oral lichen planus: a bioinformatics approach

##### José Pedro Santos^1^, Ana Cristina Esteves^2^, Nuno Rosa^3^

###### ^1^ Universidade Católica Portuguesa, Faculty of Dental Medicine (FMD, Viseu, Portugal; ^2^ CESAM, Department of Biology, University of Aveiro, Aveiro, Portugal; ^3^ Universidade Católica Portuguesa, Faculty of Dental Medicine (FMD), Center for Interdisciplinary Research in Health (CIIS), Viseu, Portugal

####### **Correspondence:** Nuno Rosa (nrosa@ucp.pt)

*BMC Proceedings 2023,* **17(9):**P14

Lichen planus is a chronic inflammatory, mucocutaneous pathology with possible autoimmune origin. The most described clinical classifications of oral lichen planus are reticular, erosive, bullous, and plaque-like. Although clinical presentation is part of the diagnosis, it is recommended that the diagnosis be confirmed by biopsy and histological confirmation of the lesions and sometimes by biomarkers. However, information to assist in the identification of specific, early and clinically useful biomarker panels and to clarify the molecular aspects of the development and manifestation of the pathology is lacking.

This work aims to propose a set of salivary biomarkers useful for the diagnosis of Oral Lichen Planus.

Oral Lichen Planus salivary proteome was manually annotated and the SalivaTecDB database was updated. A functional analysis was performed to identify a potential panel of salivary biomarkers characteristic of Lichen Planus, using UniProtKB and DAVID tool. A biomarker score was also calculated based on the data resulting from manual annotation and functional analysis of the identified proteins to attribute a robustness to each biomarker.

A total of 136 distinct proteins were identified and annotated. Of these, 42 were associated with at least one biological process related to Oral Lichen Planus, 102 were already associated with one or more pathologies, of which 53 showed association with autoimmune pathologies and 10 with Lichen Planus.

This work proposes a panel of 6 salivary biomarkers, still to be validated, for Oral Lichen Planus: TNF-α, TNFR-2 (or TNFR-1β), IL-1α, IL-6, fibrinogen β-chain and complement component C3.

#### P15 - DNA repair SNPs as biomarkers of therapeutic response in oral cancer patients: a systematic review

##### Bruna Mirahy^2^, Maria José Correia^1,2^, Raquel Silva^1,2^, Luís Silva Santos^1,2^

###### ^1^ Universidade Católica Portuguesa, Center for Interdisciplinary Research in Health, Viseu, Portugal; ^2^ Universidade Católica Portuguesa, Faculdade de Medicina Dentária, Viseu, Portugal

####### **Correspondence:** Luís Silva Santos (lsantos@ucp.pt)

*BMC Proceedings 2023,* **17(9):**P15

**Background**

Radiotherapy, chemotherapy, or their combination remain a mainstay in oral cancer treatment. Such agents introduce genetic lesions in tumour cells to kill them. Non-tumour cells are also affected, generating side effects, e.g., secondary cancers. DNA repair pathways exist that repair such therapy-induced genetic lesions and avoid their fixation as mutations (potentially oncogenic) or the activation of apoptosis. Since most DNA repair genes are polymorphic, with potential impact on enzyme expression or activity, patients carrying distinct genetic variants may have different capacity to repair genetic lesions. We conducted a systematic review to identify DNA repair SNPs that have been associated with altered response to radio or chemotherapy in the context of oral cancer and to characterize their impact on clinical outcome, therapeutic efficacy and safety.

**Materials and methods**

PRISMA guidelines were followed to answer the PICO question: “Do DNA repair SNPs influence the clinical outcome of anticancer treatment in patients with oral cancer?” Literature databases were searched, using the MeSH term-based expression (enriched with synonyms and hierarchically included terms): (Polymorphism, Single Nucleotide) AND ((DNA repair) OR (DNA repair enzymes)) AND ((mouth neoplasms) OR (Squamous Cell Carcinoma of Head and Neck)) AND ((drug therapy) OR (antineoplastic agents) OR (radiotherapy) OR (chemoradiotherapy)).

**Results**

Database search identified 142 studies, 7 being compliant with selection criteria. Significant associations with overall survival, disease-free survival or therapeutic efficacy were observed for 3 BER, 4 NER, 2 MMR and 1 HR SNPs. The variant genotype of *MLH1* rs1800734 was negatively associated with overall survival and disease-free survival in two independent studies.

**Conclusions**

*MLH1* rs1800734 may influence the clinical outcome of radio and/or chemotherapy in oral cancer patients. Further studies are needed to validate these results. Deepening such knowledge could help to individualize the treatment strategy according to the patient's genetic profile, thus improving clinical outcome in these patients.

#### P16 - Cytogenetic changes in exfoliated oral mucosa cells as effect biomarkers in dental diagnostic imaging: insights from a systematic review

##### Susana Alonso^2^, Maria José Correia^1,2^, Raquel Silva^1,2^, Luís Silva Santos^1,2^

###### ^1^ Universidade Católica Portuguesa, Center for Interdisciplinary Research in Health, Lisboa, Portugal; ^2^ Universidade Católica Portuguesa, Faculdade de Medicina Dentária, Viseu, Portugal

####### **Correspondence:** Luís Silva Santos (lsantos@ucp.pt)

*BMC Proceedings 2023,* **17(9):**P16

**Background**

Identification and validation of sensible and reliable biomarkers in biological samples, easily obtainable though non-invasive techniques, remains an active and attractive field in biomedical research. Saliva and exfoliated oral mucosa cells are among such samples and could become an important source of clinically relevant biomarkers for a vast spectre of applications. Diagnostic imaging techniques employing ionizing radiation (IR) are vastly used in modern dentistry but carry a cytotoxic and genotoxic risk. Through a systematic review of studies on dental patients submitted to radiological diagnostic techniques, we sought to verify whether cytogenetic changes in exfoliated oral mucosa cells can be used as sensible and reliable effect biomarkers of low dose IR exposure.

**Materials and methods**

The systematic review was conducted according to the PRISMA methodology and PICO criteria. Literature databases were searched, using an expression with the following MeSH terms: (Mouth mucosa) AND ((Chromosome Aberrations) OR (Cytogenetic Analysis) OR (Cytogenetics) OR (DNA damage) OR (Mutagenicity Tests)) AND ((Dental radiography) OR ((Dentistry) AND (Diagnostic imaging))).

**Results**

30 articles were selected for analysis, from the 246 records originally obtained. In 15 studies (50.0% of the total), cytological analysis was performed to determine the frequency of degenerative nuclear changes (pyknosis, karyolysis and karyorrhexis) in exfoliated oral mucosa cells, and diagnostic irradiation was associated with a statistically significant increase (*p* < 0.05) in at least one of the above-mentioned cytotoxicity markers (post-exposure versus pre-exposure comparisons). Changes were observed regardless of the radiographic imaging technique used, e.g., conventional X-ray techniques, panoramic radiography or cone-beam computer tomography (CBCT).

**Conclusions**

The frequency of degenerative nuclear changes (pyknosis, karyolysis and karyorrhexis) in exfoliated oral mucosa cells appears to be a sensitive and reliable effect biomarker of low dose IR exposure in the context of dental diagnostic imaging. These effect biomarkers could also prove useful to evaluate the cytotoxicity of other agents and materials commonly used in dentistry. However, further studies will need to be undertaken to ascertain that.

#### P17 - Excision repair SNPs may influence the extent of DNA damage from radioiodine therapy in lymphocytes from thyroid cancer patients

##### Luís Silva Santos^1,2,3^, Otávia Monteiro Gil^3,4^, Susana Nunes Silva^3^, Bruno Costa Gomes^3^, Teresa Ferreira^5^, Edward Limbert^5^, José Rueff^3^

###### ^1^ Universidade Católica Portuguesa, Center for Interdisciplinary Research in Health, Viseu, Portugal; ^2^ Universidade Católica Portuguesa, Faculdade de Medicina Dentária, Viseu, Portugal; ^3^ Centre for Toxicogenomics and Human Health (ToxOmics), Genetics, Oncology and Human Toxicology, NOVA Medical School|Faculdade de Ciências Médicas, Universidade Nova de Lisboa, Lisboa, Portugal; ^4^ Radiological Protection and Safety Unit – Technological and Nuclear Campus, Instituto Superior Técnico, Universidade de Lisboa, Bobadela-LRS, Portugal; ^5^ Dept. Nuclear Medicine, Instituto Português de Oncologia de Lisboa, Lisboa, Portugal

####### **Correspondence:** Luís Silva Santos (lsantos@ucp.pt)

*BMC Proceedings 2023,* **17(9):**P17

**Background**

Thyroid cancer (TC) is the most common endocrine malignancy, with rising incidence. Radioactive iodine (^131^I) is the standard therapy: ^131^I is captured by thyrocytes, releases ionizing radiation and inflicts DNA damage, inducing cell death. Prognosis is good. Because ^131^I may enter other cells, raising secondary malignancy risk, TC management guidelines now recommend cautious ^131^I use. Since DNA repair counteracts DNA damage, DNA repair SNPs may interfere with ^131^I-induced damage.

**Materials and methods**

We assessed micronuclei (MN) frequency in 26 TC patients undergoing ^131^I therapy – 15 patients exposed to 70 mCi, 11 to 100 mCi. MN levels were assessed before and after ^131^I exposure (1, 6 and 24 months for 70 mCi-treated patients; 1 and 3 months for 100 mCi-treated patients). Patients were genotyped for excision repair SNPs by real-time PCR, using TaqMan® Genotyping Assays (Applied Biosystems), or by PCR-RFLP. MN level variation from baseline was compared between genotypes, for each time point, in both dose groups. The study was approved by the Ethics Committees of Instituto Português de Oncologia Francisco Gentil (GIC/357) and Faculdade Ciências Médicas (CE-5/2008). Informed consent was obtained from all participants.

**Results**

*ERCC5* rs17655, *RAD23B* rs1805329, *XPC* rs2228000 and *XPC* rs2228001 variant allele carriers exhibited significant differences in MN frequency at one of the time points considered in at least one dose group. For *ERCC5* and *XPC* SNPs, significant differences in MN level variation from baseline were also observed in the 100 mCi group.

**Conclusions**

Excision repair SNPs may influence DNA damage, hence therapeutic outcome in ^131^I-treated patients. This could modify the risk of developing ^131^I-induced secondary malignancies and therapeutic efficacy. Further studies are needed to validate these results and to identify additional SNPs contributing to interindividual variability in response to ^131^I.

#### P18 - Identification and characterization of *Candida* spp. from denture stomatitis patients

##### Gonçalo Carvalho^1^, Juliana Lourenço^1^, Ana Abrantes^1,2^, Mónica Fernandes^1,2^, Maria José Correia^1,2^, Ana Sofia Duarte^1,2^, Raquel M Silva^1,2^

###### ^1^ Universidade Católica Portuguesa, Faculdade de Medicina Dentária, Viseu, Portugal; ^2^ Universidade Católica Portuguesa, Center for Interdisciplinary Research in Health, Viseu, Portugal

####### **Correspondence:** Raquel M Silva (rmsilva@ucp.pt)

*BMC Proceedings 2023,* **17(9):**P18

Fungi of the genus *Candida* are opportunistic pathogens that normally colonize mucosal tissues, from the oral cavity to the urogenital tract. The increase in *Candida* spp. infections is associated with their easy dissemination, ability to colonize surfaces including medical devices, and the development of cross-resistance to antifungal drugs.

The use of drugs with broad-spectrum antifungal activity, such as fluconazole, both for the treatment of patients and for prophylaxis, leads to the development of resistant strains. The difficulty in making an accurate diagnosis and a quick assessment of the susceptibility profile to antifungals may contribute to high morbidity and mortality rates in these infections.

In this work, we have identified *Candida* species isolated from patients with denture stomatitis, from the University Dental Clinic of the Universidade Católica Portuguesa in Viseu, Portugal. Phenotypic and molecular characterization of isolates was carried out, through the determination of their hemolytic activity, susceptibility profile to fluconazole and, in resistant isolates, the identification of polymorphisms related to the development of antifungal resistance.

These studies can further contribute to the prevention of more serious infections and to the design of alternative and more effective therapeutic options.

The project has been approved by the Health Ethics Committee from Universidade Católica Portuguesa (CES-UCP, nr. 113).

**Funding**

This work is financially supported by National Funds through FCT – Fundação para a Ciência e a Tecnologia, I.P., under the project UIDB/04279/2020 and by Programa Operacional Capital Humano e Fundo Social Europeu (FSE), under the projects SPRINT (CENTRO-01-0145-FEDER-181253) and OeHMP (CENTRO-01-0247-FEDER-072636).

Ana Abrantes and Mónica Fernandes thank the UCP for the Research grant, respectively, from SPRINT and OeHMP. Thanks are also due to FCT and UCP for the CEEC institutional funding of Raquel M Silva (CEECINST/00137/2018/CP1520/CT0012) and Ana Sofia Duarte (CEECINST/00137/2018/CP1520/CT0013).

#### P19 - Adaptation and implementation of the European matrix for teaching spiritual care to nursing students

##### Sara Sitefane^1^, Ana Afonso^1^, Isabel Rabiais^1^, Sílvia Caldeira^1^

###### ^1^ Institute of Health Sciences, Universidade Católica Portuguesa, Lisbon, Portugal

####### **Correspondence:** Sara Sitefane (s-ssitefane@ucp.pt)

*BMC Proceedings 2023,* **17(9):**P19

**Background**

The World Health Organization’s concept of health currently comprises eight dimensions: emotional, spiritual, intellectual, physical, environmental, financial, occupational, and social. Including the spiritual dimension [1] represents an essential milestone in recognizing its positive impact on health, well-being, and quality of life.

In this sense, nursing students' acquisition and development of spiritual care skills are required, particularly in undergraduate nursing degrees. Also, the evidence demonstrates the positive relationship between spiritual education and spiritual competencies, emphasizing the need for spiritual education as an integral and regular part of the undergraduate nursing *curriculum*. Regardless of this evidence, the educational strategies for improving and developing undergraduate nursing students’ skills and competencies are scarce and should be urgently considered as nurses and midwives still report feeling unprepared for providing spiritual care.

Recently, the EPICC project (Nurses' and Midwives' Competence in Providing Spiritual Care through Innovative Education and Compassionate Care)[2] has been implemented as a turning point in nursing education for spiritual care and spirituality, through a systematic, consensual, and effective response, by involving multiple partners and experts from different European countries. Portugal has been a participant in that Erasmus-funded project.

**Materials and methods**

This Ph.D. project concerns the translation, adaptation, and implementation of the EPICC matrix. First, a translation and cultural adaptation process will be conducted according to the core project guidelines [3]. Then the matrix for education and assessing spiritual care competencies will be implemented in a pilot study in a Portuguese nursing school involving undergraduate nursing students.

**Results**

The core project guidelines represent V stage of the cross-cultural adaptation process. The preliminary results point to the beginning of stage III with the back translation of the synthesized written version of the EPICC Spiritual Care Education Standard and EPICC Spiritual Care Competency Self-assessment tool. Stage I (initial translation with written reports of each – T1 and T2) and Stage II (synthesis of the translations to version T12) of the translation and cultural adaptation of the EPICC matrix are already concluded.

**Conclusions**

This innovative project could help improve Portuguese schools' nursing curricula from an evidence-based perspective.

**References**

1. World Health Organization. The Spiritual Dimension: Resolution of the Executive Bord of the WHO. 1984.

2. McSherry W, Ross L, Attard J, van Leeuwen R, Giske T, Kleiven T, et al. Preparing undergraduate nurses and midwives for spiritual care: Some developments in European education over the last decade. Journal for the Study of Spirituality. 2020 Jan 2;10(1):55–71.

3. Beaton DE, Bombardier C, Guillemin F, Ferraz MB. Guidelines for the Process of Cross-Cultural Adaptation of Self-Report Measures. Spine (Phila Pa 1976). 2000;25(24):3186–91.

### Session 2 - Clinical Care

#### P20 - Surgical Site Infections in Colorectal Surgery and generic prevention bundles

##### Tiago Cunha^1, 2^, João Maciel^1^, Susana Miguel^1, 4^, Carlos Zagalo^1, 3^, Paulo Alves^4^

###### ^1^ Instituto Português de Oncologia de Lisboa Francisco Gentil, E.P.E.; ^2^ Universidade Católica Portuguesa, Instituto de Ciências da Saúde, Portugal; ^3^ Instituto Universitário Egas Moniz, Centro de Investigação Interdisciplinar Egas Moniz, Portugal; ^4^ Universidade Católica Portuguesa, Center for Interdisciplinary Research in Health, Lisboa, Portugal

####### **Correspondence:** Paulo Alves (pjalves@ucp.pt)

*BMC Proceedings 2023,* **17(9):**P20

**Background**

Surgical Site Infections are amongst the most frequent complications in colorectal surgery. Colorectal Surgical site infection rates in Europe have seen a modest decrease when compared with other types of surgical procedures.

**Discussion**

The European surveillance system has studied surgical site infections in the last two decades being colorectal surgery the highest among them.

Several surgical site infection prevention bundles have been introduced since, but few of them are tailored to colorectal surgery.

**Methods**

A retrospective study was undertaken from colorectal surgeries performed between 2011 and 2020. An analysis of annual surgical site infection rates, as well as the compliance to the Portuguese surgical site infection prevention bundle between 2019 and 2020.

**Results**

2345 colorectal surgeries were studied in accordance with HELICS/HAI-Net SSI protocols. Surgical site infection rates varied between 26.35% (2013) and 34.10% (2019), with an average of 30.06%.

Regarding the compliance rate of the prevention bundle, results were underwhelming, but it was noted that glycemic control (84.9%) and hair removal avoidance (74.5%) were the individual interventions with better observance.

**Conclusion**

The development of prevention bundles tailored to colorectal surgery could prove to be an adequate tool for a sustained reduction of surgical site infection rates.

#### P21 - Surgical Site Infections in Colorectal Surgery: a Portuguese prespective

##### Tiago Cunha^1, 2^, João Maciel^1^, Susana Miguel^1, 4^, Carlos Zagalo^1, 3^, Paulo Alves^4^

###### ^1^ Instituto Português de Oncologia de Lisboa Francisco Gentil, E.P.E.; ^2^ Universidade Católica Portuguesa, Instituto de Ciências da Saúde, Portugal; ^3^ Instituto Universitário Egas Moniz, Centro de Investigação Interdisciplinar Egas Moniz, Portugal; ^4^ Universidade Católica Portuguesa, Center for Interdisciplinary Research in Health, Lisboa, Portugal

####### **Correspondence:** Paulo Alves (pjalves@ucp.pt)

*BMC Proceedings 2023,* **17(9):**P21

**Background**

Surgical Site Infections are amongst the most frequent complications in colorectal surgery and are associated with increased healthcare and socioeconomical costs.

**Methods**

A retrospective study was undertaken from colorectal surgeries performed between 2011 and 2020. An analysis of annual surgical site infection rates, as well of types of infection was performed. Data of compliance to the Portuguese surgical site infection prevention bundle was collected and analyzed from 2019 and 2020.

**Results**

2345 colorectal surgeries were studied in accordance with HELICS/HAI-Net SSI protocols. Surgical site infection rates varied between 26.35% (2013) and 34.10% (2019), with an average of 30.06%. Overall the most prevalent microorganisms were *Escherichia coli* (23.79%) e *Enterococcus faecalis* (21.37%). By infection type the most frequent micoorganisms were *Enterococcus faecalis* (27.47%) in superficial, *Escherichia coli* (25.92%) in deep incisional and *Escherichia coli* (26.21%) in organ/space surgical site infections.

Regarding the compliance rate to Portuguese surgical site infection prevention bundle, results were underwhelming, but it was noted that glycemic control (84.9%) and hair removal avoidance (74.5%) were the individual interventions with better observance.

**Conclusion**

Surgical site infection rates in colorectal surgery are still high despite the use of prevention bundles. Targeted bundles may be the answer.

#### P22 - RadWounds | Dosimetric impact of the wound dressing material used in radiotherapy

##### Marisa Matos^1,2,3^, Joana Lencart^1,3^, Pedro Dias^1,3^, João Santos^1,3^, Jorge Freitas^1,3^, Paulo Alves^2,4,5^

###### ^1^ Instituto Português de Oncologia, Porto, Portugal; ^2^ Associação Portuguesa Tratamento de Feridas (APTFeridas); ^3^ Radiobiology and Radiation Protection, Instituto Português de Oncologia, Porto, Portugal; ^4^ Universidade Católica Portuguesa, Center for Interdisciplinary Research in Health, Portugal; ^5^ Universidade Católica Portuguesa, Instituto Ciências da Saúde, Escola Enfermagem (Porto), Portugal

####### **Correspondence:** Paulo Alves (pjalves@ucp.pt)

*BMC Proceedings 2023,* **17(9):**P22

**Background**

External radiotherapy (ER) is one of the oncological therapeutic pillars, being used by approximately with cancer patients. The ionizing radiation used in ER damages the cellular components, particularly the basal layer of the skin, which leads to an imbalance between normal cell multiplication in this layer and cell damage to the skin surface.

**Objective**

The presence of any material on the patient's skin during irradiation can increase the dose on the skin (bolus effect). The aim of this study was to evaluate in which cases the increase dose caused by using dressings during the treatment is negligible, acceptable, or even favorable to treatment.

**Method**

A total of 21 samples of 14 different materials, including foams, alginates, felling fibers, silicones and emollients evaluated alone or in combinations. Dosimetry was evaluated in the original dressing, as well as saturated with saline (if applicable), to simulate saturation with exudate. Radiosensitive films were used to evaluate the increase dose to 1mm depth, caused by the introduction of those products on the surface of an equivalent soft tissue material, during irradiation with a beam of 6MV photons.

**Results / Discussion**

From the 21 samples, the increase dose at 1mm depth was: less than 20% in 11 cases; in 5 cases between 21% and 40%, and in the last 5 cases between 41% and 80%. We The immobilization masks in ER was the threshold and the results were compared with the increased dose resulting from the use of the dressings, which can differ between 30% and 90%. It was possible to verify that the materials that absorbed more radiation were those saturated with saline solution, and the addition of several materials can significantly influence the dose distribution and the amount of exudate absorbed.

**Conclusion**

Depending on disease and fractionation of ER, may be considered beneficial the use of some evaluated dressings during irradiation due to patient comfort, treatment results and toxicity.

#### P23 - Behavior of two different torque wrenches used in oral implantology – *in vitro study*

##### Filipe Araújo^1,2^ , Jorge Macário^1^**,** Helena Salgado^1,2^, Tiago Marques^1,2^, André Correia^1,2^

###### ^1^ Universidade Católica Portuguesa, Faculty of Dental Medicine (FMD), Viseu, Portugal; ^2^ Universidade Católica Portuguesa, Center for Interdisciplinary Research in Health (CIIS), Viseu, Portugal

####### **Correspondence:** Filipe Araújo (faraujo@ucp.pt)

*BMC Proceedings 2023,* **17(9):**P23

**Background**

Torque limiting devices (torque wrenches) are commonly used in implant dentistry. Implant manufacturers recommend these devices to deliver the target torque value to the screw because inadequate torque delivery is sometimes attributed to screw loosening.

There are several types of manual torque wrenches, the most common are the “toggle-style” and the “spring-style” systems.

**Objectives:** To study the behavior of two types of mechanical torque limiting devices used in current oral rehabilitation.

**Materials and Methods**

A laboratory study was carried out to test the mechanical behavior of two different torque limiting devices, spring-style, and toggle-style. For each torque wrench it was prepared 3 working tables with an implant analog, straight standard abutment, and respective screw. For each system, 30 tightening/loosening cycles were carried out for 3 pre-selected values: 10, 20, 35N.Cm. The values were recorded using a dynamometric measurement cell.

**Results**

Both studied devices presented lower torque values when compared to the pre-selected torque, showing significantly different results. Toggle-style and spring-style systems also had different results, being Toggle-Style inferior to spring-style.

**Conclusion**

Within the limitations of this research, it can be concluded that the analyzed torque wrenches systems presented inferior values to those pre-selected. Precaution must be taken by the Dentist when applying the recommend manufacturer torque values to prosthetic/implant screws to avoid mechanical complications.

#### P24 - Influence of non-carious lesions in the surgical treatment of gingival recession: a new data collection method

##### Filipe Araújo^1,2^, Helena Salgado^1,2^, André Correia^1,2^, Javier Montero^3^, Norberto Quispe^3^, Tiago Marques^1**,**2^

###### ^1^ Universidade Católica Portuguesa, Faculty of Dental Medicine (FMD), Viseu, Portugal; ^2^ Universidade Católica Portuguesa, Center for Interdisciplinary Research in Health (CIIS), Viseu, Portugal; ^3^ Surgery Department, Universidad de Salamanca, Salamanca, Spain

####### **Correspondence:** Filipe Araújo (faraujo@ucp.pt)

*BMC Proceedings 2023,* **17(9):**P24

**Introduction**

Non-carious cervical lesions are a pathological condition that can be defined as the wear of the solid tooth substance at the level of the gingival one-third. From a topographic point of view, a non-carious lesion can involve only the crown, or only the root surface, or it can cover both. When the non-carious lesions involve the root, a gingival recession is often observed. Restorative with or without surgical coverage are accepted procedures for non-carious cervical lesions treatment.

**Objective**

develop a new digital evaluation protocol to objectively quantify the volumetric changes of root coverage regarding the treatment of non-carious cervical lesions.

**Materials and Methods**

Data (.stl files) from a sample of patients with Cairo recession type 1 or Cairo recession type 2 were analyzed. This data reported to the 3D volumes of the surgical area in time point 0 and after 3 and 6 months was analyzed using specific software. Measurement protocol with specific informatic tools was developed by selecting the points/areas of interest and testing it in several cases.

**Results**

The application of this measurement protocol in the data of several clinical cases was possible. The healing dynamics could be measured by calculating volume differences between time points. This protocol was successfully tested in a clinical case series. Significant differences were found on volume, area of coverage, average thickness on both evaluated time points.

**Conclusions**

The digital protocol presented proved to be a non-invasive technique for accurate measurements of clinical outcomes.

**Ethical Issues**

The study protocol was approved by Ethtics Commission for Health of the University (Comissão de Ética para a Saúde da Universidade Católica Portuguesa, Report number 25, 4th of June 2020). Informed consent was obtained from all participants and all methods were performed in accordance with the Declaration of Helsinki principles for medical research involving human subjects and following the requirements established by Portuguese Law n° 21/2014 for clinical research.

The aim of the study is mainly focused on a new informatics methodology to analyze biological findings and outcomes.

#### P25 - Mechanical behavior of three dynamic solutions used in prosthetic rehabilitation - a pilot study

##### Filipe Araújo^1,2^, Catarina Saramago^1^, Hernâni Lopes^3^ Helena Salgado^1,2^, Tiago Marques^1,2^, André Correia^1,2^

###### ^1^ Universidade Católica Portuguesa, Faculty of Dental Medicine (FMD), Viseu, Portugal; ^2^ Universidade Católica Portuguesa, Center for Interdisciplinary Research in Health (CIIS), Viseu, Portugal; ^3^ Instituto Superior de Engenharia do Porto, Portugal

####### **Correspondence:** Filipe Araújo (faraujo@ucp.pt)

*BMC Proceedings 2023,* **17(9):**P25

**Background**

Oral rehabilitation can be performed using dental implants. The existence of anatomical limitations can prevent the implant from being placed in an ideal prosthetic position, making rehabilitation a challenge. Due to these limitations, several problems can arise, for example, the aesthetic compromise associated with the appearance of the screw access channel in visible areas. To overcome this situation, dynamic solutions with “angled screw channels” were developed, which allow access to the screw channel using a multiangular screwdriver.

**Objectives:** To study and validate the mechanical behavior of 3 dynamic solutions.

**Materials and Methods**

Mechanical behavior of 3 dynamic solutions (BHS30®, UBH30® 4 grooves, UBH30® 6 grooves) was evaluated. First test, the samples of each system were tightened with the recommended torque of 30 N.cm, at 0° and 30°, and 10 and 30 tightening cycles were performed. The screw and the screwdriver were analyzed using laboratory loupes. Second test, the systems were submitted to a progressive torque until a fracture and/or deformation of one of the components occurred, to determine the maximum torque value that they support.

**Results**

No significant differences were observed between dynamic *vs* standard systems. Comparing the different dynamic systems, it was found that the UBH30® 4 grooves is the one with the highest average torque values, being also the only system that doesn’t show variations in torque values between 0° and 30°. BHS30® is the system that supports higher maximum torque values.

**Conclusions**

Dynamic solutions can be considered a viable option in Implantology. However, there must be caution with the system used, since there are differences in the maximum torque values supported.

#### P26 - The sense and meaning of comfort in the lived experience of the survivors of allogeneic hematopoietic stem cells transplantation

##### Lúcia Bacalhau^1^, Patrícia Pontífice Sousa^2^

###### ^1^ RN, MsC, PHD Student, Institute of Health Sciences, Center for Interdisciplinary Research in Health, Universidade Cátolica Portuguesa, Lisbon, Portugal; ^2^ RN, MSC, PhD, Associated Professor, Institute of Health Sciences, Center for Interdisciplinary Research in Health, Universidade Católica Portuguesa, Lisbon, Portugal

####### **Correspondence:** Lúcia Bacalhau (luciabacalhau@gmail.com)

*BMC Proceedings 2023,* **17(9):**P26

**Background**

Allogeneic Stem Cells Transplantation (ASCT), and consequently, the chronicity associated with this life event, have a growing prevalence and a significant impact on the life and daily life of each person who experiences it. The increase in the number of survivors of the transplant process leads us to reflect on the challenges that these people face in this prolonged process. The aim of this study was understand the meaning and sense of comfort for the survivor of ASCT.

**Materials and Methods**

To understand the lived experience of comfort, we used a qualitative approach using van Manen's phenomenology of practice. We uncovered the phenomenon through phenomenological interviews and illustrative episodes that reflected the lived experience of twenty survivors. In the process of analysis, we followed the "stages" such as epoché, reduction, and vocative. The MAXQDA support was facilitative in organizing the data and in the analysis process.

**Results**

The results revealed the complexity of the phenomenon in relation to the understanding of how the "Being a survivor" person experiences comfort in the daily life of the ASCT process. From the analysis, the following aspects emerged as meaning and sense of comfort: "to give time to life", "to live with well-being", "to give meaning to the days" and "the opportunity to achieve healing". We found comfort as something desired, ambitioned to promote well-being and sense of happiness for survivors.

**Conclusion**

Regarding the comforting sphere, this knowledge allows personalizing and adapting nursing care to the survivor, drawing attention to the need for intervention in this domain of living and contributing to achieve full comfort and improve the quality of nursing care.

**Keywords**

Comfort, Survivor, Stem Cells Transplant, Lived Experience, Nursing, Phenomenology

The study was approved by the Ethics Committee for Health of the Instituto Português de Oncologia de Lisboa, Francisco Gentil, EPE under reference UIC 1314.

All participants voluntarily consented to collaborate in this study and agree to its publication, they signed the informed consent form after clarification of the study procedures.

#### P27 - Translation and Cross-Cultural Adaptation for Portuguese of the Nurses’ Foot Care Knowledge Test

##### Rafael Bernardes^1,2^, Sílvia Caldeira^2^, Minna Stolt^3^, Arménio Cruz^1^

###### ^1^ The Health Sciences Research Unit: Nursing (UICISA:E), Nursing School of Coimbra (ESEnfC), Portugal; ^2^ Universidade Católica Portuguesa, Center for Interdisciplinary Research in Health, Lisboa, Portugal; ^3^ Department of Nursing Science, University of Turku, Finland

####### **Correspondence:** Rafael Bernardes

*BMC Proceedings 2023,* **17(9):**P27

**Background**

Nurses are frequently exposed to prolonged standing environments while at work, which is a significant risk factor for the development of musculoskeletal disorders, particularly of the foot and ankle. Such a fact can decrease quality of life and work satisfaction and motivate professional absenteeism. Few studies about this topic mention that nurses acknowledge foot health as an essential occupational factor belatedly, specifically when signs and symptoms are already evidenced and, in some cases, hindering.

To propel adequate preventive interventions and raise awareness among nurses for the importance of adopting efficient self-care actions during their life, it is important to assess their knowledge. Moreover,

The Nurses’ Foot Care Knowledge Test (NFKT®) was initially developed in Finland by Minna Stolt and is effective in assessing nurses’ knowledge of foot care.

Therefore, this study aims to translate and culturally adapt to Portuguese, the NFKT.

**Materials and Methods**

The methodological study followed a five-stage process proposed by Beaton and colleagues: translation, synthesis, back translation, expert committee review, and pretesting. A total of seven experts reviewed all translations and proposed changes in a Delphi process, where consensus was defined when at least 75% of the participants agreed to each item. The pretesting was performed with 30 participants to test comprehension and clarity of items. Ethical Committee Approval nr. P799_07_2021 and Clinical Trials.gov registration nr. NCT05197166.

**Results**

A total of two rounds were necessary to reach the final version. Only two items required rewording. The final version was named *Teste de Conhecimento dos Enfermeiros sobre Cuidados ao Pé (TCE-CP)* and was conceptually equivalent to the original scale. The pre-test didn’t lead to any item revision.

**Conclusions**

The TCE-CP version of the NFKT questionnaire showed equivalency with the original instrument, having the same potential to assess nurses’ knowledge regarding their foot health effectively.

#### P28 - Usability testing of the Implant Disease Risk Assessment IDRA, a tool for preventing peri-implant disease: protocol design

##### Rita Bornes^1,2^, Javier Montero^3^, André Correia^1,2^, Nuno Rosa^1,2^, Ana Ferreira^4^

###### ^1^ Universidade Católica Portuguesa, Faculty of Dental Medicine (FMD), Viseu, Portugal; ^2^ Universidade Católica Portuguesa, Center for Interdisciplinary Research in Health (CIIS), Viseu, Portugal; ^3^ University of Salamanca Department of Surgery, Faculty of Medicine, University of Salamanca, Salamanca, Spain; ^4^ CINTESIS@RISE, FMUP-MEDCIDS, Porto, Portugal

####### **Correspondence:** Rita Bornes (rbornes@ucp.pt)

*BMC Proceedings 2023,* **17(9):**P28

**Background**

A risk assessment tool named Implant Disease Risk Assessment (IDRA) was developed to help professionals predict the development of peri-implantitis. Eight parameters were organized into an octagon-shaped functional diagram capable of providing an individual risk profile and determining the need for risk reduction measures. To design a protocol to test the usability of IDRA-TOOL within a population of dentists dedicated to the field of Implantology.

**Materials and Methods**

In order to identify tasks or features of the tool that are difficult to perform, assess the clarity of the language used, the average time to complete the tasks, identify design issues and factors or other requirements that may have not been considered and that could improve the efficiency of the tool, a convenience sample of 8 dentists dedicated to the field of Implantology and interested in participating in the IDRA-TOOL usability tests is used. Literature research was done to identify methods that could help in the development of this usability tests.

**Results**

This study made it possible to define the usability protocol for the IDRA-TOOL, which should follow the following steps: i) Each participant accesses a link at the QUALTRICS platform where they find the pre-defined clinical use cases with specific data, who anonymously carry out the same steps and enter or search for the same information while performing the same tasks. Each task is observed by the responsible researcher. ii) Participants are asked to use the *Think Aloud* approach. iii) Afterwards, the survey System Usability Scale should be completed. iv) A face-to-face interview with audio recording will be then conducted by the responsible researcher, asking participants about their experience and what suggestions can be considered to improve the tool.

**Conclusions**

The usability protocol that was developed can help in future research in the optimization of IDRA-TOOL to be easily applied in the clinical practice.

#### P29 - Standardized language systems used by nurses in palliative care in Portugal

##### Joana Bragança^1,2,3^, Pedro Tavares^3^, Carlos Rodrigues^3^, Lurdes Martins^1,2^, Silvia Caldeira^1,2^

###### ^1^ Universidade Católica Portuguesa, Center for Interdisciplinary Research in Health, Lisboa, Portugal; ^2^ Universidade Católica Portuguesa, Instituto de Ciências da Saúde, Lisboa, Portugal; ^3^ Hospital da Luz, Lisboa, Portugal

####### **Correspondence:** Joana Bragança (s-jfbraganca@ucp.pt)

*BMC Proceedings 2023,* **17(9):**P29

Given the increasing complexity of palliative care patients and care delivery, the adoption of tools that support this reality is essential. Standardized terminologies play a fundamental role in guiding the different stages of the nursing process, leading to an improvement in the quality of care provided.

To identify the standardized nursing languages, nursing record methodologies available and used by nurses in palliative care (PC) 108 PC nursing teams were contacted and 56 have replied; 94.6% reported using electronic and in 89.3% nursing data are stored in a standardized electronic format. The most prevalent standardized language system for nursing is ICNP (94.6%). In 39.3% of the teams, nursing data cannot be measurable or recoverable, and when it is possible, 41.2% of teams have already used that data in research development. The more known standardized nursing language systems are ICNP, NANDA-I, NIC and NOC.

In PC, avoidable suffering is perpetuated by the lack of knowledge, requiring the updating of evidence-based tools in this area. The knowledge and development of standardized languages ​​will contribute to the improvement of the quality of care provided, as well as support the production of scientific evidence related to the nursing practice in PC.

#### P30 - Is anxiety related to higher vulnerability to a body illusion? Results from the classical rubber hand illusion paradigm

##### Duarte Santos^1^, Mariana Agostinho^2^, Rita Canaipa^2^

###### ^1^ Institute of Health Sciences, Universidade Católica Portuguesa, Lisbon, Portugal; ^2^ CIIS, Center for Interdisciplinary Health Research, Institute of Health Sciences, Universidade Católica Portuguesa, Lisbon, Portugal

####### **Correspondence:** Rita Canaipa

*BMC Proceedings 2023,* **17(9):**P30

**Background**

The Rubber Hand Illusion is a classical body illusion that induces in the individual the feeling that a rubber hand positioned in the expected place of the real hand is its own. It is believed that the illusion is a consequence of the integration of ascending signals of three main sensory modalities: proprioceptive, visual, and tactile. As such, it has been proposed that individuals with a lower ability to detect and interpret body signals may be more vulnerable to being affected by the illusion. The current study aimed to investigate if individual differences in anxiety, known to affect the perception of body signals were associated with the vulnerability to sense this body illusion.

**Materials and methods**

Healthy volunteers underwent the two classic conditions of the RHI, synchronous and asynchronous. The feeling of ownership and the proprioceptive drift in each condition were the main outcomes. Anxiety was measured using the State-Trait Anxiety Inventory (STAI). Spearman’s correlations were used to assess associations between the illusion outcomes and the state and trait anxiety. The study was approved by the Ethical Committee of the Universidade Católica Portuguesa. The individuals who agreed to participate provided written informed consent.

**Results**

Forty-five healthy volunteers have been enrolled in the study. Trait anxiety was correlated with the feeling of ownership in the asynchronous condition (Spearman’s r = 0.325, p = 0.029), and the state anxiety correlated with all proprioceptive measures, such as proprioceptive drift in synchronous (Spearman’s r = 0.299, p = 0.049) and asynchronous condition (Spearman’s r = 0.310, p = 0.038), and with the feeling of ownership in the control items (Spearman’s r = 0.335, p = 0.025).

**Conclusions**

These results suggest that the sensitivity to the RHI is higher in individuals with higher anxiety. As measured by the changes in proprioception, the vulnerability to body illusions might be increased in individuals with high-state anxiety, thus suggesting difficulties in maintaining a stable body schema and interpreting body signals that may have clinical consequences.

#### P31 - The relationship between the ability to detect ascending sensory signals, and the emotions and beliefs in expectations of pain reduction - an experimental conditioning task

##### Mariana Agostinho^2^, Duarte Santos^1^, Adriana Gutierrez^1^, Miguel Réfega^1^, Rita Canaipa^2^

###### ^1^ Institute of Health Sciences, Universidade Católica Portuguesa, Lisbon, Portugal; ^2^ CIIS, Centre for Interdisciplinary Health Research, Institute of Health Sciences, Universidade Católica Portuguesa, Lisbon, Portugal

####### **Correspondence:** Rita Canaipa

*BMC Proceedings 2023,* **17(9):**P31

**Background**

According to the Bayesian Brain Hypothesis, pain results from the interaction of the certainty that individuals have in the priors, encompassing emotions, expectations, and conditioning responses from previous experiences, as well as the confidence of the ascending sensory information. In this study, we aimed to assess factors contributing to these certainties and possible associations between these two components.

**Materials and methods**

Healthy volunteers underwent an experimental placebo paradigm (EPP) to assess their confidence in the priors. Expectations of benefit were induced by pairing red and green cues to high and low stimuli intensity during a conditioning phase, followed by a second phase where the stimuli intensities were equal for both colors/cues. Expectations were measured before, during, and at the end. In addition, the participants performed the Focused Analgesia Selection Test (FAST), which measures the within-subject variability in pain reports. Questionnaires assessing emotions (The Hospital Anxiety and Depression Scale, HADS) and beliefs (Pain Catastrophization Scale, PCS) were also used to assess individual differences. Study approval was obtained from the Ethical Committee of the Universidade Católica Portuguesa. The individuals who agreed to participate provided written informed consent.

**Results**

Twenty-six participants completed the study. No correlations were found between the FAST outcomes, the conditioning, the test phase, or expectations at the EPP. Correlations were found between the HADS anxiety and the placebo (Spearman’s r=-.606, p<.001). Expectations before the conditioning (Spearman’s r= .402 e p= .042) and after (Spearman’s r= .443, p= .023) the placebo were positively correlated with the PCS.

**Conclusions**

The ability to perceive ascending sensory signals, as measured by the within-subject variability in pain reports was not related to the intensity of conditioning or placebo response in the pain task in healthy individuals. Aligned with previous studies, we found that individuals with higher anxiety levels were less vulnerable to the placebo effect, and catastrophization may have a critical role in expectations of pain reduction during the conditioning by considering the treatment to be more effective.

#### P32 - Endodontic treatment of a geminated canine - case report

##### Miguel Cardoso^1,2^, Mariana Duarte^2^, Giovana Siqueira^2^, Diana SottoMayor^2^, Rita Noites^1,2^

###### ^1^ Universidade Católica Portuguesa, Center for Interdisciplinary Research in Health, Viseu, Portugal; ^2^ Universidade Católica Portuguesa, Faculdade de Medicina Dentária, Viseu, Portugal

####### **Correspondence:** Miguel Cardoso (mabcardoso@ucp.pt)

*BMC Proceedings 2023,* **17(9):**P32

**Background**

Dental gemination is a designation used to identify a disturbance occurred during odontogenesis that causes changes in the shape of the tooth. It is recognized as a failed attempt by a single tooth germ to divide by invagination, resulting in a single wide tooth with a bifid crown. It is a rare condition that occurs with greater prevalence in the primary dentition and affects mostly incisor teeth. Its etiology is not clearly understood, although there is evidence that it may be related to genetic factors, trauma and with some syndromes. Despite being generally asymptomatic, it can manifest clinical alterations like malocclusion, impaction of adjacent teeth and even greater susceptibility to caries and periodontal destruction.

**Case report**

A 22-year-old female patient with Fahr's Syndrome was referred to the endodontic appointment at the Clinica Dentária Universitária of Universidade Católica Portuguesa in Viseu, presenting tooth 23 with symptoms compatible with a state of irreversible pulpitis. Periapical radiographs and CBCT confirmed the diagnosis of geminated tooth with irreversible pulpitis. Endodontic treatment was performed using an operative microscope. The preparation was performed with ProTaper^TM^ Gold F5 using 2.5% sodium hypochlorite irrigation. In the obturation, two techniques were used, continuous wave compaction in the apical third and gutta-percha injection with vertical compaction in the remain canal. The crown was restored with a direct composite. After six months, the tooth maintained the function and there was no symptomatology. The radiograph was normal, predicting a good evolution. Informed consent was obtained for publication.

**Conclusion**

Dental gemination is an anomaly diagnosed through clinical and radiological criteria. A careful clinical and radiographic examination, as well as the use of an operative microscope, increases the probability of success and improves the prognosis of endodontic treatment. The recognition and adequate treatment of this anomaly allowed to restore the patient's function and aesthetics.

#### P33 - FMD-Caries Risk Assessment Index - A new caries risk assessment index including salivary and microbiological factors

##### Pedro Lopes^1^, André Fonseca^2^, Vanessa Ribeiro^2^, Ana Mendes^1^, Ana Gomes^1^, Marla Pinto^1^, Rute Rio^1^, Maria Correia^1^

###### ^1^ Universidade Católica Portuguesa, Faculty of Dental Medicine, Center for Interdisciplinary Research in Health, Viseu, 3504-505, Portugal; ^2^ Universidade Católica Portuguesa, Faculty of Dental Medicine, Viseu, 3504-505, Portugal.

####### **Correspondence:** Maria Correia

*BMC Proceedings 2023,* **17(9):**P33

**Background**

Caries risk assessment, although not routinely performed by most dentists, is an essential tool to personalize caries treatment plans [1, 2]. However, the existing indexes are not easily applied in the dental appointment and most dentists doubt their predictive efficiency, relying mostly on clinical experience [3]. Improvement of the current indexes is desirable to make caries risk assessment a more objective and effective task. Microbiological and salivary factors can provide essential information [4,5] and should be included in the risk assessment. This work aims at developing a caries risk assessment index based on the CAMBRA [6] index modified by adding and changing the factors analyzed to improve the predictability and assessment of risk caries in individuals.

**Materials and methods**

The CAMBRA caries risk index was modified and was subsequently applied in a cross-sectional observational study involving 80 patients who attended the Dental Clinic of the Universidade Católica Portuguesa - Viseu. Each patient who met the necessary requirements to participate in this study was observed by the researchers who collected patient data through a questionnaire performed an intraoral evaluation and collected of saliva and biofilm samples.

**Results**

In the 80 patients evaluated, we classified 56.25% (n=45), moderate risk 27.50% (n=22), high risk 13.75% (n=11) and severe risk 2.50% (n=2). The index was easy to apply, took about 5 minutes for the whole procedure and is feasible to apply chairside. Association between the caries risk and total bacterial load in saliva was not statistically significant.

**Conclusions**

The feasibility of applying the proposed index as a chairside method was verified, as well as the changes necessary to apply the index more generally in the University Dental Clinic, in all the first appointments. In this way, patient follow-up becomes more accurate and more personalized and future dentists are trained in this type of assessment that they will be able to develop in their postgraduate clinical practice.

Future work involves the recall of the same patients after 6-8 months to verify is the caries risk status changed.

**References**

1. L SK, Ünlü N. Effectiveness of Different Preventive Programs in Cariogram Parameters of Young Adults at High Caries Risk. 2017;2017

2. Wardani R, Zubaedah C, Setiawan AS. Occlusal caries risk assessment using cariogram analysis in student aged 11-12 years. (1):13–20

3. Featherstone JDB, Crystal YO, Alston P, Chaffee BW, Doméjean S, Rechmann P, et al. A Comparison of Four Caries Risk Assessment Methods. 2021;2(April):1–13

4. Chen X, Daliri EB, Tyagi A. Cariogenic Biofilm: Pathology-Related Phenotypes and Targeted Therapy. 2021

5. Strużycka I. The Oral Microbiome in Dental Caries. 2014;63(2):127–35

6. Gannam C V, Chin KL, Gandhi RP. Caries risk assessment. 2018

#### P34 - Orthodontics after dental trauma – when and how?

##### Tatiana Serrão^1^, Susana Silva^1,2^, Patrícia Correia^1,2^

###### ^1^ Faculty of Dental Medicine, Universidade Católica Portuguesa, Viseu, Portugal; ^2^ Centro de Investigação Interdisciplinar em Saúde, Universidade Católica Portuguesa, Viseu, Portugal

####### **Correspondence:** Patrícia Correia (pcorreia@ucp.pt)

*BMC Proceedings 2023,* **17(9):**P34

**Background**

Dento-alveolar injuries are very common in children and adolescents, ranging from mild injuries, such as concussion, to tooth loss, as an avulsion. Trauma management depends on the type of trauma sustained. Future dental treatment, such as orthodontic treatment, may be compromised due to a dental trauma history. The aim of this review was to determine the assessment criteria, when planning and performing orthodontic treatment on traumatized teeth, in order to prevent future dental complications.

**Materials and Methods**

A systematic review was based on PRISMA guidelines and a literature search was conducted in PubMed/Medline®, Web of Science, Embase and ProQuest Dissertation, according to a predetermined PICO question. Keywords were defined and combined with Boolean operators. Filters were applied, as well as inclusion and exclusion criteria. This review was registered in the international database PROSPERO.

**Results**

Initially, 1065 articles were retrieved, after applying the filters and removing duplicates, 973 articles were obtained. After reading the title and abstract and applying the eligibility criteria, 11 studies were selected. At the end, after screening the full text, 5 studies were included in this review. The results were heterogeneous due to the range of trauma types and orthodontic treatment undertaken. Globally, regular pulp vitality checks and reduced orthodontic forces were advised.

**Conclusions**

Prior orthodontic treatment, a thorough evaluation is required on the trauma-involved teeth. Careful follow-up once treatment has started is key for a favourable long-term prognosis.

#### P35 - Contemporary Scientific Evidence on Silver Diamine Fluoride

##### Kelyane Moraes^1^, Nélio Veiga^1,2^, Anna Carolina Moura^1,2^, Patrícia Correia^1,2^

###### ^1^ Faculty of Dental Medicine, Universidade Católica Portuguesa, Viseu, Portugal; ^2^ Centro de Investigação Interdisciplinar em Saúde, Universidade Católica Portuguesa, Viseu, Portugal

####### **Correspondence:** Patrícia Correia (pcorreia@ucp.pt)

*BMC Proceedings 2023,* **17(9):**P35

**Background**

Dental caries is still the most common oral disease in the world. Silver diamine fluoride (SDF) has emerged as an important armamentarium on the control of dental caries, due to its safety, efficiency and feasibility. Clinical trials have provided some evidence supporting the use of SDF to stop dentin caries in preschool children. The aim of this study was to assess SDF usage in the management of dental caries in children.

**Materials and Methods**

A systematic review was developed following the PRISMA guidelines using a predetermined research question. Search was performed in PubMed/MEDLINE®, Web of Science, Embase and Scopus databases; without time limits, in English and Portuguese, using specific keywords and Boolean operators. This review was registered in the international database PROSPERO.

**Results**

The literature search resulted in 1611 studies which, after applying the selection criteria and removing duplicates led to 13 considered an important agent in caries arrest in school-age children studies, that were read in full and included in this review. The majority of papers had a low risk of bias, according to RoB 2 tool. Overall, the use of SDF is useful in the prevention and stabilization of caries and can be considered an important agent in caries arrest in school-age children.

**Conclusions**

The present systematic review has highlighted the use of SDF in preventing and arresting dentine caries in preschool children. Despite significant differences in treatment protocols, data was consistent on the benefits of SDF in caries management.

#### P36 - A Critical Appraisal of Bruxism in Children

##### Filipa Espingarda^1^, Patrícia Fonseca^1,2^, Patrícia Correia^1,2^

###### ^1^ Faculty of Dental Medicine, Universidade Católica Portuguesa, Viseu, Portugal; ^2^ Centro de Investigação Interdisciplinar em Saúde, Universidade Católica Portuguesa, Viseu, Portugal

####### **Correspondence:** Patrícia Correia (pcorreia@ucp.pt)

*BMC Proceedings 2023,* **17(9):**P36

**Background**

Bruxism has a higher prevalence in the younger population, comparing to the general population and it can occur at night or during the day. The aetiology of sleep or awake bruxism is multifactorial. Since the 2018 international consensus, bruxism has not been regarded a movement disorder or a sleep disorder in otherwise healthy individuals. However, in the literature, there is still variability in the diagnostic criteria, rendering it difficult to identify as a disorder. Besides these, there is also a lack of agreement on the most effective therapy. The aim of the present review was to critical appraise therapies for bruxism reduction in children.

**Materials and Methods**

A systematic review was designed based on the PRISMA guidelines and framed according to a PICO question. Several databases, such as PubMed/MEDLINE®, Embase and ProQuest, were included. Specific keywords and Boolean operators were applied. The search strategy was limited to children up to 12 years old, with no time limit, and studies in Portuguese and English languages. Two investigators performed the selection of studies independently. The quality of the studies was assessed using the Newcastle-Ottawa scale.

**Results**

The search resulted in 89 studies. After duplicates removal, the title and abstract were analyzed, leaving 32 for full text assessment. Ultimately, a total of 4 studies were considered in the qualitative analysis of this systematic review. The results showed that the use of occlusal splints with adequate follow-up was effective, photobiomodulation and physical therapy were reported as emerging interventions.

**Conclusions**

In the literature, bruxism management interventions in children include occlusal splints and alternative treatments, such as photobiomodulation and physical therapy. A high degree of heterogeneity was found due to diagnostic cofounders.

#### P37 - Children´s Oral Mucositis – perspectives from the parents and the literature

##### Francisca Marvão^1^, Joana Janeiro^1^, Ana Paula Fernandes^2^, Raquel Silva^1,3^, Patrícia Correia^1,3^

###### ^1^ Faculty of Dental Medicine, Universidade Católica Portuguesa, Viseu, Portugal; ^2^ Department of Peadiatric Oncology, Instituto Português de Oncologia Francisco Gentil, Porto, Portugal; ^3^ Centro de Investigação Interdisciplinar em Saúde, Universidade Católica Portuguesa, Viseu, Portugal

####### **Correspondence:** Patrícia Correia (pcorreia@ucp.pt)

*BMC Proceedings 2023,* **17(9):**P37

**Background**

Cancer-related oral mucositis (OM) affects mostly children and can seriously impact their nutritional intake, oral-care, quality of life and, at times, course of treatment. As main carers, parental knowledge is essential during OM episodes. There are several therapeutic options, which require clinical evidence. The purpose of this study was to report both parental literacy and evidence-based knowledge of OM.

**Materials and methods**

A parent questionnaire was used on a convenient sample, at a Paediatric Cancer Ward, addressing oral health and OM management. Additionally, a systematic review based on the PRISMA guidelines, was performed in PubMed/MEDLINE®, Embase and grey literature databases. Selection criteria were applied to retrieve articles in the last 20 years, in English and Portuguese.

**Results**

In our sample, cancer treatment related oral complications were high, however oral hygiene measures and dental professional support was not always available. In the literature review, a total of 11693 articles were obtained and after careful selection, according to pre-determined eligibility criteria, 6 articles were included. There is a growing body of evidence on the use of natural products in treating OM.

**Conclusions**

Oral health support should be given to parents of children being treated for cancer. There are several therapies in the management of OM, however evidence level varies. Good quality randomized control trials are still required. Providing better professional oral care and empowering the parents of children treated for cancer is paramount to improve management of these patients.

This study was kindly funded by the “*4ª Edição Prémio Rui Osório de Castro / Millennium BCP*”: https://froc.pt/4a-edicao-premio-rui-osorio-de-castro-millennium-bcp-vencedor-e-mencoes-honrosas/

#### P38 - Squamous cell carcinoma: apropos of a clinical case

##### Rúben Martins^1^, Tiago Marques^2^, Patrícia Couto^2^

###### ^1^ Faculdade de Medicina Dentária, Universidade Católica Portuguesa, Viseu, Portugal; ^2^ Center for Interdisciplinary Research in Health, Universidade Católica Portuguesa, Viseu, Portugal

####### **Correspondence:** Patrícia Couto (pscouto@ucp.pt)

*BMC Proceedings 2023,* **17(9):**P38

**Background**

Squamous Cell Carcinoma (SCC), also known as Epidermoid Carcinoma represents more than 90% of all malignant tumors that occur in the oral cavity. It mainly affects males, aged between 50 and 80 years. However, some studies show an increase in the development of this pathology in patients younger than 45 years. Tobacco use and alcohol consumption are well-established risk factors. However, a small proportion (15-20%) occurs in patients without a history of smoking and alcoholism, suggesting the presence of other risk factors.

**Case Report**

This paper reports the case of a female patient, 73 years old, with an ulcerated, fixed, indurated lesion, located on the right lateral-posterior border of the tongue, with clearly defined margins. The patient had no risk factors and believed she had a traumatic ulcer caused by tooth 47. After extraction of the tooth, there was no regression of the lesion. The treatment performed consisted of excisional biopsy. After total excision, the surgical specimen was sent for histopathological analysis, confirming the diagnosis of well-differentiated keratinizing squamous cell carcinoma, with invasion of the chorion, reaching the most superficial bundles of the muscle proper in the anatomical region. Because of the aggressive nature of the SCC and complex treatment options, the patient was referred to an oncology service for a strict follow-up.

**Conclusion**

In fact, squamous cell carcinoma represents the majority of tumors in the oral cavity and should be considered when there is an ulcerated lesion, with no history of traumatic factors, and which does not heal. In the case in question, the continuation of the symptoms after the extraction of the tooth ruled out the hypothesis of a traumatic lesion, which led to the performance of an excisional biopsy, dictating the diagnosis of squamous cell carcinoma. In short, even though there are no risk factors and little propensity at the gender level, all hypotheses must be considered. A good anamnesis, clinical and histopathological examination are always essential for a correct and definitive diagnosis.

Informed consent was obtained and all methods were performed in accordance with the Declaration of Helsinki principles for medical research involving human subjects and following the requirements established by Portuguese Law n.° 21/2014 for clinical research.

#### P39 - Verrucous carcinoma: apropos of a clinical case

##### Patrícia Couto^1^, Nélio Veiga^1^

###### ^1^ Center for Interdisciplinary Research in Health, Universidade Católica Portuguesa, Viseu, Portugal

####### **Correspondence:** Patrícia Couto (pscouto@ucp.pt)

*BMC Proceedings 2023,* **17(9):**P39

**Background**

Verrucous carcinoma is a low-grade variant of oral squamous cell carcinoma. It is more common in males, over 55 years old (65-70 years old) and is usually associated with chewing tobacco. Its preferred location is the bottom of the vestibule, gums, buccal mucosa, tongue and hard palate.

**Case Report**

This paper reports the case of a male brazilian patient, 67 years old, former tobacco user (about 20 years), diagnosed with metabolic syndrome and with a history of chronic alcoholism, sporadic depression and anxiety. The patient had a white, extensive lesion on the right lateral border of the tongue, with a long evolution time, well delimited, not removable by scraping, without painful symptoms and with verruciform projections on the surface. There was also an increase in cervical lymph nodes. The treatment performed consisted of incisional biopsy. The surgical specimen was sent for histopathological analysis, obtaining a provisional diagnosis of papillomatous squamous proliferation and suggesting the need of a differential diagnosis with verrucous carcinoma. Given the extent of the lesion, the patient was referred to the IPO (Portuguese Institute of Oncology) for histopathological examination of the entire lesion, which confirmed the definitive diagnosis of verrucous carcinoma. The patient has been subject to periodic follow ups in the last 5 years without any recurrence.

**Conclusion**

As seen in the present case, verrucous carcinoma usually appears extensive at the time of diagnosis, and in the form of a verrucous lesion, with slow and progressive growth, which histopathologically presents a misleading benign appearance. A good anamnesis (looking for risk factors), a detailed clinical examination that evaluates all aspects of a lesion (color; ulceration; bleeding; growth; consistency; duration; fixation) and an adequate histopathological examination are always essential for a correct and definitive diagnosis.

Informed consent was obtained and all methods were performed in accordance with the Declaration of Helsinki principles for medical research involving human subjects and following the requirements established by Portuguese Law n.° 21/2014 for clinical research.

#### P40 - The perception of occlusal plan inclination

##### Cristina Figueiredo^1^, Teresa Rosmaninho^1^, Ana Margarida Silva^2^, Tiago Marques^1^

###### ^1^ Universidade Católica Portuguesa, Faculty of Dental Medicine, Center for Interdisciplinary Research in Health, Portugal; ^2^ Faculty of Dental Medicine (FMD), Universidade Católica Portuguesa, Portugal

####### **Correspondence:** Cristina Figueiredo (cristinafigueiredo@ucp.pt)

*BMC Proceedings 2023,* **17(9):**P40

**Background**

The integration of dental prostheses into the patient's face and smile is a key factor for an aesthetically pleasing restorative outcome. The importance of dental and rehabilitation aesthetics has increased in the last decades, as a result of a greater demand from patients and dentists. Aesthetics and beauty are defined in the literature as a subject concept, resulting from the perception of each individual. The aim of this study is to measure the differences in perception between Laypeople, Dental Students and Dentists according to discrepancies in the occlusal plane inclination regarding the corresponding facial landmarks, as well as age.

**Materials and methods**

An observational Cross-Sectional study with a survey applied to 3 different populations was made. This study protocol was approved by the Ethics Commission for Health of the Universidade Católica Portuguesa (Report number 180, 21st January 2022). Informed consent was obtained from all participants. Two groups of symmetrical facial images were created for female and male gender. The occlusal plane was modified in1degree incremental angulation (from 0 to 5 degrees of angulation), and the position of maxillary central incisors was manipulated in order to follow the inclination of the occlusal plane.

**Results**

Statistically significant differences were found between the three populations (p<0,05). Dentists showed a higher rate of success identifying changes. Regarding age analysis, there were statistically significant results, especially in the elderly age group, which demonstrated a lower rate of perception for changes.

**Conclusions**

The level of knowledge and the age of the viewer proved to be statistically significant in the esthetic perception. It is essential that dentists have the necessary tools to evaluate which approach and esthetic criteria are the most suitable for the appropriate treatment of each patient.

#### P41 - Tooth supported overdenture: A case Report

##### Cristina Figueiredo^1^, Filipe Araujo^1^, Ana Margarida Silva^1^, Tiago Marques^1^

###### ^1^Universidade Católica Portuguesa, Faculty of Dental Medicine, Center for Interdisciplinary Research in Health, Portugal

####### **Correspondence:** Cristina Figueiredo (cristinafigueiredo@ucp.pt)

*BMC Proceedings 2023,* **17(9):**P41

**Background**

An overdenture is, by definition, any removable dental prosthesis that entirely covers one or more remaining natural teeth, root, or implant. The maintenance of dental roots allows the use of copings or/and precision attachments, and the retention of the denture is provided. The advantages of overdentures with natural roots are the preservation of the alveolar ridge, providing sensory feedback and improving the stability of dentures. Furthermore, this could be a treatment modality for elderly patients once adaptation and comfort to wearing dentures is facilitated.

**Case Report**

A 80-year-old man was referred for prosthodontic rehabilitation to the Faculty of Dental Medicine in Viseu. The chief complaint was chewing difficulty and poor esthetics. He presented an elevated degree of wear in both upper and lower jaws caused by attrition and erosion. The prosthetic rehabilitation was made with a superior overdenture with two precision attachments on both canines, with previous endodontic treatment, and a lower conventional removable dental prosthesis. The design of the Cr-Co framework included occlusal surfaces on the anterior teeth, the precision attachments in both canines and bar-type clasps on second pre-molars and a U-shaped palatal connector. Follow up appointments were made after 1, 3 and 6 months of rehabilitation. Fit, occlusion and oral hygiene were accessed and maintained. Functional and esthetic evaluation were performed, and patient’s comfort was ensured.

**Conclusion**

Overdentures may be the treatment of choice for some individuals, particularly when fixed rehabilitation can´t be considered (because of cost, systemic pathology, or lack of interest). This case report shows that this type of rehabilitation is easy to make, relatively cheap, conservative, and non-invasive. In controlled cases biological failures through caries, periodontal disease and endodontic problems will be rare and easy to manage.

Informed consent was obtained from the participant and all methods were performed in accordance with the Declaration of Helsinki principles for medical research involving human subjects and following the requirements established by Portuguese Law nr 21/2014 for clinical research.

#### P42 - Cone-beam computed tomography protocol to measure the upper airways – a contribution to the study of obstructive sleep apnea syndrome

##### Catarina Fonseca^1^, Vanessa Silva^1^, André Correia^1^, Patrícia Fonseca^1^

###### ^1^ Universidade Católica Portuguesa, Faculty of Dental Medicine, Center for Interdisciplinary Research in Health, Portugal

####### **Correspondence:** Catarina Fonseca (s-camefonseca@ucp.pt)

*BMC Proceedings 2023,* **17(9):**P42

**Background**

Analyze upper airway in Cone Beam Computerized Tomography (CBCT) can be useful for Obstructive Sleep Apnea Syndrome (OSAS) diagnosis [1]. In some individuals, upper airway obstruction results from airway narrowing and can be identified in CBCT [2].

**Objective**

The main objective of this research was to establish a protocol to evaluate the upper airway in CBCT.

**Materials and methods**

The study was approved by the Ethics Committee for Health of the University. The evaluation of the upper airways was performed in CBCT of patients attending the University Dental Clinic after informed consent. The studied images were obtained using Planmeca® ProMax 3D with an exposure of 90kV, 8mA and 13.713s. The software used was Romexis® version 5.1.O.R. Images were displayed according to field of view (FOV) of 20.1x17.4cm, size 502x502x436mm and voxel 400μm. The software tools were used to do the measurements.

**Results**

For airway measurement, the image was oriented in the 3 anatomical planes according to upper airway direction. After activating “Extract airways” tool, first point was marked at Posterior Nasal Spine level and airway delimitation was marked following its curvature, with last point marked at fourth cervical vertebra level.

“Airways tool” was selected and the variable that defines the image limit was adjusted to calculate airway dimensions. Calculation of the total volume and minimum area of the airway was done by the imaging software, minimizing errors. In the narrowest area, the anteroposterior and laterolateral dimensions were measured manually using the “linear measurement” tool. When necessary, the segmentation was readjusted to the entire airway.

**Conclusions**

This protocol allows automatic calculation of total volume of the pharyngeal air space, the area of greatest narrowing, its location and the smallest anteroposterior and laterolateral dimensions of the pharynx. The use of this protocol makes possible standardize measurements and assist in the identification of OSAS risk patients.

**References**

1. Zimmerman JN, Vora SR, Pliska BT. Reliability of upper airway assessment using CBCT. Eur J Orthod. 2019;41(1):101–8.

2. Gottlieb DJ, Punjabi NM. Diagnosis and Management of Obstructive Sleep Apnea: A Review. JAMA 2020;323(14):1389-40.

#### P43 - Evaluation of linear and angular deviations after implant placement with fully guided surgery using CoDiagnostix® - Clinical Case

##### Catarina Fonseca^1^, Margarida Quezada^1^, Patrícia Fonseca^1^, Tiago Marques^1^, André Correia^1^

###### ^1^ Universidade Católica Portuguesa, Faculty of Dental Medicine, Center for Interdisciplinary Research in Health, Portugal

####### **Correspondence:** Catarina Fonseca (s-camefonseca@ucp.pt); Margarida Quezada (s-mmquezada@ucp.pt)

*BMC Proceedings 2023,* **17(9):**P43

**Background**

With the advent of new technologies, it is possible to acquire patient’s data by means of a cone beam computed tomography (CBCT) and intraoral scanners. These tools allow the creation of a virtual 3D patient. This 3D data can be used in implant planning software (e.g. CoDiagnostix®) to establish a virtually treatment plan and manufacture surgical guides that allow a static computer-aided implant surgery (s-CAIS). According to the literature, s-CAIS allow the placement of implants with greater precision [1,2]. CoDiagnostix® software add-on “Treatment evaluation tool” allows the calculation of linear and angular deviations between the position initially planned for a dental implant and its real position after surgery.

**Case Report**

A 56-year-old male with no relevant pathology was observed in the Digital Oral Rehabilitation Post-Graduation Clinic to rehabilitate a single edentulous space in tooth 24 with a dental implant. After CBCT and intraoral scanner, the implant was virtually planned using CoDiagnostix® software (Straumann® Bone Level X Roxolid® SLActive® (RB) Ø 3.5mm 12.00 mm) and a surgical guide was developed to perform a fully guided surgery. The implant was placed following the guided surgery protocol of the manufacturer.

After surgery, the deviations between the virtually planned and the final position were measured in the “Treatment Evaluation Tool” of CodiagnostiX® software. The parameters evaluated were: angle of the implant with respect to the axial axis, mesio-distal and bucco-lingual deviation at the apex, mesio-distal and bucco-lingual deviation in the most coronal area of the implant and apico-coronal deviation. The following results were obtained: angle of the implant with respect to the axial axis =1.4 degrees; mesio-distal deviation at the apex = - 0.12 mm; bucco-lingual deviation at the apex = -0.15 mm; mesio-distal deviation in the coronal area = - 0.42 mm; bucco-lingual deviation in the coronal area = - 0.12 mm and apico-coronal deviation = 0.03 mm. The patient had a follow-up period of 3 months with no complications associated to the implant and after radiographic monitoring, the screwed crown was placed.

**Conclusion**

The linear and angular measurements between the virtually planned and the final position of the implant placed with guided surgery had no significant clinical relevance. This clinical case demonstrates the effectiveness of guided surgery and digital planning in oral rehabilitation.

The study protocol was approved by Ethics Commission for Health of the University (Comissão de Ética para a Saúde da Universidade Católica Portuguesa, Report number 201, 24th of March 2022).

Informed consent was obtained from the patient and all methods were performed in accordance with the Declaration of Helsinki principles for medical research involving human subjects and following the requirements established by Portuguese Law n° 21/2014 for clinical research.

**References**

1. Wismeijer D, Joda T, Flügge T, Fokas G et al. Group 5 ITI Consensus Report: Digital technologies. Clin Oral Implants Res. 2018 Oct;29 Suppl 16:436-442.

2. Siqueira R, Chen Z, Galli M et al. Does a fully digital workflow improve the accuracy of computer-assisted implant surgery in partially edentulous patients? A systematic review of clinical trials. Clin Implant Dent Relat Res. 2020 Dec;22(6):660-671.

#### P44 - Are mandibular advancement devices efficient in obstructive sleep apnea syndrome? A systematic review

##### Alexandra Caiado^1^, Vanessa Silva^2^, André Correia^2^, Patrícia Fonseca^2^

###### ^1^ Universidade Católica Portuguesa, Faculty of Dental Medicine (FMD), Viseu, Portugal; ^2^ Universidade Católica Portuguesa, Faculty of Dental Medicine, Center for Interdisciplinary Research in Health, Portugal

####### **Correspondence:** Patrícia Fonseca (pafonseca@ucp.pt)

*BMC Proceedings 2023,* **17(9):**P44

**Background**

Obstructive Sleep Apnea Syndrome (OSAS) is a sleep related respiratory disorder, classified by the Apnea/Hypopnea Index. One of the treatments recommended for the mild and moderate forms of this pathology are the Mandibular Advancement Devices (MAD).[1-3]

The aim of this study was to qualitatively assess the efficacy and the adverse effects of Mandibular Advancement Devices in the treatment of OSAS over time.

**Materials and methods**

This systematic review was developed using PRISMA guidelines. A search was carried out in the PubMed/MEDLINE®, Cochrane® and Web of Science-MEDLINE® databases, until April 15th, 2021. Two researchers selected the studies independently. The quality of the papers was assessed with Downs and Black checklist and the agreement between examiners was measured using the Cohen's kappa coefficient.

**Results**

Through the research, 2011 articles were identified. After removing duplicates and triplicates, the title of 662 articles was analyzed. Among these, 339 were examined by abstract and 62 by full reading. After this selection, 16 articles were eligible for analysis. Mandibular Advancement Devices increase the area of the airways through displacement of the mandible anteriorly and inferiorly, displacement of the soft palate, tongue, and hyoid bone anteriorly and activation of the masseter and submentonian muscles. These effects decrease the Apnea/Hypopnea Index and increase oxygen saturation. However, over time possible side effects emerge including reduced overjet and overbite, posteroinferior position of the mandible and joint discomfort.

**Conclusion**

MAD are effective in OSAS treatment reducing its symptoms. However, due to their intraoral placement, they can cause collateral effects that, despite not being considered a contraindication to treatment, should not be neglected.

**References**

1. Chang HP, Chen YF, Du JK. Obstructive sleep apnea treatment in adults. Kaohsiung J Med Sci. 2020;36(1):7-12.

2. Rashid NH, Zaghi S, Scapuccin M, Camacho M, Certal V, Capasso R. The Value of Oxygen Desaturation Index for Diagnosing Obstructive Sleep Apnea: A Systematic Review. Laryngoscope. 2021;131(2):440-7.

3. Alessandri-Bonetti A, Bortolotti F, Moreno-Hay I, Michelotti A, Cordaro M, Alessandri-Bonetti G, et al. Effects of mandibular advancement device for obstructive sleep apnea on temporomandibular disorders: A systematic review and meta-analysis. Sleep Med Rev. 2019;48:101211.

#### P45 - Digital technologies to check occlusal contacts assessed by dental students

##### Diana Sottomayor^1^, André Correia^1,2^, Patrícia Fonseca^1,2^

###### ^1^Universidade Católica Portuguesa, Faculty of Dental Medicine (FMD), Viseu, Portugal; ^2^Universidade Católica Portuguesa, Center for Interdisciplinary Research in Health (CIIS), Viseu, Portugal

####### **Correspondence:** Patrícia Fonseca (pafonseca@ucp.pt)

*BMC Proceedings 2023,* **17(9):**P45

**Background**

Evaluation of static and dynamic occlusal contacts is essential to Dentistry, particularly in oral rehabilitation treatments, but it is often difficult to perform and to objectively achieve.

The objective of this study was to assess the ability of Dental Medicine students to identify occlusal contacts (static and dynamic occlusion) with articular papers of different thicknesses, using digital occlusal records as reference.

**Materials and Methods**

A cross-sectional observational study was carried out after approval by the ethics committee of *Universidade Católica Portuguesa* (n° 201, 24 March 2022), with 60 undergraduate students, characterized by gender, from 4^th^ and 5^th^ year of Dental Medicine course. In a standard patient, each participant was asked to identify the main occlusal contacts in maximum intercuspation position and in excursive movements (protrusion and lateral movements) with 40μm and 200μm articular paper (Bausch®). These identifications were collected and recorded in a specific form and compared with the occlusal contacts records obtained digitally with the OccluSense® device. Data analysis was performed using IBM SPSS® software with a significance level of 0.05.

**Results**

There was no statistically significant relationship between gender and the identification of occlusal contacts regardless of the thickness of the articular paper used (p>0,005). Regarding maximum intercuspation position, most students correctly identified 3 of the 5 expected teeth (40%) with the 40μm paper. With the 200μm, the correct identification was lower, 2 of the 5 expected teeth (48.3%). The differences found between these two articular papers had statistical significance to the maximum intercuspation position (p=0,001). Regarding excursive movements, there was a greater ability to identify the correct contacts with the thicker articular paper but with no significance.

**Conclusions**

Dental students’ ability to identify occlusal contacts, static and dynamic, was influenced by the thickness of the articular paper and, in every case, the corrected identification was inferior to 50%. To make occlusal analysis more objective and didactic, digital evaluation of these contacts should be introduced in undergraduate education.

#### P46 - Can Thermography be used as a diagnostic tool in temporomandibular disorders? A systematic review

##### Luísa Silva^1^, Vanessa Silva^1^, André Correia^1,2^, Patrícia Fonseca^1,2^

###### ^1^ Universidade Católica Portuguesa, Faculty of Dental Medicine (FMD), Viseu, Portugal; ^2^ Universidade Católica Portuguesa, Center for Interdisciplinary Research in Health (CIIS), Viseu, Portugal;

####### **Correspondence:** Patrícia Fonseca (pafonseca@ucp.pt)

*BMC Proceedings 2023,* **17(9):**P46

**Background**

The application of infrared thermography (IT) in the diagnosis and follow-up of patients with Temporomandibular Disorders (TMD) is not consensual.

Therefore, the main objective of this study is to clarify the relevance/efficacy of IT application in the diagnosis of TMD.

**Materials and methods**

This systematic review was developed according to the standards described by PRISMA guidelines, the research question was formulated by PICO, and the research protocol was registered and validated. The research focused on 3 platforms of bibliographic databases: PubMed/MEDLINE®, Web of Science® and Embase®. The quality of the studies was evaluated through the STROBE checklist and the agreement between examiners was evaluated using Cohen's kappa coefficient. Data were collected regarding the date of performance, the population (age, gender sample size), the intervention (type of device and evaluation characteristics), the diagnostic/evaluation method and the main conclusions of each study included in this review and were analyzed in a comparative way.

**Results**

From the initial 170 articles obtained, 74 were duplicated and/or tripled, resulting in 96 articles to be screened. In the title screening, 75 articles were selected and later, 43 articles were selected by the abstract screening. Finally, a total of 12 final articles were selected in the full-text screening. Most of them were of high quality (83.33%), with a majority sample of women and aged between 18 and 40 years. The equipment and observation conditions are quite similar, and the areas of interest are mostly TMJ, temporal and masseter muscles. In general, studies conclude that the use of infrared thermography for the diagnosis of TMD demonstrated low accuracy and limited efficacy.

**Conclusions**

The lack of scientific evidence leads the authors to advise caution in the use of thermography in the diagnosis of temporomandibular disorders.

#### P47 - Proposal to the construction of clinical algorithm to support decision-making for the person with complex wounds

##### Raquel Marques^1,2^, Marcos Lopes^3^, Paulo Alves^1,2^

###### ^1^ Universidade Católica Portuguesa, Center for Interdisciplinary Research in Health, Portugal; ^2^ Universidade Católica Portuguesa, Institute of Health Sciences; Escola Enfermagem (Porto), Portugal; ^3^ Universidade Federal Ceará, Nursing School, Fortaleza, Brasil

####### **Correspondence:** Raquel Marques (s-raquelmarquessilva@ucp.pt)

*BMC Proceedings 2023,* **17(9):**P47

**Background**

Given the increasing complexity of evaluation and intervention in person with chronic, complex, or hard-to-heal wounds, healthcare professionals with less experience end up having a greater difficulty in the decision-making process, making it necessary to develop tools that support clinical reasoning in the diagnosis of wound typology and therapeutic plan.

**Objectives**

To construct and validate clinical algorithms for the differential diagnosis of wound typology and treatment of the person with wound.

**Material and methods**

Mixed study, composed of 3 stages. The first stage, construction of clinical algorithms supported in evidence produced in systematic reviews of the literature, in the opinion of experts and guidance standards. The second stage, content analysis of recommendations and alerts by consensus of expert/specialist to incorporate in clinical algorithms. The third stage, a clinical validation through analytical observational study of prospective and multicenter cohort to determine intra-observer and interobserver agreements and reliability for wound type diagnoses (pressure ulcer, pressure ulcer associated with medical devices, pressure ulcer in the mucous membranes, venous leg ulcer, mixed leg ulcer, arterial ulcer and diabetic foot ulcer) and treatment recommendations between nursing evaluation, system algorithm and expert/specialist.

**Results**

With this study we intend to validate the different components built for clinical algorithms, alerts and recommendations for the treatment of person with complex wounds. The decision support tools promote: safety in action, high quality healthcare and protection against complications. This study is in phase two of content validation with experts.

**Conclusions**

The results will give validity to the constructed algorithms that will integrate in a mobile application to support clinical decision-making that aims to guide the healthcare professional to provide standardized, safe, and evidence-based care.

#### P48 - Sequential surgical guide for full arch immediate implant placement and provisionalization in high risk patient

##### Tiago Marques^1,2^, Filipe Araújo^1,2^, Nuno Santos^1,2^, Patrícia Fonseca^1,2^, André Correia^1,2^

###### ^1^ Universidade Católica Portuguesa, Center for Interdisciplinary Research in Health, Viseu, Portugal; ^2^ Universidade Católica Portuguesa, Faculdade de Medicina Dentária, Viseu, Portugal

####### **Correspondence:** Tiago Marques (tmmarques@ucp.pt)

*BMC Proceedings 2023,* **17(9):**P48

**Background**

Incorporation of virtual engineering in Dentistry and the digitalization of information are giving new perspectives for dental treatments. Implant planning software allows the combination of radiology, prosthetic and surgical fields under a common virtual scenario, creating the designated virtual patient. This allows an optimized treatment planning and, when possible, the use of least invasive surgical techniques, with the patient experiencing a better postsurgical course with a faster tissue healing.[1]

**Case Report**

54 years-old male presented with a chief complaint of teeth advanced mobility and an ill-fitting maxillary removable partial denture. Patient was a former smoker, no relevant medical history, with a Stage III/B Periodontitis and diagnosed with bruxism. The case was classified with high risk according to the ITI® SAC classification. Treatment plan: rehabilitation of the maxilla with dental implants and an immediate loading with a provisional fixed prosthesis. To perform this treatment, sequential surgical guides were developed: 1– Tooth-supported guide to place anchor pins; 2– Tissue-supported surgical guide (after teeth extraction), retained with anchor pins to place dental implants (two of them, immediate); 3– Tissue-supported prosthetic guide, retained with anchor pins, to do a pick-up of provisional abutments and finalize the provisional prosthesis.

Three months after provisionalization patient initiated the procedures for the definitive zirconia monolithic prosthesis, with a titanium-bar framework. Then, a centric relation splint was done to protect the prosthesis and temporomandibular joints. Within a follow-up period of 1 year, the success/survival rate is 100%, and patient is very satisfied with the rehabilitation, that has an alpha score according to the modified USPHS criteria.

**Conclusion**

Sequential surgical guides to assist patients with severe periodontitis for immediate full arch implantation and immediate restoration can expand the indications of guide assisted implant surgery. It meets the safety requirements in clinical applications.

Informed consent from the patient was obtained to publish in open access.

**Reference**

1. Lanis A, Llorens P, Álvarez Del Canto O. Selecting the appropriate digital planning pathway for computer-guided implant surgery. Int J Comput Dent. 2017;20(1):75–85.

#### P49 - Computer Guided Bone Harvesting from Mandible. Case Series

##### Tiago Marques^1**,2**^**,** André Correia^1,2^, Filipe Araújo^1,2^, Nuno Santos^1,2^

###### ^1^ Universidade Católica Portuguesa, Center for Interdisciplinary Research in Health, Viseu, Portugal; ^2^ Universidade Católica Portuguesa, Faculdade de Medicina Dentária, Viseu, Portugal

####### **Correspondence:** Tiago Marques (tmmarques@ucp.pt)

*BMC Proceedings 2023,* **17(9):**P49

**Background**

During prosthetically driven restorations, optimal implant placement in the presence of alveolar defects is strongly dependent on bone augmentation procedures.[1] Autogenous bone is the most predictable material to support new bone formation, allowing for a higher bone survival rate and implant success.[1,2] Computer-guided bone harvesting, performed according to the protocol described by De Stavola et al. (2015), effectively translated the surgical plan into the surgical field, assisting the surgeon in performing the correct osteotomy and limiting the variability of the cut position due to skill factors.[1] The position, angulation, and depth of the osteotomy is controlled, optimizing the volume of the harvestable bone block while reducing the risk of damage to anatomical structures.[1–3]

**Case report**

Three patients in need of mandibular horizontal ridge augmentation due to bone atrophy were treated with autologous bone graft from the retromolar area using a computer guided bone harvesting guide. All anatomical structures such as the alveolar canal and dental roots were located using Materialise Mimics Innovation Suite and ideal bone-cutting planes were defined with secure surgical margins. The final guide was designed using Exocad GmbH software and were printed on Phrozen Mini 8k printer. Clinical wound healing was evaluated 8 days post surgically and 15 days for suture removal. None of the cases showed any kind of complication in a 4 month follow-up.

**Conclusion**

This case series clinical results confirm that this is a clinically proven technique allowing a minimal invasive procedure with satisfying clinical results. The use of digital planning simplifies the procedure and reduces the learning curve, in a reproductible way.

**References**

1. De Stavola L, Fincato A, Albiero AM. A computer-guided bone block harvesting procedure: a proof-of-principle case report and technical notes. Int J Oral Maxillofac Implants. 2015;30(6):1409–13.

2. De Stavola L, Fincato A, Bressan E, Gobbato L. Results of Computer-Guided Bone Block Harvesting from the Mandible: A Case Series. Int J Periodontics Restorative Dent. 2017;37(1):e111–9.

3. De Stavola L, Cristoforetti A, Fincato A, Nollo G, Ghensi P, Cantarutti A, et al. Accuracy and Technical Predictability of Computer Guided Bone Harvesting from the Mandible: A Cone-Beam CT Analysis in 22 Consecutive Patients. J Funct Biomater. 2022 Dec 10;13(4):292.

#### P50 - New surgical technique for second stage surgery in mandibular implants

##### Tiago Marques^1,2^**,** Nuno Santos ^1,2^, Manuel Sousa ^2^

###### ^1^ Universidade Católica Portuguesa, Center for Interdisciplinary Research in Health, Lisboa, Portugal; ^2^ Universidade Católica Portuguesa, Faculdade de Medicina Dentária, Viseu, Portugal

####### **Correspondence:** Tiago Marques (tmmarques@ucp.pt)

*BMC Proceedings 2023,* **17(9):**P50

**Background**

The potential effects of circumferential keratinized mucosa around dental implants on the long-term stability of peri-implant tissues remain controversial. Several publications suggest that an inadequate width and thickness of peri-implant keratinized mucosa may lead to higher plaque deposition, higher rates of mucosal inflammation, a higher risk of peri-implant alveolar bone loss, soft tissue dehiscence, and clinical attachment loss. Additionally, there is evidence that the width of peri-implant KM influences immunological parameters. In a case of peri-implant soft tissue deficiency, the knowledge of the appropriate surgical technique seems to be of utmost clinical relevance for planning the second-stage surgery.

**Case Report**

This case report describes the treatment of an implant overdenture case with two tissue level implants that lack lingual and vestibular keratinized mucosa treated with a newly developed technique and followed up for one year, the implants are stable without any mucositis. This new technique consists of rotating the remaining buccal keratinized tissue to the lingual while two free gingival grafts are used to augment the buccal mucosa. The partial-thickness dissection is carried on the implant on the buccal and a second step in the technique involves the harvesting of an autogenous free gingival graft from the contralateral side of the palate, the next step is to position and secure the newly acquired autogenous tissue in the created recipient site. This technique has the potential of augmenting the buccal soft-tissue around implants in the atrophic mandible of edentulous patients, and documents a stable result for over 12 months after restoration of the dental implants overcoming the main limitations of other techniques.

**Conclusion**

Typically, surgeons choose the surgical methods that they are most familiar with, but once a surgeon comprehends more than three surgical techniques, one can choose the best treatment according to the anatomical characteristics, and the needs of the patient.

The purpose of this case report is to demonstrate one method of addressing the problem of inadequate soft tissue and to introduce clinicians to a new minimally invasive treatment for mandibular implants lacking keratinized mucosa solving the limitations of other techniques.

#### P51 - Screw-retained surgical guide for implant placement

##### Tiago Marques^1,2^**,** Filipe Araújo^1,2^, Bruno Valentim^2^, Patrícia Fonseca^1,2^, André Correia^1,2^

###### ^1^ Universidade Católica Portuguesa, Center for Interdisciplinary Research in Health, Viseu, Portugal; ^2^ Universidade Católica Portuguesa, Faculdade de Medicina Dentária, Viseu, Portugal

####### **Correspondence:** Tiago Marques (tmmarques@ucp.pt)

*BMC Proceedings 2023,* **17(9):**P51

**Introduction**

For patients with extended edentulous areas, with existing implants, and in need of additional implant placement, the use of the osseointegrated implants for guide fixation seems to be a logical alternative. Current options for the fabrication of surgical guides in this type of cases, involve creating surgical guides that are supported by the teeth, by the teeth and mucosa or retained by fixation pins. Since these existing techniques involve inherent inaccuracies, particularly when supported by the mucosa, or by failing teeth, the fabrication of surgical guides that are screw-retained at the implant or abutment level would probably reduce those inaccuracies by stabilizing the guide.[1]

The purpose of the present technical report is to illustrate a step-by-step digitally planned guided implant placement protocol for terminal dentition patients with salvageable existing implants requiring full-arch implant rehabilitation or partial rehabilitation.

**Case series**

Five patients received eight implants using a screw-retained guide. None of the implants was immediate loaded. In two of the cases single implants were placed using only one screw retention. The other 3 cases were full-arch cases, where several screw-retained implants were used. All implant planning was done using COdiagnostix® and to make the guides screw retained Exocad GmbH software was used to attach the prosthetic connection. All guides were printed on a Phrozen Mini 8k printer using NextDent SG biocompatible resin.

The success rate was 100% after one year follow up and the final implant position, when compared to the planning, was within the acceptable clinical deviations values reported in the literature.

**Conclusion**

The purposed protocol seems to enhance the accuracy of guided implant placement with screw-retention, simplifying the transition from failing teeth to implants, and reducing chairside time. However, further studies are needed to corroborate the findings of this case series.

**Reference**

1. Papaspyridakos P, De Souza A, Kudara Y, Basha V, Bokhary A, Sinada N, et al. Screw-retained surgical guide for implant placement in terminal dentition patients with existing implants. J Prosthodont. 2022 Aug 14;31(7):639–43.

#### P52 - The effect of the interproximal creeping attachment with VISTA technique in the anterior mandible: A case report with 4 years follow up

##### Tiago Marques^1**,2**^, Nuno Santos^1,2^, Manuel Sousa^2^, Javier Montero^3^, André Correia^1,2^

###### ^1^ Universidade Católica Portuguesa, Center for Interdisciplinary Research in Health, Lisboa, Portugal; ^2^ Universidade Católica Portuguesa, Faculty of Dental Medicine, Viseu, Portugal; ^3^ Universidad de Salamanca, Faculty of Medicine, Department of Surgery, Salamanca, Spain

####### **Correspondence:** Tiago Marques (tmmarques@ucp.pt)

*BMC Proceedings 2023,* **17(9):**P52

**Background**

Gingival recession is a common manifestation in most populations. The mechanism by which gingival recession occurs is not well understood but it seems to be complex and multifactorial. The main etiological factors are the accumulation of dental plaque biofilm with the resulting inflammatory periodontal diseases and mechanical trauma due to faulty oral hygiene technique, especially in thin biotypes.[1] Over the time, after periodontal surgery, a known phenomenon named creeping attachment occurs, which increases the attached gingiva width around the tooth and stops the progressive GR.

**Case Report**

**Fig. 1 (abstract P52).**
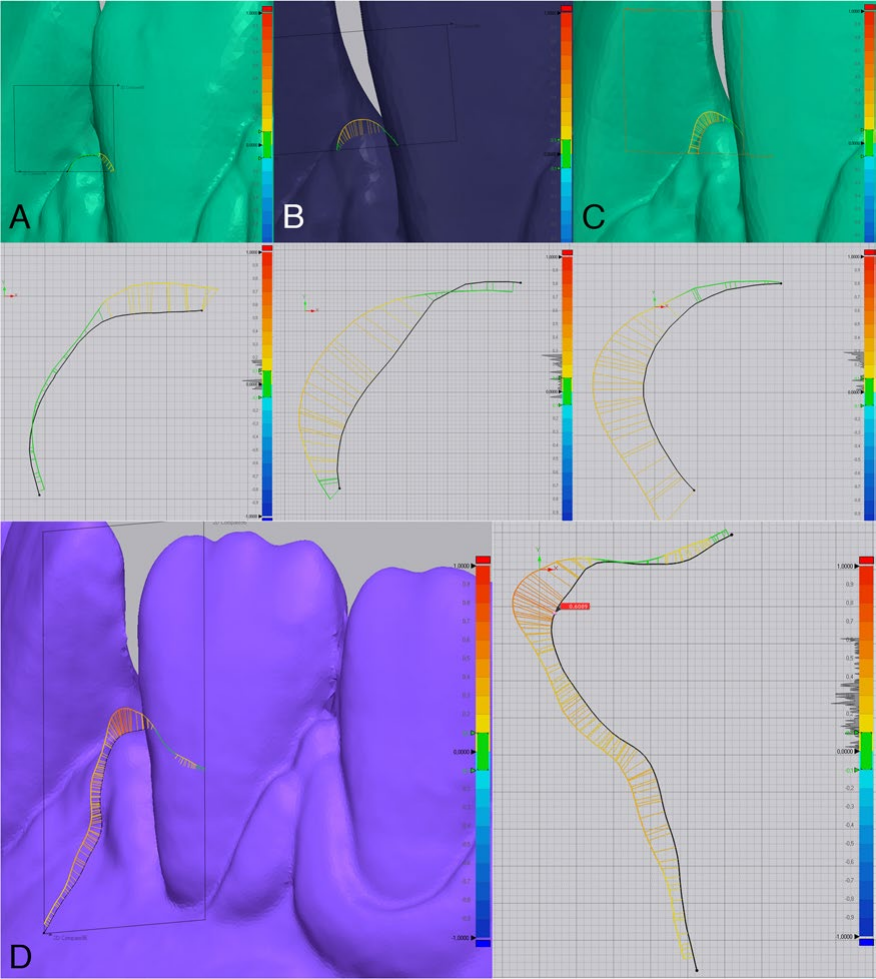
**A** Interproximal creep attachment at 3 months, **B** 3 months to 6 months, **C** 6 months to 48 Months, **D** Interproximal creep attachment from 3 months to 48 Months

This case report describes the treatment of a vestibular recession associated with interdental bone loss, with VISTA technique associated with a connective tissue graft. The case was evaluated at 3, 9 months and 48 months after the surgery clinically complete root coverage and increased thickness of keratinized tissue were achieved, and the interdental papilla was augmented improving the soft tissue quality for future orthodontic treatment. (Fig 1).

**Conclusion**

The purpose of the current case report was to digitally evaluate the creep attachment effect, either in buccal as interproximal sites, assessed on long-term (48 months), and to present the clinicians with the result obtained with a minimally invasive treatment using the tunnel technique. VISTA technique associated with a connective tissue graft to reconstruct vertically papilla is a promising alternative for minimally invasive treatment and stable after 4 years.

**Reference**

1. Marques T, Santos NM, Fialho J, Montero J, Correia A. A new digital evaluation protocol applied in a retrospective analysis of periodontal plastic surgery of gingival recessions. Sci Rep. 2021 Oct 14;11(1):20399.

#### P53 - Experiences of comfort of hospitalized person: scoping review

##### Ana Gonçalves Martins^1^, Rita Margarida Marques^1^, Patrícia Pontífice Sousa^1^

###### ^1^ Centro de Investigação Interdisciplinar em Saúde, Instituto de Ciências da Saúde, Universidade Católica Portuguesa, Lisboa, Portugal

####### **Correspondence:** Ana Gonçalves Martins (s-ailmmartins@ucp.pt)

*BMC Proceedings 2023,* **17(9):**P53

**Background**

In nursing, using a centred-care approach, understanding how comfort/discomfort is experienced will contribute to identify not only the patients` needs but also nursing interventions which may promote comfort. Both are often related, as they are applied as strategies, care styles, depending on nurse-patient relationship patterns or even professional behaviour. Nurses play a key role in promoting well-being and quality of life, hence why caring for individuals in a hospital setting means understanding people´s needs, their concerns, their anxieties, their experiences of comfort and discomfort, and implementing interventions that provide comfort. In nursing, providing comfort can be understood as the fulfilment of a specific need or discomfort felt, promoting a state of relief, calmness, or wholeness, in search of a state of Health. The objective of the study is to map patients´ experiences of comfort and discomfort in hospital settings within current literature.

**Materials and methods**

A Scoping review, using the Joanna Briggs Institute methodology, between 1990 and 2022.

**Results**

The absence of pain, physical structure, health and safety of health care organisations, the presence and support of family and friends, the promotion and respect for spirituality, and the technical qualification and affection of health professionals are mentioned by participants as shared experiences in different clinical settings. Pain appears as an ally of discomfort, often being a constant cause of lack of comfort for patients, admitted in hospital settings**.**

**Conclusions**

The perception of comfort can be shared by subjects, although there are specific elements which characterizes each individual experience. The diversity of settings of the studies reveals the existence of a specific concept of comfort, depending on the setting the subjects are involved with. The need for a patient-centred nursing care process, based on each one´s real experiences of comfort became clear, adopting interventions committed to satisfy the patients’ needs for comfort, improving the quality of the care provided.

**Keywords**

Patient comfort, nursing, hospitalization, review.

#### P54 - Spiritual distress in cancer patients undergoing chemotherapy: a longitudinal study

##### Helga Martins^1,2^, Joana Romeiro^1^, Sílvia Caldeira^1^

###### ^1^ Instituto de Ciências da Saúde, Universidade Católica Portuguesa, Center for Interdisciplinary Research in Health, Lisbon, Portugal; ^2^ Escola Superior de Saúde, Instituto Politécnico de Beja, Beja, Portugal

####### **Correspondence:** Helga Martins (s-htmartins@ucp.pt)

*BMC Proceedings 2023,* **17(9):**P54

**Background**

Spiritual distress is a nursing diagnosis related to suffering and lack of meaning/purpose in life. However, spiritual distress in clinical practice is often undervalued and neglected in nursing care. Cancer patients go through a complicated health situation in which the spiritual dimension is affected and experienced through spiritual distress. The aim of this study is to assess spiritual distress in patients undergoing chemotherapy treatment.

**Materials and methods**

A quantitative, longitudinal, and prospective study conducted in a Hospital Day setting. 332 participants were assessed for spiritual distress before initiating chemotherapy and at three months, six, nine, and twelve-month follow-ups. A questionnaire was used and the outcomes were sociodemographic characteristics, clinical condition, and spiritual distress (Spiritual Distress Scale). Data analyses included descriptive and bivariate statistics, which were conducted with SPSS. The study was approved by the Ethics Committee where the study was conducted.

**Results**

At baseline, 56.6% were females, mean age of 60.3 years (SD= ±11.7), and 66.3% were married. At three months, were reached the highest value of spiritual distress, and the score gradually decreased; however, the value of spiritual distress at 12 months was higher than at baseline. Trajectories did not differ significantly between man and women, but age and religious affiliation was predictor of spiritual distress. Spiritual distress exhibited a negative, weak, statistically significant correlation with religious involvement.

**Conclusions**

There is an important variation of spiritual distress at three months after the beginning of chemotherapy. These data reinforce the need for spiritual assessment in cancer patients to promote spiritual health and well-being.

#### P55 - Validation of instruments for assessing spiritual well-being, religious involvement, and spiritual distress: methodological studies

##### Helga Martins^1,2^, Joana Romeiro^1^, Sílvia Caldeira^1^

###### ^1^ Instituto de Ciências da Saúde, Universidade Católica Portuguesa, Center for Interdisciplinary Research in Health, Lisbon, Portugal; ^2^ Escola Superior de Saúde, Instituto Politécnico de Beja, Beja, Portugal

####### **Correspondence:** Helga Martins (s-htmartins@ucp.pt)

*BMC Proceedings 2023,* **17(9):**P55

**Background**

Spirituality is a dimension that is part of a holistic approach to the patient. However, in Portugal, there is currently a lack of validated instruments in the Portuguese cultural context.

In clinical practice, it is necessary to have appropriate tools to ensure the equivalence of the measure, regardless of the context in which it is used. However, the validation of an instrument is an intricate process requiring a high degree of methodological rigor.

The aim of this study was to validate instruments for assessing spiritual well-being, religious involvement, and spiritual distress in the context of oncology and in couples with reproductive health conditions.

**Materials and methods**

Methodological guidelines according to Sousa and Rojjanasrirat [1].

**Results**

Spiritual well-being questionnaire embraced a four-factors solution and 20 items with an overall Cronbach’s alpha=0.947; Religious involvement (Belief into Action scale) comprises a two-factor solution and nine items Reliability – with an overall Cronbach’s alpha=0.86; Spiritual distress scale comprises a four-factors solution and 30 items with an overall Cronbach’s alpha=0.91.

**Conclusions**

This process was achieved through a rigorous methodological approach. This way, reliable instruments were validated and considered reliable to evaluate these concepts in Portuguese samples.

Therefore, instruments for assessing spiritual well-being, religious involvement, and spiritual distress in different contexts provide nurses with the necessary resources and tools for measuring and evaluating spiritual and religious needs to plan appropriate nursing interventions, obtain better patient health outcomes and increase the quality of nursing care.

However, this is an underdeveloped area, and there are still few scales available in clinical practice that claim to need further development and investment.

**Reference**

1. Sousa VD, Rojjanasrirat W. Translation, adaptation and validation of instruments or scales for use in cross-cultural health care research: a clear and user-friendly guideline. J Eval Clin Pract. 2011;17:268-274.

#### P56 - Religious commitment of cancer patients after one year of chemotherapy: a cross-sectional study

##### Helga Martins^1,2^, Joana Romeiro^1^, Sílvia Caldeira^1^

###### ^1^ Instituto de Ciências da Saúde, Universidade Católica Portuguesa, Center for Interdisciplinary Research in Health, Lisbon, Portugal; ^2^ Escola Superior de Saúde, Instituto Politécnico de Beja, Beja, Portugal

####### **Correspondence:** Helga Martins (s-htmartins@ucp.pt)

*BMC Proceedings 2023,* **17(9):**P56

**Background**

Cancer patients during their disease face several treatments, one of them is chemotherapy. One of the coping strategies to overcome this process used by these patients is religious commitment. Religious commitment reduces stress, anxiety and pain related with treatments. The aim of this study is to assess religious commitment in cancer patients after one year of chemotherapy.

**Materials and methods**

This study is a quantitative, observational, correlational and cross-sectional, involving 274 outpatients with cancer in a hospital which were involved in the study through a random sample technique. Data were collected after one year of chemotherapy treatment and it was used a questionnaire which gathered sociodemographic characteristics, clinical conditions and the Belief into Action (BIAC) scale. Data analysis was performed through SPSS software. The study was approved by the Ethics Committee of the hospital.

**Results**

274 participants were recruited (females *n*=167; males *n*=107), most were married (*n*=188) and had a religious affiliation (*n*=262). Mean score of BIAC was 26.28 (SD=±11.96). It was possible to achieve statically significant differences between males and females (U = 5383.000; *p* < 0.001) regarding religious commitment. In fact, females presented higher religious commitment (Mean Ranks= 158.77 than males (Mean Ranks= 104.31). However, this study did not achieve any statically differences between other sociodemographic and clinical condition variables and religious commitment.

**Conclusions**

After one chemotherapy treatment females’ cancer patients experience a higher religious commitment than males. Therefore, gender plays an important role regarding religious commitment, and as such it is necessary to pay special attention to men regarding the coping strategies they use in this health/disease process.

#### P57 - Hopelessness a predictor of spiritual distress nursing diagnosis in cancer patients: a follow-up study

##### Helga Martins^1,2^, Joana Romeiro^1^, Sílvia Caldeira^1^

###### ^1^ Instituto de Ciências da Saúde, Universidade Católica Portuguesa, Center for Interdisciplinary Research in Health, Lisbon, Portugal; ^2^ Escola Superior de Saúde, Instituto Politécnico de Beja, Beja, Portugal

####### **Correspondence:** Helga Martins (s-htmartins@ucp.pt)

*BMC Proceedings 2023,* **17(9):**P57

**Background**

Spiritual distress is defined as a lack of meaning in life, disconnection, suffering and anger at God. Also, spiritual distress, is a nursing diagnosis according to NANDA-International, Inc, which hopelessness is one of the defining characteristics. The aim of this study was to assess hopelessness as a predictor of in spiritual distress nursing diagnosis in cancer patients during chemotherapy treatment.

**Materials and methods**

A quantitative, observational and follow up study. It was comprised 322 outpatients with cancer followed at an Oncology Unit, randomly selected. The data was collected when patients were initiating chemotherapy and then quarterly until completing one year of chemotherapy. In addition, data collection occurred between February 2019 and May 2015, and was applied a questionnaire. The data was analyzed statistically through the program SPSS version 21. This study, was approved by the hospital´s Ethics Committee where the study was conducted.

**Results**

The initial sample embraced 188 females and 144 males. The dropout rate of this study reached 17,5% after twelve months. The age range was between 22 to 83 years old. Most of the patients had breast cancer (n=27.70%), colorectal cancer (n=23.7%) and lung cancer (n=14.8%). The frequency of hopelessness during the chemotherapy increased its value, such as before chemotherapy (n=55, 10.5%), after three months (n= 40, 12.7%), after six months (n= 41, 13.5%), after nine months (n= 40, 14.0%) and after twelve months (n= 42, 15.3%). Hopelessness was a predictor of spiritual distress after nine (ꞵ=3.254, *p*=0.029) and twelve months (ꞵ=3.461, *p*=0.019) since the beginning of chemotherapy.

**Conclusions**

At the end of nine months after the start of chemotherapy, hopelessness appears as a predictive variable for the diagnosis of spiritual distress, therefore nurses can anticipate autonomous nursing interventions with the purpose for the readiness for enhanced hope in cancer patients.

#### P58 - Q methodology in research on the health of vulnerable groups

##### Susana Miguel^1^, Rita Silva^1^, Joana Bragança^1^, Silvia Caldeira^1^

###### ^1^ Institute of Health Sciences , Universidade Católica Portuguesa, Lisbon, Portugal

####### **Correspondence:** Susana Miguel (s-ssamiguel@ucp.pt)

*BMC Proceedings 2023,* **17(9):**P58

**Background**

Patients with poor or no voice quality or difficulty articulating are group vulnerable. They are often excluded from research due to their inability to be interviewed. Researchers should find the best method to fit the research goals and dignity-preserving rules when conducting research with vulnerable participants. The Q methodology may be appropriate and inclusive method for this specific population.

**Material and methods**

Discussion about the adequacy of the Q methodology as an option with vulnerable participants based on the research developed with head and neck cancer patients undergoing surgery.

**Results**

This research method combines qualitative and quantitative procedures, which require participants to order statements written on cards according to their preference to ask a research question. It is often described as the method to study subjectivity. The Q methodology allows the study of subjective phenomena in clinical and non-clinical topics in nursing and health by providing an objective dimension for analyzing ideas, attitudes, and perceptions. The Q methodology includes five steps: definition of concourse, Q sample, P sample or P set, Q sorting, and analysis and interpretation. During this process, the participant doesn´t need to have verbal speech.

**Conclusions**

The Q methodology involves the participant reflecting on a given topic under study, diverging from other types of instruments, such as questionnaires, in which the response may have greater impulsiveness. The participant's involvement can lead him to understand that the answers he presents are individual and represent his personal view of the situation. It allows the health researcher to gain knowledge of certain phenomena in vulnerable groups that could usually be excluded from research.

#### P59 - Resilience in the elderly: accuracy study of a nursing outcome

##### Susana Miguel^1^, Gabriella Santos Lima^1,2^; Sílvia Caldeira^1^; Luciana Kusumota^3^

###### ^1^ Instituto de Ciências da Saúde, Centro de Pesquisa Interdisciplinar em Saúde, Universidade Católica Portuguesa, Lisboa, Portugal; ^2^ Programa de Pós-graduação em Enfermagem Fundamental, Escola de Enfermagem de Ribeirão Preto, Universidade de São Paulo, São Paulo, Brasil; ^3^ Departamento Enfermagem Geral e Especializada, Escola de Enfermagem de Ribeirão Preto, Universidade de São Paulo, São Paulo, Brasil

####### **Correspondence:** Susana Miguel (susanasamiguel@gmail.com)

*BMC Proceedings 2023,* **17(9):**P59

**Background**

Elderly or older adults are characterized by their resilient potential, which develops throughout life and is a protective factor in old age, and resilience is included in the Nursing Outcomes Classification (1). This study to aimed to analyze the accuracy of the elements of the nursing outcome “Personal resilience: elderly” in elderly.

**Materials and methods**

The methodological study was developed in three stages. First, the concept analysis (2) identified the attributes, antecedents, consequences, and empirical elements of “resilience in the elderly.” Next, a focus group (3) with experts verified the suitability and recommendation of the title, definition, and indicators of the nursing outcome. Lastly, a clinical study (4) with older adults, through a structured interview with an approach to resilient aspects (meaning, behaviors, and attitudes) throughout life and in old age. The study was approved by the Research Ethics Committee of the Nursing School of Ribeirão Preto, University of São Paulo and all participants signed the term of free and informed consent.

**Results**

For the concept "resilience in the elderly," the definition was "positive attitudes of the elderly, with the help of available resources in the face of adversity experiences". For the focus group, eight experts participated in a virtual environment. The discussions permeated the opinion and the degree of adequacy and representativeness of the proposed indicators (components of mental health; perspective and experience of aging; grief and loss experience; coping strategies; health perspective; optimistic perspective) to the nursing outcome. The clinical study included 25 participants, 82.3% were female, mean age 74.7 (SD=5.9), 52.9% were married and 41.1% widowed, mean education 3.0 (SD=2.5) years, 76.4% were catholics religion and had an average of 7.2 (SD=2.8) medical diagnostic; Mini Mental State Examination 26.2 (SD=24) points. The interviews took place under audio recording, exploring resilience in meaning, characteristics, and personal level in old age. Direct content analysis identified three levels of categories that concern about resilience in the elderly: “perception” represented the meaning of resilience for the elderly; “state” composed the characteristics of resilience attributed by the elderly and “behavior” examples of resilient attitudes in the face of adversity situations in the past and present of the elderly.

**Conclusions**

The accuracy and validation measures of the nursing outcome “Personal resilience: elderly” suggested more accurate indicators of the complexity of care for the elderly.

**References**

1. Moorhead S, Swanson E, Johnson M, Maas M (Eds). Nursing outcomes classification (NOC): measurement of health outcomes (6th ed.). St. Louis, MO: Elsevier.2018.

2. Rodgers BL. Concept analysis: An evolutionary view. In: Rodgers BL. & Knalf KA (Eds.), Concept development in nursing: foundations, techniques, and applications (pp. 77–102). Philadelphia: Saunders.2000.

3. Krueger RA; Casey MA. Focus groups: a practical guide for applied research. 5ed. Los Angeles: SAFE; 2015.

4. Bardin L. Análise de conteúdo. São Paulo: Edições 70, 2016.

#### P60 - Dental material used for teeth protection and treatment with structural changes into the enamel. An umbrella review

##### Andreia Cristina Gomes^1^, Pedro C. Lopes^1,2^, Thais Gimenez^3^, Patrícia Nunes Correia^1,2^, Anna Carolina Volpi Mello-Moura^1,2^

###### ^1^ Universidade Católica Portuguesa, Faculdade de Medicina Dentária, Viseu, Portugal; ^2^ Universidade Católica Portuguesa, Center for Interdisciplinary Research in Health, Lisboa, Portugal; ^3^ Universidade Metropolitana de Santos, Mestrado em Saúde e Meio Ambiente, Santos, São Paulo

####### **Correspondence:** Anna Carolina Volpi Mello-Moura (acmoura@ucp.pt)

*BMC Proceedings 2023,* **17(9):**P60

**Introduction**

Dental enamel can undergo changes at different stages of amelogenesis because of the interplay of various factors both at the systemic, environmental and hereditary levels. Consequently, several types of enamel defects such as molar incisor hypomineralization (MIH), fluorosis and amelogenesis imperfecta (AI) can arise.

**Materials and Methods**

A systemic umbrella review was performed following the PRISMA guidelines. Results of searches up to April 2022 of the electronic databases Pubmed, Scopus and Web of Science were included in the current study which aimed at attempting to answer the question as to which materials were most suitable for the protection/treatment of permanent molars with enamel alterations, based on the Pico research scientific strategy. After establishing search strategy, studies were selected with defined inclusion and exclusion criteria. The data extracted and calibration was finalized by two independent reviewers. The methodological quality was evaluated by means of the AMSTAR-2 tool and the risk of bias of the systematic review was assessed with the ROBIS tool.

**Results**

In all, 313 articles were identified during the search of the three databases plus a complementary manual search. After excluding duplicates, 279 articles were evaluated based on their titles and abstracts. Of these, 15 articles were eventually selected for complete reading in order to assess their eligibility. A total of seven studies met the pre-defined criteria and were deemed suitable for inclusion in the current systematic review.

**Conclusion**

The options currently available for the treatment of teeth with changes in their enamel structure vary from prevention, restoration or even extraction. Although the results of the present study suggest certain treatment options, there is evidently a need for further clinical studies, which might suggest with a higher degree of confidence, which materials are most suitable for the protection/treatment of permanent molars with enamel alterations.

#### P61 - Relationship between functional capacity and executive functions in Schizophrenia

##### Tomé Nazaré^1^, Filipa Ribeiro^1,2^

###### ^1^ Universidade Católica Portuguesa, Instituto de Ciências da Saúde, Lisboa, Portugal; ^2^ Centre for Interdisciplinary Research in Health, Lisboa, Portugal

####### **Correspondence:** Tomé Nazaré (tomenazare61@gmail.com)

*BMC Proceedings 2023,* **17(9):**P61

**Background**

functional and executive deficits are well documented in schizophrenic patients. Not so well documented is the relationship between the different executive functions (EF), namely working memory (WM), inhibitory control and cognitive flexibility, and the performance of instrumental activities of daily living (IADL). Performance-based instruments, like the UPSA-2, promote increased reliability and ecological validity in assessing IADL. IADL characterization and the knowledge of the relationship between EF and IADL can assist clinicians in predicting patients’ needs and planning cognitive interventions to promote their independence.

**Materials and methods**

the aim was to study the validity of UPSA-2 to detect functional deficits in people with schizophrenia and the relationship between EF and functional capacity in the same population. The project was approved by the ethics committees of the psychiatric institutions and all participants signed an informed consent. The sample consisted of a clinical group (n=37) and a control group (n=27). The UPSA-2 was used to assess the functional capacity; the Corsi Board, the Trail Making Test, the Digit Span, and the Stroop Test were used to assess the EF.

**Results**

UPSA-2 results significantly discriminate between patients and controls (*U*=935.50; *p*<0.001); EF are moderate to strongly correlated with UPSA-2 total score (*r*=0.558-0.756; *p*<0.001), with visuospatial WM showing to the strongest correlations, especially with the organization/planning domain (*r*=0.746; *p*<0.001); visuospatial WM and verbal WM are good predictors of UPSA-2 total score.

**Conclusions**

the results are in agreement with the literature regarding the good discriminative validity of the UPSA-2 in this population, proving to be a useful instrument to assess functional capacity. EF, especially visuospatial WM, also appears in the literature with a strong association and predictive value in relation to functionality. Including both an EF and functional capacity assessment in this population would allow for a good characterization of the functional profile of each subject, leading to individualized and effective therapeutic planning.

**Keywords**

Functional Capacity; Executive Functions; Schizophrenia; Performance Based Instruments; UPSA-2

#### P62 - Multimodal sensorial stimulus in the sense of embodiment during brain machine interface: a systematic review

##### Diogo Tomás^1^, Miguel Pais-Vieira^2,3^, Carla Pais-Vieira^3^

###### ^1^ Escola Superior de Saúde Atlântica, Barcarena, Portugal; ^2^ iBiMED - Instituto de Biomedicina, Departamento de Ciências Médicas, Universidade de Aveiro, Aveiro, Portugal; ^3^ CIIS - Centro de Investigação Interdisciplinar em Saúde Porto, Universidade Católica Portuguesa, Porto, Portugal

####### **Correspondence:** Carla Pais-Vieira

*BMC Proceedings 2023,* **17(9):**P62

**Background**

The Sense of Embodiment (SoE) allows a person to perceive and control actions of different body parts and plays an important role in neurorehabilitation. Namely, the SoE can be induced in real-time through the use of Brain-Machine Interface (BMI) technologies that allow the user having the experience of control and perception of a virtual body or body parts. The objective of the present systematic review was to identify the contribution of multimodal sensory stimulation while performing BMI-based tasks in studies integrating SoE variables.

**Materials and methods**

A systematic review was conducted to identify if studies integrating SoE variables have analyzed the contribution of multimodal sensory stimulation. Inclusion criteria were: i) research articles, ii) in English, iii) including brain-machine interfaces, iv) analyzing the Sense of Embodiment.

**Results**

From a total of 493 results, 223 were duplicates, 2 were in foreign languages, 86 were conference papers, and 1 was not available. From the remaining 181 articles searched, only 20 analyzed the SoE of humans using BMIs. Analysis of these articles revealed that most studies that relate BMI with sensorial stimulation with SoE are focused on the manipulation of visual stimuli, more specifically with its coherence (e.g., synchronous vs. asynchronous stimuli) and its realism (e.g., humanoid or robotic appearance). No study has analyzed the independent contributions of the different sensorial modalities.

**Conclusions**

These results support the notion that describing the independent and joint contributions of the different sensorial modalities to the SoE during BMI control may be relevant for neurorehabilitation protocols.

#### P63 - Analysis of information transfer during acquisition of a multimodal Brain-machine interface

##### João Martim Reis^1^, Miguel Pais-Vieira^1^, Carla Pais-Vieira^2^

###### ^1^ iBiMED - Instituto de Biomedicina, Departamento de Ciências Médicas, Universidade de Aveiro, Aveiro, Portugal; ^2^ CIIS - Centro de Investigação Interdisciplinar em Saúde Porto, Universidade Católica Portuguesa, Porto, Portugal

####### **Correspondence:** Carla Pais-Vieira

*BMC Proceedings 2023,* **17(9):**P63

**Background**

Brain-machine interface (BMI) control often requires simultaneously sensory, motor and cognitive processing and is associated with high information transfer between different neural circuits. Although many BMI applications have been developed in the last two decades, a description of the underlying neural mechanisms associated with the amount of information transferred between different networks of electrodes has not yet been thoroughly explored. To investigate potential relations between neural activity and information processing networks, neural data from a complete Spinal Cord Injury (SCI) patient controlling a multimodal BMI was analysed.

**Materials and Methods**

Informed consent was obtained from a SCI patient (Hospital Senhora da Oliveira Ethics Committee; no 15/2020) with a complete spinal cord lesion (according to the American Spinal Injury Association Impairment Scale) at the level of the 4th thoracic vertebra, and with history of chronic low back pain. A total of 5 sessions were analysed. The period of acquisition (i.e., when the patient was thinking about moving, but not actually performing the task) was analysed. Granger causality (GC) tests were performed to identify the network of electrodes transferring information to and from the electrodes recording from the scalp above the somatosensory cortex (C3 and C4).

**Results**

Analysis of GC revealed an extensive and asymmetrical network of electrodes sending and receiving information from the electrodes C3 and C4. The C3 electrode sent information in all sessions to the right prefrontal (Fp2), Central (Cz), right parietal (P4) and left occipital (O1) electrodes. Meanwhile a much more extensive network was found in the C4 electrode, namely involving the right prefrontal (Fp2), left and right frontal (F3 and F4), Central (Cz), left and right temporal (T3 and T4), as well as left and central parietal (P3, Pz) electrodes. The lowest information transfer from the C3 and C4 electrodes was found, in both cases, towards the right occipital electrode (O2).

**Conclusions**

These results support the notion that brain-machine interface control in a SCI patient is associated with information transfer in a complex network of electrodes recording from pre-frontal, frontal, central, temporal, and parietal regions of the scalp.

Informed consent was obtained for publication.

#### P64 - Electrophysiological activity and asymmetry in information transfer during brain-machine interface control by a spinal cord injury patient

##### José Gabriel Figueiredo^1^, Sandra Vieira^1^, Miguel Pais-Vieira^1^, Carla Pais-Vieira^2^

###### ^1^ iBiMED - Instituto de Biomedicina, Departamento de Ciências Médicas, Universidade de Aveiro, Aveiro, Portugal; ^2^ CIIS - Centro de Investigação Interdisciplinar em Saúde Porto, Universidade Católica Portuguesa, Porto, Portugal

####### **Correspondence:** Carla Pais-Vieira

*BMC Proceedings 2023,* **17(9):**P64

**Background**

Spinal cord injury (SCI) is associated with impairment of sensorimotor functions, reduced autonomy and quality of life of patients. Recent neurorehabilitation protocols, combining brain-machine interfaces (BMIs) with virtual reality and multimodal feedback, have achieved relevant improvements in sensorimotor and other functions. Neuroplasticity is elicited when coherent and synchronized inputs, generated by the multimodal feedback, are combined with motor imagery outputs generated by the central nervous system. These improvements are thought to be associated with asymmetries in neural activity during brain-machine interface control. Here, we have analysed symmetry in the network of information transfer associated with electroencephalography activity recorded from the scalp of a SCI patient training with a multimodal BMI.

**Materials and methods**

Informed consent was obtained from a SCI patient (Hospital Senhora da Oliveira Ethics Committee; no 15/2020) with a complete spinal cord lesion (according to the American Spinal Injury Association Impairment Scale) at the level of the 4th thoracic vertebra, and with history of chronic low back pain. First, neural recordings (electroencephalography) were obtained during the acquisition phase of brain-machine interface sessions (n=5) combining virtual reality with thermal, and tactile feedback. Second, Granger causality (GC) tests were performed and the number of significant connections between an electrode and the remaining electrodes was analysed. Lastly, the number of significant connections was compared between electrodes with symmetrical positions (pre-frontal; frontal, central, temporal, parietal, and occipital).

**Results**

Comparison of asymmetry in information transfer revealed that frontal (F3 and F4) and occipital (O1 and O2) electrodes presented the largest asymmetries in information transfer. Meanwhile, the parietal electrodes (P3 and P4) presented the lowest asymmetry in information transfer, immediately followed by the electrodes recording from central (C3, C4) and temporal (T3, T4) regions.

**Conclusions**

Asymmetries in information transfer were present during brain-machine interface control by a spinal cord injury patient and occurred mostly in occipital and frontal electrodes, while symmetrical patterns occurred in central and parietal electrodes.

Informed consent was obtained for publication.

#### P65 - Exoskeleton users prefer controlling an exoskeleton delivering real-time tactile feedback

##### Mafalda Aguiar^1^, Demétrio Matos^2^, André Perrotta^3^, Miguel Pais-Vieira^1^, Carla Pais-Vieira^4^

###### ^1^ iBiMED - Instituto de Biomedicina, Departamento de Ciências Médicas, Universidade de Aveiro, Aveiro, Portugal; ^2^ ID+ (Instituto de Investigação em Design, Média e Cultura), Instituto Politécnico do Cávado e do Ave, Vila Frescainha, Portugal; ^3^ Centre for Informatics and Systems of the University of Coimbra (CISUC), Coimbra, Portugal; ^4^ CIIS - Centro de Investigação Interdisciplinar em Saúde Porto, Universidade Católica Portuguesa, Porto, Portugal

####### **Correspondence:** Carla Pais-Vieira

*BMC Proceedings 2023,* **17(9):**P65

**Background**

Real-time tactile feedback during exoskeleton operation has been previously used for neurorehabilitation purposes, particularly through the delivery of tactile stimuli to the forearms in spinal cord injury patients when the soles of the exoskeleton touch the ground. It is not known however, if delivering this type of feedback in control subjects affects exoskeleton operation.

**Materials and methods**

Here, we compared users’ preference and self-reported performance indicators in a small sample of control subjects (N=7) performing different exoskeleton benchmarking scenarios with and without tactile feedback. Informed consent was obtained from participants (Committee for Health Sciences of the Universidade Católica Portuguesa (99/2022). For this subjects’ psychological performance indicators (PPIs) (Usability, Perceptibility, Acceptability, Functionality, Fatigue, Stress, Energy expenditure, and Attention) were assessed while they controlled the exoskeleton in a benchmarking scenario in five different versions: (flat, M-shape, A-shape, V-shape, and random shapes) with and without tactile feedback. In addition to the PPIs, a brief interview was conducted after each run to determine if users preferred controlling the exoskeleton with or without real-time tactile feedback.

**Results**

During the interviews 85.7% (6/7) of the subjects reported that using the tactile feedback facilitated exoskeleton control. Analysis of PPIs revealed a tendency (Interaction effect, P=0.09) for an increase in Energy expenditure in the Random scenario. This finding was supported by the results of the brief interviews. No differences, however, were found for Fatigue (P=0.65) and Stress (P=0.22) nor for the remaining PPIs (Acceptability, P=0.20; Usability, P=0.82; Perceptibility, P=0.92; Functionality, P=1.0; Attention, P=0.81).

**Conclusions**

These results support the notion that subjects prefer controlling an exoskeleton that delivers real-time tactile feedback to the forearms even though no significant differences were present in PPIs. Future studies, with larger samples and additional neuropsychological measurements, will be critical to determine to which extent different versions of the benchmarking scenario affect different PPIs.

#### P66 - Neural activity and pain variation in a spinal cord injury patient during brain-machine interface control

##### Márcia Gato^1^, Miguel Pais-Vieira^1^, Carla Pais-Vieira^2^

###### ^1^ iBiMED - Instituto de Biomedicina, Departamento de Ciências Médicas, Universidade de Aveiro, Aveiro, Portugal; ^2^ CIIS - Centro de Investigação Interdisciplinar em Saúde Porto, Universidade Católica Portuguesa, Porto, Portugal

####### **Correspondence:** Carla Pais-Vieira

*BMC Proceedings 2023,* **17(9):**P66

**Background**

Spinal cord injury (SCI) is associated with high prevalence of pain. In a recent study, we have demonstrated that continuous use of a multimodal brain-machine interface (BMI) resulted in self-reported pain reduction. BMIs have been previously used to reduce pain, however the underlying mechanism is not fully understood. For example, in some studies pain levels were reduced during the period of the BMI session (possibly due to an effect of attention) while in our study the self-reported pain reduction occurred weeks after the BMI protocol.

**Materials and methods**

To investigate potential relations between neural activity and ongoing levels of pain, we have analyzed neural data from a complete SCI patient while controlling a multimodal BMI. Informed consent was obtained from the participant (Hospital Senhora da Oliveira Ethics Committee; no 15/2020) with a complete spinal cord lesion (according to the American Spinal Injury Association Impairment Scale) at the level of the 4^th^ thoracic vertebra, and with history of chronic low back pain. The period of acquisition (i.e., when the patient was thinking about moving, but not actually performing the task), from a total of 18 sessions of patient, was analyzed. The power for the different frequency bands (delta, theta, alpha, beta, and gamma) was compared with two different self-reported pain scales, the visual analogue and the faces pain scale.

**Results**

Moderate negative Spearman Rho values were found for the right occipital electrode namely for beta (Rho=-0.63,) and gamma (Rho-0.62,) frequency bands. These, however, were not significant after multiple comparison corrections (beta: p=0.0049, n.s. and gamma: p=0.006, n.s.).

**Conclusions**

These results suggest the need for future studies directly testing the hypothesis of neural correlates of pain levels being encoded in the power of beta and gamma frequency bands in occipital electrodes.

Informed consent was obtained for publication.

#### P67 - Neural correlates of pavement texture during exoskeleton control

##### Júlia Ramos^1^, Miguel Pais-Vieira^2^, Carla Pais-Vieira^3^

###### ^1^ Departamento de Engenharia Electromecânica, Universidade da Beira Interior, Covilhã, Portugal; ^2^ iBiMED - Instituto de Biomedicina, Departamento de Ciências Médicas, Universidade de Aveiro, Aveiro, Portugal; ^3^ CIIS - Centro de Investigação Interdisciplinar em Saúde Porto, Universidade Católica Portuguesa, Porto, Portugal

####### **Correspondence:** Carla Pais-Vieira

*BMC Proceedings 2023,* **17(9):**P67

**Background**

Powered exoskeletons are becoming a widely spread and reliable tool for rehabilitation with recent studies showing that neural plastic effects can be potentiated through their use. Despite these beneficial effects, the neural correlates associated with exoskeleton control have not yet been described in detail. For example, using an exoskeleton in surfaces with different textures is likely to be associated with changes in neural activity, however it is not known if, for example, the cognitive load required for exoskeleton control is so large that sensory processing of pavement textures becomes largely irrelevant. In this pilot study, we set out to describe the effects of different pavement textures in the neural signal of a participant controlling an ExoAtlet ® powered exoskeleton.

**Materials and methods**

In order to measure, process, and analyze the impact of different textures on neurophysiological rhythms, 4-minute signals were recorded with a 16 channel EEG cap (actiCAP by Brain Products) throughout the session. Informed consent was obtained from the participant (Committee for Health Sciences of the Universidade Católica Portuguesa - 99/2022). The subject was instructed to walk in place in 4 different types of pavements (regular, carpet, foam, and rubber circles) with and without the exoskeleton, in a total of 8 different experimental conditions.

**Results**

Analysis of EEG signals revealed that different pavements were associated with changes in the power of specific electrodes and frequency bands.

**Conclusions**

These preliminary results support the notion that exoskeleton control *per se* may not constitute an impediment for sensory processing of pavement textures at the neurophysiological level. Future studies with an increased number of subjects are required to validate the present findings.

Informed consent was obtained for publication.

#### P68 - Ways and means to comfort people at the end of life: An ethnographic study

##### Raquel Alexandra Machado Pereira^1^, Patrícia Pontífice de Sousa^1^

###### ^1^ Universidade Católica Portuguesa, Centro de Investigação Interdisciplinar em Saúde, Lisboa, Portugal

####### **Correspondence:** Raquel Alexandra Machado Pereira

*BMC Proceedings 2023,* **17(9):**P68

**Background**

Comfort is a desirable need throughout life and it is a key element in the practice of nursing care for the patient at the end of life. A particular human need and a state related to the experience and culture of the person at the end of life, constitutes the target of attention and nursing intervention, being a very relevant indicator of the quality of health care. This article reveals the partial results of a doctoral study on comfort as a culture-integrating entity in a palliative care unit. The objectives of this study are to understand the ways and means of comfort perceived by the person at the end-of-life hospitalized in a palliative care unit, their family and health staff as well as the value of the nurse in this process.

**Materials and methods**

We conducted a ethnographic study with a qualitative approach of semi-structured interviews with 18 patients at the end of life; 18 families and 21 health professionals. We also conducted participant observation of care situations.

**Results**

The ways and means of providing comfort are centered on strategies developed by the entire multidisciplinary team. During this whole process, one of the categories that emerged from the ethnography was the nurse as a privileged actor, since he plays an absolutely essential role in all phases. The results revealed that nurses play a very important role in end-of-life comfort, which is based on a predisposition for end-of-life care (active listening, empathy, congruence and biographical narrative) and focused attention (global care, attention to detail, family support and opposition to therapeutic obstinacy).

**Conclusions**

The different ways and means of providing comfort aim to increase care, relieve discomfort and invest in potential different forms of comfort and nurses are recognized by all those involved in this process as someone essential to providing comfort care.

**Keywords**

Comfort; Palliative Care; Etnography; Nursing.

Study approved by the ethics committee and board of directors of Centro Hospitalar Barreiro Montijo, EPE. Reference number: 48/2019.

All participants consented to the publication of the study.

#### P69 - Characterization of the main methodology used by dentists and dental students in the determination of dental color

##### Malainho, L.^1^; Araújo, F.^2^; Rio, R.^2^

###### ^1^ Universidade Católica Portuguesa, Faculdade de Medicina Dentária, Portugal; ^2^ Universidade Católica Portuguesa, Faculty of Dental Medicine, Center for Interdisciplinary Research in Health, Portugal

####### **Correspondence:** Rio, R (rpsousa@ucp.pt)

*BMC Proceedings 2023,* **17(9):**P69

**Background**

Dental Aesthetics is an increasingly relevant subject in today’s society. In order to obtain aesthetic results, it’s extremely important to do a correct shade selection. It is a complex process, realized in the daily dental practice, that can be realized using different methods and it’s influenced by factors such as light, the operator and the object. In order to make a good shade selection it’s important do understand the factors that influence the process to minimize the errors that might occur.

The objective of this study was to analyse which methods are used by dentists and final year dental students in the moment of shade matching, and understand their knowledge on this theme and the degree of difficulty associated with this stage of treatment.

**Material and Methods**

An observational cross-sectional epidemiological study was performed, with a sample of 145 inquired. In order to obtain information a questionnaire about shade selection was applied to dentists and final year dental students who met the following inclusion criteria: degree inn Dentistry or student in the same area; clinical practice in Portugal, volunteer to answer the questionnaire and have access to digital platforms. The questionnaire was carried out on an online platform, the Qualtrics ®. Statistical analysis was performed using the statistical program IBM SPSS® Statistics 23, considering a rate significance of 5%.

**Results**

The majority of the sample (68.3%) reported never having performed any training about color in esthetic restorations.

The visual method with help of VITA® Classic guide (65,5%) was the preferred method of shade selection, 88,3% presented difficulties in the process of shade matching, and 88,3% referred to know the concepts of hue, chroma and value.

**Conclusions**

Shade selection is a subjective process influenced by many factors. Knowledge about color is necessary in order to facilitate the process. Despite the evolutions to the present day the visual method is still the most used.

#### P70 - Influence of a polarizing filter on the determination of dental color

##### Veríssimo, N.^2^; Araújo, F.^1,2^; Rio, R.^1,2^

###### ^1^ Universidade Católica Portuguesa, Centre for Interdisciplinary Research in Health, Viseu, Portugal; ^2^ Universidade Católica Portuguesa, Faculdade de Medicina Dentária, Viseu, Portugal

####### **Correspondence:** Rio, R (rpsousa@ucp.pt)

*BMC Proceedings 2023,* **17(9):**P70

**Background**

Shade matching is an inherent thematic in dental aesthetics, being a complex process and influenced not only by personal characteristics, but also by factors such as light, age, training, protocol and biomaterials used. In order to mimic shade reproduction, it is important to test and understand the new methodologies available.

The objective of this study was to evaluate the agreement in color determination, when a polarizing filter is used and in the absence of it.

**Materials and Methods**

It was an observational cross-sectional study. A total of 306 evaluations were performed in 70 patients using the VITA® Classical and VITA 3D®-Master guide, with and without the use of a polarizing filter (Smile Lite®).

A group of 16 evaluators was constituted to evaluate several colour assessments of the cervical third, middle and incisal in central incisor and upper canine, of the first or second quadrant, according to the established inclusion and exclusion criteria. Each evaluation consisted of 4 parts. Using the VITA® Classic scale ,the VITA 3D® Master scale, a joint use of the Classic VITA scale with the Smile Lite ®and the VITA 3D® Master scale together with Smile Lite®.

**Results**

Regarding the color guides used, the VITA® Classical guide obtained a significantly higher proportion of concordant results. There were significant differences between the thirds regarding the VITA 3D®-Master guide. Regarding the agreement between gender of evaluators, no statistically significant differences were found.

**Conclusion**

According to the results obtained, the influence of Smile Lite ®can be verified in certain situations, namely in the dental division by thirds. Thus, it is a valid and influential element in shade matching. Taking into account the conclusions presented, it is important that further studies are carried out in the future due to the scarce literature on the subject.

#### P71 - The duality between pain and suffering in assisted reproduction techniques: a cross-sectional study

##### Joana Romeiro^1^, Helga Martins^1,2^, Sílvia Caldeira^1^

###### ^1^ Universidade Católica Portuguesa, Institute of Health Sciences, Center for Interdisciplinary Research in Health, Lisbon, Portugal; ^2^ Instituto Politécnico de Beja, Escola Superior de Saúde, Beja, Portugal

####### **Correspondence:** Joana Romeiro (s-jromeiro@ucp.pt)

*BMC Proceedings 2023,* **17(9):**P71

**Background**

Evidence has demonstrated possible side effects and adverse effects due to hormonal medications to increase women's fertility. In addition, frequent and painful therapeutic administrations, successive exposure of the woman's body for examination, and invasive, mechanistic, and dehumanizing procedures have been described by women during fertility treatments.

This study explored perceptions of pain and suffering in women during assisted reproductive techniques.

**Materials and methods**

This was a cross-sectional study conducted from September 2019 to June 2020. The Ethics Committee of The Institute of Health Sciences of Universidade Católica Portuguesa approved this study. The sample was composed of 104 Portuguese adults in the process of engaging or at any stage of a fertility treatment recruited from the web which gave their informed consent to participate in the study. The survey comprised demographic questions, clinical-health aspects, and the first part of the Portuguese version of the Meaning in Suffering Test (MIST-P). Statistical analyses were conducted using SPSS (version 26.0).

**Results**

More than half of the participants in the study identified body changes (n=54; 51.9%) as an aspect that enhances suffering. Moreover, the pain had a high presence among people undergoing fertility treatment (n=82; 78.8%), highlighting the idea of suffering as a profound experience beyond the organic side of living with infertility. Moreover, healthcare practices, such as talking to clients, caring, and providing comfort were strategies suggested by the sample under study.

**Conclusions**

This study highlighted the fact that suffering is not synonymous with pain, although it appears closely related to it. These findings play a fundamental role in future healthcare practices and specifically in the crucial role of nurses in providing a holistic and person-centered approach to improve the quality of life of such individuals and simultaneously raise the quality of nursing care in the reproductive context.

#### P72 - The relationship between religion and the meaning in suffering: perception of people with infertility

##### Joana Romeiro^1^, Helga Martins^1,2^, Sílvia Caldeira^1^

###### ^1^ Universidade Católica Portuguesa, Institute of Health Sciences, Center for Interdisciplinary Research in Health, Lisbon, Portugal; ^2^ Instituto Politécnico de Beja, Escola Superior de Saúde, Beja, Portugal

####### **Correspondence:** Joana Romeiro (s-jromeiro@ucp.pt)

*BMC Proceedings 2023,* **17(9):**P72

**Background**

Religion is a strategy that emerges, is developed, and is often used to face and overcome an adverse health event. Spirituality is essential to maintaining an individual´s mental health. Spiritual practices (such as prayer, mediation, attending religious services, and spending time in nature, reading religious books or self-help texts) are commonly used to recover mental well-being.

**Materials and methods**

For the purpose of the study of exploring the relationship between religion and the meaning of suffering in people with infertility, a cross-sectional study was carried out from September 2019 to June 2020. Informed consent was obtained. The Ethics Committee of The Institute of Health Sciences of Universidade Católica Portuguesa approved this study. The sample was composed of 104 Portuguese adults in the process of engaging or at any stage of a fertility treatment recruited from online forums and social (in)fertility-related websites. The survey comprised demographic questions, information about spiritual and religious beliefs, clinical-health aspects, and the Portuguese version of the Meaning in Suffering Test (MIST-P). Statistical analyses were conducted using SPSS (version 26.0).

**Results**

The religious aspect stood out in the sample (n=20; 66.7%). A comparison between the change in the importance of spirituality/religion after the diagnosis of infertility and after the start of fertility treatment confirmed that there were more changes in the latter context than in the former. The meaning of unavoidable suffering was significantly associated with changes in religion during the infertility diagnosis phase (p = 0.03) with higher MIST-P scores (M = 3.88, SD = 0.81). Religion was identified as a source of spiritual strength and support in the individual's search for meaning in suffering.

**Conclusions**

Further longitudinal research is imperative to understand the profile of meaning in suffering and its relationship with religious and spiritual beliefs through long reproductive treatments.

#### P73 - Studying end-of-life narratives to understand and integrate forgiveness

##### Rita Santos Silva^1^, Joana Bragança^1^, Sílvia Caldeira^1^

###### ^1^ Institute of Health Sciences, Universidade Católica Portuguesa, Lisbon, Portugal

####### **Correspondence:** Rita Santos Silva (s-arcssilva@ucp.pt)

*BMC Proceedings 2023,* **17(9):**P73

**Background**

Since childhood, we live embedded in stories affirmed and reaffirmed throughout our lives as one of the most important ways of defining and shaping cultures and personal interactions. Stories gain relevance in times of transition and/or life change events, such as being a palliative care patient. For those living this condition, life review may bring the need for forgiveness, which seems critical in getting serenity and peace at the end of life. Forgiveness facilitation is a nursing intervention, but a deeper understanding of this phenomenon must inform effective implementation.

This study aims at studying end-of-life narratives to understand and integrate forgiveness.

**Material and methods**

Narrative research is a type of qualitative research in which participants interpret their own experiences, telling their individual stories in their own words to the researcher in an interview. It seeks to minimize the interviewer's influence, avoiding the question-answer structure standard in structured or semi-structured interviews, and avoiding restructuring, as the researcher does not question the interviewee's answers but encourages the interviewee to tell the story.

**Results**

The research findings are, therefore, a joint product of the participant sharing their experiences and the researcher analyzing them.

A narrative showing the connection of someone's experiences to a specific care context and their previous experiences can provide detailed insight into the experiences of a person receiving care.

**Conclusions**

Narrative research is thus a specific innovative approach in the field of qualitative methodology dedicated to the ongoing development of rigorous analytical methods to understand personal and cultural experiences. Health professionals working in palliative care have the opportunity to develop a wide range of narrative research methods to improve health policy, training, and practice in health care.

This is the way to add value to the knowledge of forgiving and being forgiven in palliative care patients, creating new knowledge, and opening up new strategies for the intervention.

### Session 3 - Community Care

#### P74 - Teaching spiritual care in Portuguese nursing schools

##### Ana Afonso^1^, Sara Sitefane^1^, Isabel Rabiais^1^, Sílvia Caldeira^1^

###### ^1^ Institute of Health Sciences, Universidade Católica Portuguesa, Lisbon, Portugal

####### **Correspondence:** Ana Afonso (s-anfiafonso@ucp.pt)

*BMC Proceedings 2023,* **17(9):**P74

**Background**

Spiritual care is an essential dimension of holistic care. Organizations, such as the International Council of Nurses, mention the importance of spirituality for health and the urgency of nurses providing spiritual care. At the same time, studies show that spirituality brings benefits at the level of coping strategies both in crisis or struggling experiences, greater gratitude, facilitating forgiveness and meaning of life. However, spirituality and spiritual care seem to have been neglected and, among other factors, the lack of training in the nursing degree has been described as critical.

The undergraduate nursing degree in Portugal should ensure scientific, technical, human, and cultural training for of providing and managing general nursing care. As so, the undergraduate degree should provide the conditions to learn about spiritual care based on a holistic approach to patients, families, and communities.

The school curriculum and respective syllabuses are not random, and the choice of different curricular units results from reflexive and intentional processes in each institution.

**Materials and methods**

An exploratory study was conducted to map the explicit reference of spiritual care in the undergraduate nursing degree in all nursing schools in Portugal.

Data were collected in September 2022 by searching for the spiritual* research term in the designation of the undergraduate nursing degree curricular units as displayed on the websites of the higher education institutions (HEI).

**Results**

Of the 36 HEI, none had curricular units entitled with spirituality or spiritual care.

**Conclusions**

Although it cannot be inferred that spiritual care is not addressed in the undergraduate nursing degree, as the curriculum is a form of social visibility of a discipline and a profession, these data should lead us to reflect on the (in)visibility that is given to the spiritual dimension. So further studies are needed to disclose and understand how students are prepared to attend to patients in a holistic paradigm that includes attention to the spiritual dimension of health.

#### P75 - Correspondence between language performance of children in formal alternative care and the placement environment: Preliminary data from a systematic review

##### Ana Carolina Capinha^1^, Ana Mineiro^1^, Mara Moita^1,2^, Ana Maria Abreu^1^

###### ^1^ Centro de Investigação Interdisciplinar em Saúde, Instituto de Ciências da Saúde, Universidade Católica Portuguesa, Lisboa, Portugal; ^2^ Centro de Linguística da Universidade NOVA de Lisboa, Faculdade de Ciências Sociais e Humanas, Universidade Nova de Lisboa, Lisboa, Portugal

####### **Correspondence:** Ana Carolina Capinha (anacarolinacapinha@gmail.com)

*BMC Proceedings 2023,* **17(9):**P75

**Background**

An estimated 2.7 million children live in formal alternative care (FAC). FAC varies in living conditions and care provided. However, research has shown that living in FAC adversely affects child development. This should be cautiously interpreted as studies reporting these effects have mainly been conducted in the northern hemisphere, in psychosocially deprived settings. Conversely, due to socio-economic factors, FAC compares favorably to domestic care in low-income countries.

Here, we sought to understand the correspondence between children's language performance in FAC and the placement setting (residential, foster, and kinship care), a query subset from a more extensive main study aiming to investigate children's language development in formal alternative care.

**Materials and methods**

We systematically searched APA PsycInfo, Cochrane Library, Embase, ERIC, MEDLINE, PubMed, Scopus, and Web of Science databases between October and November 2021. The search was not circumscribed to a period. Only primary English reports published in peer-reviewed journals investigating the language performance of children up to age 18 in FAC were included.

**Results**

We identified ten reports that matched these criteria. Eight reports (80%) described changes in the setting in FAC leading to variations in children's linguistic performance. We found that children who transition from low-quality settings (i.e., settings in which some aspect of care is substantially lower than suggested by best practice) to higher-quality environments show a "catch-up effect" in their linguistic performance. When this change happens early, children in FAC have equivalent language performances to the comparison groups (children living with their biological parents). Conversely, children who stay with their families in situations of abuse or exposure to war show lower linguistic performance scores than children in FAC.

**Conclusions**

Thus, not all settings, even if family-based, can be linguistically enriching; there needs to be reciprocity in interactions between carers and children to promote this development. Training and support for carers in all care settings are essential to ensure responsiveness and developmentally appropriate environments for children in FAC.

#### P76 - Development and test of a complex intervention: “Promoting spiritual coping” of family caregivers of an adult relative with severe mental illness

##### Tiago Casaleiro^1,2^ , Helga Martins^1,3^ , Joana Romeiro^1^ , Sílvia Caldeira^1^

###### ^1^ Universidade Católica Portuguesa, Center for Interdisciplinary Research in Health, Lisboa, Portugal; ^2^ Escola Superior de Enfermagem São Francisco das Misericórdias, Lisboa, Portugal; ^3^ Instituto Politécnico de Beja, Escola Superior de Saúde, Beja, Portugal

####### **Correspondence:** Tiago Casaleiro (tcasaleiro@esesfm.pt)

*BMC Proceedings 2023,* **17(9):**P76

**Background**

Severe mental illness is characterized by severe changes in functionality interfering with daily life activities. The home-dwelling person with severe mental illness often needs support from family members who assume the role of caregivers. The performance of the role of family caregiver often leads to a burden, with an impact on physical and mental health. As so, the caregiver develops coping strategies to deal with stressful situations. Among these, spiritual coping strategies are often used, which involve the relationship with oneself, others, and/or the transcendent/God or nature and may include religious or spiritual practices. These strategies are related to improvement in physical and mental well-being. The specialist nurse in mental and psychiatric health has competencies, such as the systemic assessment and intervention based on the biological, psychological, social, cultural, and spiritual dimensions aiming at the promotion of well-being and mental health.

The main goal of this study was to develop and test the intervention “promoting spiritual coping” in the family caregivers of home-dwelling people with mental illness.

**Materials and methods**

A mixed-method study with a sequential exploratory design was conducted, using the development and feasibility/pilot phases of the Medical Research Council’s framework. The development stage included a systematic literature review according to Joanna Briggs Institute guidelines; two focus groups with caregivers and experts; and an online modified e-Delphi. The test phase consisted of a pilot test with ten family caregivers of home-dwelling people with a mental illness accompanied by the community mental health structures of health units in the Lisbon region. The outcomes included spiritual coping, quality of life, and the burden of the family caregiver.

**Results**

A protocol for the intervention was developed and tested with a group of ten family caregivers. A three-session intervention was implemented. The outcomes were assessed pre and post-intervention. Significant changes were observed in the outcomes and the family caregivers mentioned that it was helpful to discuss issues regarding spirituality and religiosity.

**Conclusion**

The intervention "promoting spiritual coping" was developed and tested, considered appropriate for family caregivers of people with mental illness, to be applied in psychotherapeutic context by mental health nurses.

#### P77 - Validation of the European Portuguese version of the Brief RCOPE: a methodological study

##### Tiago Casaleiro^1,2^ , Helga Martins^1,3^ , Joana Romeiro^1^ , Sílvia Caldeira^1^

###### ^1^ Universidade Católica Portuguesa, Center for Interdisciplinary Research in Health, Lisboa, Portugal; ^2^ Escola Superior de Enfermagem São Francisco das Misericórdias, Lisboa, Portugal; ^3^ Instituto Politécnico de Beja, Escola Superior de Saúde, Beja, Portugal

####### **Correspondence:** Tiago Casaleiro (tcasaleiro@esesfm.pt)

*BMC Proceedings 2023,* **17(9):**P77

**Background**

Being a family caregiver often leads to a burden, with an impact on different dimensions of life. As so, the caregiver develops coping strategies to deal with stressful situations. Coping is a multifactorial and individual process related to responding to stressful situations, such as being a caregiver of a relative with health conditions. There are different coping strategies, such as spiritual/religious coping. The 14-item Brief RCOPE is a widely used instrument to assess spiritual/religious coping, but it is not available in European Portuguese. This instrument is a short version of RCOPE which has 63 items. The aim was to translate, adapt and validate the 14-item Brief RCOPE in Portuguese caregivers of an adult relative with a health condition, such as dementia, mental illness, disabilities, among other physical illnesses.

**Materials and methods**

To examine the psychometric properties of the Brief RCOPE it was used the methodological guideline provided by Sousa and Rojjanasrirat.

**Results**

The linguistic and conceptual equivalence of the scale was established. A total of 105 questionnaires were included in this study. The internal consistency was acceptable (Cronbach’s α = 0.86). The Principal Axis Factor (PAF) analysis with varimax rotation identified two factors made up of 13 items, and one item was excluded from the scale.

**Conclusion**

The European Portuguese version of the Brief RCOPE is a reliable and valid measure for assessing the religious coping of family caregivers of adults with health conditions.

#### P78 - Cognitive, emotional, and motivational effects of gamification in the context of learning: A protocol feasibility and usability study

##### Franz Coelho^1^, David Aparício^2^, Patrícia Sousa^1^, Daniel Gonçalves^3^, Ana Maria Abreu^1^

###### ^1^ Center for Interdisciplinary Research in Health (CIIS), Institute of Health Sciences, Universidade Católica Portuguesa, 1649-023 Lisbon, Portugal; ^2^ Escola de Enfermagem, Institute of Health Sciences, Universidade Católica Portuguesa, 1649-023 Lisbon, Portugal; ^3^ INESC-ID and Instituto Superior Técnico – University of Lisbon, Av. Rovisco Pais, 1049-001 Lisbon, Portugal

####### **Correspondence:** Franz Coelho (franzgrc@hotmail.com)

*BMC Proceedings 2023,* **17(9):**P78

Gamification is the use of game elements in non-gaming contexts*.* Empirical studies show that gamification impacts performance, engagement, attention, motivation, and emotions in cognition and learning contexts. However, the literature seems limited by the lack of consistent theoretical support related to gamification; the heterogeneity of results and methodologies; samples without statistical robustness; and the lack of validated questionnaires or non-subjective resources in evaluating individuals. We intend to address these gaps in a future comprehensive randomized control trial (RCT) study. To this end, we carried out a pilot study to assess the feasibility and usability of the RCT. A simple digital course was created and nested (or not) within an e-learning platform, which was adapted to four different versions containing different embedded game elements (“points”, “challenge”, “medals”, and “points + challenge + medals”) and one version without embedded game elements. Ten nursing students were recruited to take the course and were asked to watch the course video lessons, do summative exercises, and answer a final assessment. While doing so, we measured affective states with the Self-Assessment Manikin questionnaire, motivation with the Intrinsic Motivation Inventory, the experience with the User Experience Questionnaire and considered an open question to collect ideas for improvements. We also identified the player profiles with the Brainhex Questionnaire. Correct exercises and the final assessment provided engagement and performance scores. Results showed that the protocol is feasible, but there are adjustments to be made to the e-learning platform and its versions. There were technical issues with running it on different operating systems and configurations. It affected the eye tracker and facial recognition software that were plugged in to collect attention and emotion scores. On the other hand, students were interested in experiencing new forms of learning content. Data from this study will be used to develop new web-based versions of the e-learning platform to circumvent the encountered problems, facilitate the implementation, enable the operation, and afford easier user access. This web-based version will also be used to improve the intervention and evaluate the e-learning interface usability, providing insights concerning the acceptability and adaptability of the interface and protocol to refine the research design of the future RCT study. This study received approval from the Comissão de Ética para a Saúde (# 210) and all the students signed an informed consent before the study.

#### P79 - Characterization of oral health in a group of elderly adults from Viseu – implications for treatment efficacy

##### Sara Sousa^1^, Rafaela Guilherme^1^, Adriana Ribeiro^1^, Nélio Veiga^2^, Maria Correia^2^

###### ^1^ Universidade Católica Portuguesa, Faculty of Dental Medicine (FMD), Viseu, Portugal; ^2^ Universidade Católica Portuguesa, Faculty of Dental Medicine (FMD), Center for Interdisciplinary Research in Health (CIIS), Viseu, Portugal

####### **Correspondence:** Maria Correia (mcorreia@ucp.pt)

*BMC Proceedings 2023,* **17(9):**P79

**Background**

Although it is not recognized and valued by everyone, oral health has a great impact on quality of life, affecting physiological, aesthetic, and social aspects of everyday life. In the elderly oral health issues are prevalent and treatment needs increased. This work presents a characterization of the oral health in an elderly population of Viseu in different dimensions.

**Materials and methods**

Clinical aspects such as tooth loss due to caries, periodontal disease indexes, properties of saliva and total microbial load, as well as *Firmicutes* and *Bacteriodetes* quantification and quality of life indexes were obtained. Questionnaires for sociodemographic data and self-perceived oral health; clinical assessments of oral health indexes and saliva for biochemical and microbiological parameters were used to collect data. Data and sample collection was approved by the ethics committee of IMM and informed consent was signed by every participant in the study.

**Results**

The oral health indicators show that the population analyzed has a DMFT index of 20.82 and 78% of the population presented with periodontal disease (PSR 2-4). Salivary bacterial analysis demonstrated that in saliva Firmicutes (20%) are more prevalent than Bacteroidetes (3%) and the mean F/B ratio was 12,84. No significant differences were found between the total bacterial loads of individuals with different DMFT scores. Results indicate that this populations oral health may be improved especially regarding missing teeth and periodontal *status*. There were no statistical differences in the association between biochemical (pH and total protein concentration) and most microbiological parameters and oral health. However, edentulous individuals presented lower bacterial loads in saliva then dentate individuals.

**Conclusions**

There were differences in the clinically assessed oral health levels and the self-perceived oral health indicating that frequently individuals have low expectations regarding their oral health.

#### P80 - Characterization of the oral health status and literacy among a sample of Portuguese elderly

##### Hélder Costa^1^, Nélio Veiga^1,2^, Patrícia Correia^1,2^, Patrícia Couto^1,2^, Maria José Correia^1,2^, Joaquin López-Marcos^3^

###### ^1^ Faculty of Dental Medicine - Universidade Católica Portuguesa, Viseu, Portugal; ^2^ Center for Interdisciplinary Research in Health – Universidade Católica Portuguesa, Viseu, Portugal; ^3^ Faculty of Medicine – University of Salamanca, Spain

####### **Correspondence:** Hélder Costa

*BMC Proceedings 2023,* **17(9):**P80

**Background**

Health literacy is a main factor in health for its improvement, allowing the individuals to have a greater capacity to engage and participate in collective health promotion actions. In dentistry, oral health literacy (OHL) is the ability to understand information regarding dental services, as well as the prevention, control, and treatment of oral problems. The aim of this study was to characterize the oral health status, behaviors and oral health literacy of a sample of participants in the “Atividade Senior” program developed by the municipality of Viseu, Portugal.

**Materials and methods**

An observational cross-sectional study was designed with a sample of 206 participants of the program that accepted responding to the questionnaire and the application of the Rapid Estimate of Adult Literacy in Dentistry (REALD-29PT), validated for the Portuguese population. An intra-oral observation was accomplished to determine the oral health status of the participant and the decayed, missing and filled teeth index was determined (DMFT index). All the participants signed an explicit and informed consent, and the research was approved by the Health Ethics Committee of the Universidade Católica Portuguesa, with the approval registration number 100.

**Results**

As for gender, 69.4% (n=143) are women and 30.6% (n=63) men, with an average age of 70.0±7.16. Regarding oral health behaviors, 31.7% brush once a day, 81.6% do not use dental floss nor other methods of interdental hygiene and 55.8% had a dental appointment in the last 12 months. The DMFT index was 10.38±8.55, with the higher score corresponding to the missing teeth component (11.96±8.56) and 43.9% of the sample use a dental prothesis. Regarding the application of the REALD-29PT, we can verify that 22.7% have low OHL (score from 0-14), 43.7% moderate OHL (score from 15-22) and 33.6% high OHL (score 23-29).

**Conclusions**

Oral health literacy presents a satisfactory level among more active older adults. However, the reinforcement of oral health promotion strategies is needed to improve oral health status and literacy among the community. These strategies should pass by the development of teaching methods based on oral health behaviors for the community and the implementation of oral health promotion programs directed to adults.

#### P81 - Characterization of soft and hard tissue lesions in an oral medicine unit in the central region of Portugal: a 5-year retrospective study

##### Camila Trimboli^1^, Tiago Marques^2^, Raquel Silva^2^, Nélio Veiga^2^, Patrícia Couto ^2^

###### ^1^ Faculdade de Medicina Dentária, Universidade Católica Portuguesa, Viseu, Portugal; ^2^ Centre for Interdisciplinary Research in Health, Universidade Católica Portuguesa, Viseu, Portugal

####### **Correspondence:** Patrícia Couto (pscouto@ucp.pt)

*BMC Proceedings 2023,* **17(9):**P81

**Background**

The present study aims to assess the prevalence of different types of soft and hard tissue lesions diagnosed through anatomopathological examination, in a sample from the central region of Portugal; to analyze the risk factors associated with these lesions; as well as the prevalence of malignant neoplasms.

**Materials and methods**

A search of the anatomopathological records of soft and hard tissue lesions was carried out, between january 2017 and december 2021. The following variables were analyzed: gender, age at the time of biopsy, exposure to risk factors, anatomical location, and the prevalence of malignant neoplasms. All data were anonymized and kept confidential throughout the investigation, being for the exclusive use of the research team.

**Results**

52 biopsies were performed. The most diagnosed pathology was fibroma, followed by epulis. The gender most affected was the female with 59.62% and the mean age of patients was 56.54 years. Smoking habits and the use of ill-fitting dentures were the most common risk factors among the patients. The prevalence of malignant neoplasms was 5.77%, with a higher incidence among females (3.85%) and in the age group between 71-80 years (3.85%).

**Conclusions**

The various lesions diagnosed confirm that biopsy followed by anatomopathological examination is a highly relevant procedure, together with clinical examination and a detailed anamnesis, to obtain an accurate and definitive diagnosis of oral cavity lesions.

The study protocol was approved by the Ethics Commission for Health of the Universidade Católica Portuguesa (Comissão de Ética para Saúde da UCP, Report number 169, november 18, 2021).

#### P82 - Correlation between the Yesavage Geriatric Depression Scale and the Oral Health Status of the Elderly Patient - Pilot Study

##### Catarina Ramos^1^, Nélio Veiga^2^, Célia Ribeiro^3^, Patrícia Couto^2^

###### ^1^ Faculdade de Medicina Dentária, Universidade Católica Portuguesa, Viseu, Portugal; ^2^ Center for Interdisciplinary Research in Health, Universidade Católica Portuguesa, Viseu, Portugal; ^3^ Research Centre for Human Development, Universidade Católica Portuguesa, Viseu, Portugal

####### **Correspondence:** Patrícia Couto (pscouto@ucp.pt)

*BMC Proceedings 2023,* **17(9):**P82

**Background**

The present study intends to assess the prevalence of signs and symptoms of depression in elderly people in the district of Viseu, and to analyze how these are associated to the oral health status and quality of life.

**Materials and methods**

An observational descriptive cross-sectional pilot study was designed with a sample of 20 participants residing in two institutions in the municipality of Viseu, Portugal. Data collection was carried out through the application of a questionnaire composed of general sociodemographic and oral health aspects, the Geriatric Oral Health Assessment Index (GOHAI), the Yesavage Geriatric Depression Scale (GDS-15) and also through the application of the decayed, missing and filled permanent teeth index (DMFT index).

**Results**

The mean age of the sample was 80±7.74 years, with 85% of the female gender. Of the participating individuals, 55% reported not having dental prostheses. The average GOHAI index was 26.7±5.2. It was also found that 65% of the elderly did not have depression, 30% had "mild depression" and 5% had "severe depression". For those categorized as "depressed", all considered to have an "average" to "poor" condition of their teeth and almost 3/4 had low self-perception of quality of life related to oral health. Even so, no statistically significant values were detected between the severity of depression, age and the DMFT index.

**Conclusions**

It was found that individuals with symptoms of depression have a greater tendency to manifest a low self-perception of quality of life related to oral health, as well as to report more problems with their teeth and gums, although this is not reflected in the results of the DMFT index.

The study protocol was approved by the Ethics Commission for Health of the Universidade Católica Portuguesa (Comissão de Ética para Saúde da UCP, Report number 33, November 21, 2019). Informed consent was obtained from all participants and all methods were performed in accordance with the Declaration of Helsinki principles for medical research involving human subjects and following the requirements established by Portuguese Law n.° 21/2014 for clinical research.

#### P83 - Title: Digital Metric Analysis of mandibles for Forensic sexual diagnosis

##### Carolina Silva^1^, Cristina Figueiredo^1,2^

###### ^1^ Faculty of Dental Medicine (FMD), Universidade Católica Portuguesa, Viseu, Portugal; ^2^ Center for Interdisciplinary Research in Health (CIIS), Universidade Católica Portuguesa, Viseu, Portugal

####### **Correspondence:** Cristina Figueiredo (cristinafigueiredo@ucp.pt)

*BMC Proceedings 2023,* **17(9):**P83

**Background**

Sexual Diagnosis is a fundamental step towards the Biological Profiling of individuals in Forensic cases. Mandible is one of the most dimorphic bones in the skull, being relevant to sex determination. With technological advancements observed in our era, it was realized the usefulness and precision of Cone Beam Computed Tomography (CBCT) in collection and analysis of bone structures. The main objective of this study is to analyze sexual dimorphism in a collection of Portuguese mandibles belonging to the Portuguese National Institute of Legal Medicine and Forensic Sciences, through a digital metric methodology.

**Materials and Methods**

An observational, cross-sectional study was performed. This study was carried out according to ethics clearance number CE-28/2020. 33 mandibles (14 female, 19 male) and personal belongings were photographically registered. All mandibles were scanned using CBCT and measurements were done through the Simplant Pro 17.01 software. Descriptive and inferential analysis were used to identify the more dimorphic parameters in the sample, and best sex predictors.

**Results**

There was a statistically significant difference in the following parameters: coronoid process height, condyle height, maximum length of mandible and minimum width of mandibular ramus (p < 0,05). In the multivariate statistical analysis, by the stepwise methodology, it was possible to identify the coronoid process height as the best sex predictor, accurately in 72.2% of cases. Allowing to differentiate female and masculine jaws with an accuracy of 64.3% and 78.9%, respectively.

**Conclusions**

Images captured by CBCT provide more information and enable the examination of structures that are difficult to access, when compared to 2D observation and analysis. This methodology is increasingly used as it helps in diagnosing and planning treatments in the dental office. In a legal context, this methodology presents itself as an added value in the collection of information for the identification of individuals. The coronoid process height is the most dimorphic parameter and the best sex predictor in the sample. This results are in line with the existing literature.

#### P84 - The learning of Dignity by Nursing Undergraduate Students: a Grounded Theory

##### Hugo Franco^1^, Silvia Caldeira^1^, Lucília Nunes^2^

###### ^1^ Center for Interdisciplinary Research in Health, Universidade Católica Portuguesa, Lisboa, Portugal; ^2^ NURSE’IN: Nursing Research Unit for South and Islands, Instituto Politécnico de Setúbal, Setúbal, Portugal

####### **Correspondence:** Hugo Franco

*BMC Proceedings 2023,* **17(9):**P84

**Background**

Dignity is a fundamental principle in the practice of nurses. However, the literature reveals gaps in the evidence on how students learn to conceptualize the dignity of the people they care for. The purpose of this research was to design a grounded theory on the social learning process of dignity based upon the experiences and perceptions of undergraduate nursing students.

**Materials and methods**

This qualitative study was conducted using the grounded theory method. Data was collected through free reports, individual semi-structured interviews with 20 undergraduate nursing students, and a focus group. Participants were selected across purposeful sampling and analyzed simultaneously using the Corbin and Strauss approach. The investigation was approved by the Research Ethics Committee of the Institute Polytechnic of Setúbal (Ethics code: n°64/A/CC/2021). Also, after explaining the study’s objectives, all the participants completed and signed a written consent form.

**Results**

The “Reckoning of dignity” was the study’s core category, reflecting the nature of the learning process of dignity. The theory of “Reckoning of Dignity” is based on five main categories: “proto awareness of dignity”, “nursing path”, “awareness of dignity “, “ways of learning” and “becoming able”. These categories cover the underlying social factors, learning strategies, and outcomes of the reckoning process of dignity by undergraduate nursing students. The study findings showed that the undergraduate nursing students experience an acknowledgement process of the practical identity of dignity, which is strengthened throughout the social learning process during the undergraduate course.

**Conclusions**

The *theory of reckoning of dignity* can be used as a practical guide to describe and understand the process of how moral and ethical arch occur and can be boosted during the learning process of undergraduate nursing students.

#### P85 - The impact of MAIEC application in the community empowerment increase, regarding organizational climate, in a business community – a case study

##### Pedro Melo^1^, Carlos Pinto^1^, Cláudia Telles de Freitas^1^, Márcia Fontes^1^, Camila Landim Almeida^1^, Patrícia Gonçalves^2^

###### ^1^ Center for Interdisciplinary Research in Health, Universidade Católica Portuguesa, Porto, Portugal; ^2^ Institute of Health Sciences, Universidade Católica Portuguesa, Porto, Portugal

####### **Correspondence:** Pedro Melo (pmelo@ucp.pt)

*BMC Proceedings 2023,* **17(9):**P85

**Background**

According to the Litwin and Stringer model, the organizational climate of a company is a key factor in the development of productivity and well-being of employees. In Community Health Nursing, there is a framework for approaching communities as a care unit - the Community Intervention and Empowerment Assessment Model (MAIEC). The aim of this study was to assess the impact in the level of community empowerment after applying the MAIEC's clinical decision-making matrix, in an educational business community in Porto.

**Materials and methods**

The level of community empowerment for promoting a healthy organizational climate with members of the business community was assessed, applying the Portuguese version of the Community Empowerment Rating Scale. For the evaluation of community management, a questionnaire was applied to community members, according to the MAIEC clinical decision matrix. Interventions from the same matrix were then applied for two years and the level of empowerment was reassessed after that period, using the same methodology from the diagnostic phase. The project was approved by the ethics committee of ARS Norte under N°51/2012-CE_ARSN.

**Results**

The level of community empowerment increased significantly in the different domains of the scale and community management for the promotion of a healthy organizational climate improved. Also, the community management for respond to the same issue was improved.

**Conclusions**

MAIEC is a model that enhances community empowerment to promote a healthy organizational climate in companies, while improves the community management to respond those issues with autonomy. It is suggested the application of the model in other types of company and with other problems.

#### P86 - Approaching Hospital as a community- A case study regarding professional stress and burnout and the application of MAIEC

##### Pedro Melo^1^, Carlos Pinto^1^, Cláudia Telles de Freitas^1^, José Teixeira^1^, Ana Isabel Campos^3^, Patrícia Coelho^2^

###### ^1^ Center for Interdisciplinary Research in Health, Universidade Católica Portuguesa, Porto, Portugal; ^2^ Institute of Health Sciences, Universidade Católica Portuguesa, Porto, Portugal; ^3^ Hospital da Horta, EPER, Ilha do Faial, Açores, Portugal

####### **Correspondence:** Pedro Melo (pmelo@ucp.pt)

*BMC Proceedings 2023,* **17(9):**P86

**Background**

According with Melo theory, the Hospital can be approached as a client community of Nurses. The use of the Community Assessment, Intervention and Empowerment Model (MAIEC) allows the diagnosis of community management and the enhancement of community empowerment as a process and as result of community health nursing care.

**Materials and methods**

The level of community empowerment for addressing professional stress and burnout was evaluated with representatives of all professional classes and hospital community services, applying the Portuguese version of the Community Empowerment Rating Scale. For the evaluation of community management, a questionnaire was applied to the same community members, according to the MAIEC clinical decision matrix. The project was approved by the ethics committee of Hospital da Horta, EPE N° 1/2017.

**Results**

The level of community empowerment was very low in the different domains of the scale (with a slight increase in "links to others") and community management for preventing and addressing work-related stress and burnout was compromised (with a committed community process, associated with ineffective community coping; a committed community participation related to the lack of organizational structures and partnerships related to this problem and a committed community leadership, especially in the knowledge not demonstrated. Interventions were developed, such as training of community members and the creation of a commission which was constituted as the heart of leadership for the continuity of the process.

**Conclusions**

Hospital communities can be the target of care in Community Health Nursing and MAIEC is a reference that enhances the diagnosis and interventions that promote community empowerment in these communities.

#### P87 - Climate Change approach trough community empowerment- a case study

##### Pedro Melo^1^, Maria João Costa^1^, Vanessa Monteiro^1^, Alexandra Leitão^2^, Rosa Silva^1,3^

###### ^1^ Center for Interdisciplinary Research in Health, Universidade Católica Portuguesa, Porto, Portugal; ^2^ Research Centre in Management and Economics, Universidade Católica Portuguesa, Portugal; ^3^ Escola Superior de Enfermagem do Porto, Porto, Portugal

####### **Correspondence:** Pedro Melo (pmelo@ucp.pt)

*BMC Proceedings 2023,* **17(9):**P87

**Background**

Climate change is one of the major problems that the world is facing and it has repercussions on several dimensions of sustainability, such as health and the economy. The Community Intervention and Empowerment Assessment Model (MAIEC) is a framework that allows enhancing community empowerment through diagnosis and interventions in Community Health Nursing. The aim of this study was to assess the level of community empowerment and diagnose community management, using the MAIEC clinical decision matrix, with the stakeholders of the Atlantic Front of Porto community.

**Methodology**

Stakeholders were identified through a scoping review. To assess the level of community empowerment, the Portuguese version of the Community Empowerment Rating Scale was used, in a focus group with members of municipalities, academia and science, non-governmental associations and public health and civil protection services. For the evaluation of community management, a questionnaire was applied to the same stakeholders, according to the MAIEC clinical decision matrix. This article is a result of the project HAC4CG- Heritage, Art, Creation for Climate change. Living the city: catalyzing spaces for learning, creation and action towards climate change (NORTE-01-0145-FEDER-000067), supported by Norte Portugal Regional Operational Programme (NORTE 2020), under the PORTUGAL 2020 Partnership Agreement, through the European Regional Development Fund (ERDF), and submitted for the approval of the ethics committee of Technology, Social Sciences and Humanities of the Catholic University of Portugal.

**Results**

A low level of community empowerment for adaptation and mitigation in the context of climate change was identified. Community management was compromised in several dimensions (such as community process - lack of experiences and ineffective community coping; community participation - lack of organizational structures and partnerships and community leadership - knowledge not demonstrated and beliefs compromised). Interventions that respond to the diagnoses are being planned.

**Conclusions**

MAIEC is a benchmark that enhances community-wide solutions to respond to the challenges of climate change. Community Empowerment is an important process and also can be a result of community and public health intervention to promote adaptation and mitigation concerning climate change.

#### P88 - Psychometric properties of an instrument to assess students’ performance in problem-based learning tutorials

##### Sofia Menéres^1,2^, Bruno António Cardoso^1,2^, João Pereira^1^, Andreia Gaspar^2^, Pedro Mateus^1,2^, Frederico Simões do Couto^1,2^

###### ^1^ Centro de Investigação Interdisciplinar em Saúde, Universidade Católica Portuguesa, Lisbon, Portugal; ^2^ Departamento de Educação Médica, Faculdade de Medicina, Universidade Católica Portuguesa, Sintra, Portugal

####### **Correspondence:** Sofia Menéres (smeneres@ucp.pt)

*BMC Proceedings 2023,* **17(9):**P88

**Background**

In problem-based learning (PBL) curriculum, tutorial groups are the cornerstone of students’ learning processes, as they trigger constructive, contextual, collaborative, and self-directed learning processes that are known to develop long-term and significant learnings [1]. Moreover, the tutorial group functioning has an impact in time spent on self-study [2]. For these reasons, assessing the way students behave in PBL tutorial groups plays a critical role in promoting students positive and fruitful ways of participation. Sim et al. (2006) [3]. developed a four dimension – i) participation and communication, ii) cooperation and team building, iii) comprehension and reasoning skills, and iv) knowledge and information gathering skills – instrument to assess students’ performance in PBL tutorials. They reported good reliability regarding intra-class correlation coefficient (ICC), as well as the ability to discriminate “strict” and “indiscriminate” tutors. The present study aims to extend the previous findings on the psychometric properties of this instrument by further analyzing the ICC, coupled with a measure of external validity. The latter will be assessed concerning the correlations between students’ performance in PBL tutorial groups and their final exam marks in the different curricular units.

**Materials and methods**

The sample used was a Portuguese group of medical students, encompassing data from 8 blocks (seven from year I and one from year II), covering the academic years of 2021-22 and 2022-23, in a total of 138 students and 46 tutors.

**Results**

Preliminary results showed for block 1.1 a weak positive correlation between students’ overall performance on PBL assessment and final exam of curricular unit (overall R^2^= 0.26, p<0.05). Correlations between dimensions one (i.e., participation and communication) and two (cooperation and team building) and the final exam were also positive and weak (overall R^2^= 0.33, p<0.001 and overall R^2^= 0.30, p<0.001). Dimensions three (i.e., comprehension and reasoning) and four (i.e., knowledge and information gathering skills) showed moderate positive correlations with the final exam (overall R^2^= 0.42, p<0.001 and overall R^2^= 0.46, p<0.001).

**Conclusions**

Results will be discussed considering the adequacy and limitations of this instrument in assessing medical students’ behavior during their PBL discussions.

**References**

1. Dolmans, D.H.J.M. How theory and design-based research can mature PBL practice and research. Adv in Health Sci Educ. 2019; 24:879–891.

2. Dolmans DH, Wolfhagen HA, Scherpbier AJ. From quality assurance to total quality management: how can quality assurance result in continuous improvement in health professions education? Educ Health (Abingdon). 2003;16(2):210-217.

3. Sim SM, Azila NM, Lian LH, Tan CP, Tan NH. A simple instrument for the assessment of student performance in problem-based learning tutorials. Ann Acad Med Singap. 2006; 35:634-641.

#### P89 - The ethnographic method in primary research studies published by nurses: scoping review

##### Raquel Pereira^1^, Teresa Silveira^2^, Patrícia Pontífice de Sousa^3^

###### ^1^ Centro Hospitalar Barreiro Montijo, Barreiro, Portugal; ^2^ Escola Superior de Saúde da Cruz Vermelha Portuguesa-Lisboa, Lisboa, Portugal; ^3^ Universidade Católica Portuguesa - Instituto de Ciências da Saúde de Lisboa, Lisboa, Portugal

####### **Correspondence:** Raquel Pereira

*BMC Proceedings 2023,* **17(9):**P89

**Background**

The perception that each culture has regarding the various social determinants is of great importance, as it is through subjectivity that people tend to build and give meaning to their experiences. We defined the objective of analyzing the existing scientific evidence about the use of the ethnographic method in primary research studies carried out by nurses.

**Materials and methods**

A review of the type of scope was carried out according to the method defined by the Joanna Briggs Institute for Evidence Based Practice. The research question that guided this research was structured in the participants, concept and context (PCC) format: What are the primary research studies (Participants) published by nurses (Context) that use the ethnographic method (Concept)? The research was developed from the EBSCOHost platform, in the databases Cinahl, Medline, Nursing & Allied Health Collection, Comprehensive and Mediclatina. We also used the Scielo database. The descriptors used in each of the databases were validated according to Mesh.

**Results**

109 articles related to primary studies were included. Ethnographic studies carried out by nurses are very heterogeneous and focus not only on the nurses themselves, but also on health professionals and on the entire multidisciplinary team. The articles also point out the need for professionals to know and strengthen themselves with care experiences and health and disease convictions, behaviors and values ​​of families and groups, in order to provide effective and quality nursing care.

**Conclusions**

The results indicate that the ethnographic method applied to research in nursing is transversal to the clinical practice of nurses, as well as being in line with all areas of this professional's functional performance (clinical, teaching and management).

**Keywords**

Ethnography; Qualitative Research; Nurses

#### P90 - Cognitive flexibility in the preschool population

##### Daniela Sciaccaluga^1^, Filipa Ribeiro^1,2^

###### ^1^ Universidade Católica Portuguesa, Instituto de Ciências da Saúde, Lisboa, Portugal; ^2^ Center for Interdisciplinary Research in Health, Lisboa, Portugal

####### **Correspondence:** Filipa Ribeiro (filipa.nc.ribeiro@ucp.pt)

*BMC Proceedings 2023,* **17(9):**P90

There is a need to characterize the typical developmental trajectory of cognitive flexibility in the Portuguese preschool population and, at a global level, to explore its evolution by semesters. In parallel, few studies report data on the impact of gender on cognitive flexibility in this population. This study seeks to answer these questions. We homogeneously distributed 90 typically developing preschoolers in terms of the number of elements and gender in 3 age groups (Group 3-years-old, *M* = 41.16; *SD* = 3.18; Group 4-years-old, *M* = 53.76; *SD* = 3.19; in months; Group 5-years-old, *M* = 65.60; *SD* = 3.76; in months) and submitted to the Card Sorting task of the Early Years Toolbox, based on the prototypical procedure for assessing cognitive flexibility in this population. The institutional review board has approved this study. We obtained a signed informed consent form from each kindergarten and each parent to include their child in this study. We observed significant differences in the switch accuracy variable between all age groups, with older ones overcoming the youngest. When we split the sample by semesters, there were no significant differences between children older than four and a half years old and the younger four-year-olds. All five years old children showed a significantly higher performance than the older four-year-olds. We observed no significant effect of sex on Card Sorting performance. The present study supports the literature by showing an increase in cognitive flexibility over the preschool period, suggesting that the inflection point of this executive function occurs in the transition between the second semester of the four years of age and the first semester of the five. This study represents the first effort to characterize the development of cognitive flexibility in the Portuguese preschool population through a prototypical procedure for assessing this function in this age group. Considering the applicability of Card Sorting to the clinical and investigative context, further research is necessary to investigate the consistency of these findings in the Portuguese preschool population.

**Keywords**

Cognitive flexibility, Preschoolers, Card Sorting, Set shifting.

#### P91 - Spiritual aspects of living with spinal cord injury while in rehabilitation: a qualitative review

##### Liliana Roldão^1^, Joana Romeiro^1,2^, Tiago Casaleiro^1,2^, Helga Martins^1,2,3^, Sílvia Caldeira^1,2^

###### ^1^ Instituto de Ciências da Saúde, Universidade Católica Portuguesa, Lisbon, Portugal; ^2^ Instituto de Ciências da Saúde, Universidade Católica Portuguesa, Center for Interdisciplinary Research in Health, Lisbon, Portugal; ^3^ Escola Superior de Saúde, Instituto Politécnico de Beja, Beja, Portugal

####### **Correspondence:** Liliana Roldão (s-lroldao@ucp.pt)

*BMC Proceedings 2023,* **17(9):**P91

**Background**

According to the World Health Organization, between 250,000 and 500,000 people worldwide suffer a spinal cord injury (SCI) each year, and more than 90% cases are traumatic. These injuries cause profound changes in life and have a negative implication on well-being and quality of life. Spirituality may represent a critical dimension in living and overcoming this condition which usually happens unexpectedly. As so, the healthcare approach considers a holistic paradigm in which individuals are unique and require dignity preserving care. This review aims to identify the spiritual aspects of the experience of adults living with SCI while in the rehabilitation process.

**Materials and methods**

This review included qualitative studies focusing on spiritual needs, spiritual responses, or spiritual practices of adults with SCI spinal cord injury while in rehabilitation process. No date limits were be applied. No language restrictions were be applied.

The databases searched included CINAHL complete MEDLINE ,PubMed, Nursing and Allied Health Collection, CDSR (Cochrane Database of Systematic Reviews), PsycINFO, MedicLatina, and SciELO – Scientific Electronic Library Online. The search for unpublished studies will include Open Grey, RCAAP (Portuguese open access scientific repository), CAPES Brazil – Theses and dissertations. Study selection, critical appraisal, data extraction, and data synthesis were performed by two reviewers, as the review team was organized in each stage to guarantee independent and blind review. The synthesized findings were graded according to the ConQual approach for establishing confidence in findings. Software SUMARI was used to all reviewing process.

**Results**

Two synthesized findings emerged: spiritual coping strategies used by patients living in this condition (related to beliefs and values); spiritual needs are expressed by these patients in different phases while in the rehabilitations, such as faith, hope, meaning and purpose.

**Conclusions**

Spiritual needs have been identified and spirituality seem a coping mechanism used by these patients. The evidence from this qualitative review may inform new nursing intervention that can be implemented and tested.

**Keywords**

Spirituality; Experience; Rehabilitation; Qualitative research; Injuries

#### P92 - Validation of an instrument to assess the meaning in suffering: a methodological study

##### Joana Romeiro^1^, Helga Martins ^1,2^, Tiago Casaleiro^1,3^, Sílvia Caldeira^1^

###### ^1^ Universidade Católica Portuguesa, Institute of Health Sciences, Center for Interdisciplinary Research in Health, Lisbon, Portugal; ^2^ Instituto Politécnico de Beja, Escola Superior de Saúde, Beja, Portugal; ^3^ Escola Superior de Enfermagem São Francisco das Misericórdias

####### **Correspondence:** Joana Romeiro (s-jromeiro@ucp.pt)

*BMC Proceedings 2023,* **17(9):**P92

**Background**

Suffering is a complex, multidimensional and individual experience extensively described in literature concerning different life and health conditions, but often reduced to a physical perspective as a symptom. The relationship between suffering and meaning in life led to the development of measurement tools such as it is the case of the Meaning in Suffering Test (MIST). Additionally, recent studies have awakened the interest in the spiritual dimension and meaning in life lived by people with a reproductive health-condition, such as infertility. As such, there is an urge to study the psychometric properties of the Portuguese version of the Meaning in Suffering Test in the Portuguese context. Up to now, only one Portuguese study aimed to validate such tool in a sample of Portuguese adult patients with chronic rheumatic pain.

**Materials and methods**

More studies are needed to support structural validation and testing of the psychometric properties of MIST in different sets and samples. The Portuguese version of the Spiritual Wellbeing Questionnaire is a tool that could be used in such validation process. This study was approved by the Ethics Committee of Universidade Católica Portuguesa. It was performed a confirmatory factor analysis with a sample of 104 persons under fertility treatment.

**Results**

It was revealed a poor fit of previous structures and as such an exploratory factor analysis provided a final version with 16 items with three factors: “Meaning in life and subjective characteristics in the face of suffering”; “Positive responses to suffering”; and “Loss of control over suffering”.

**Conclusions**

Further research is necessary to better understand fluctuations in individuals undergoing long-term treatments and test the 16-item and three-factor version as a suitable, valid, and reliable structure for measuring meaning in suffering by nurses providing care to different patients with different health conditions and context.

#### P93 - Oral health related quality of life in institutionalized elderly in Viseu

##### Sandra Balula^1^, Ana Margarida Silva^1,2,^, Patrícia Couto^1,2^, Cristina Figueiredo^1,2^, André Correia^1,2^, Nélio Veiga^1,2^, Javier Montero^3^

###### ^1^ Universidade Católica Portuguesa, Faculdade de Medicina Dentária, Viseu, Portugal; ^2^ Universidade Católica Portuguesa, Center for Interdisciplinary Research in Health, Viseu, Portugal; ^3^ University of Salamanca, Faculty of Medicine, Department of Surgery, Spain

####### **Correspondence:** Ana Margarida Silva (amsesilva@ucp.pt)

*BMC Proceedings 2023,* **17(9):**P93

**Background**

Portugal has an increasingly aging population, due to the raise of average life expectancy and low birth rates. This situation constitutes a challenge for public health, particularly regarding oral health related quality of life. The objective of this study is to assess the impact of oral health on the quality of life of institutionalized elderly people in the district of Viseu, Portugal.

**Materials and methods**

An observational descriptive cross-sectional study was designed, composed of a population of elderly residents in nursing homes in the district of Viseu. Data collection was accomplished by an application of a questionnaire to assess sociodemographic and oral health aspects, the GOHAI index and the OHIP-14 index.

**Results**

The sample consisted of 529 institutionalized elderly aged 65 years or older. As for gender, 69.4% (n=367) are women and 30.6% (n=162) men. According to the GOHAI index, a considerable number of participants (41.5%) have a “moderate” self-perception of oral health. Regarding the OHIP-14 index, it appears that the impact of oral health on quality of life is low, since the average of the global OHIP-14 was 15 (0-56). “Physical pain” was the most affected dimension (2.70), while the dimension with the least impact was “Social limitation” (1.52).

**Conclusion**

in this research, oral health related quality of life was considered reasonable. However, there are participants in whom the impact of oral health in the quality of life is significant. Therefore, it is important to continue to develop strategies and join efforts to improve oral health and, consequently, the systemic health and quality of life of institutionalized elderly.

The study protocol was approved by the Ethics Commission for Health of the University (Comisssão de Ética para a Saúde da UCP, Report number 165, 21^st^ of January 2022).

Informed consent was obtained from all participants and all methods were performed in accordance with the Declaration of Helsinki principles for medical research involving human subjects and following the requirements established by Portuguese Law nr 21/2014 for clinical research.

#### P94 - "Ser Criança" Project - oral health literacy strategies for the vulnerable children and families

##### Nelio Veiga^1,2^, Mario Oliveira^1^, Beatriz Dias^1^, Ana Sofia Duarte^1,2^ , Maria Correia^1,2^, Anna Carolina Volpi Mello-Moura^1,2^

###### ^1^ Faculty of Dental Medicine - Universidade Católica Portuguesa, Viseu, Portugal; ^2^ Center for Interdisciplinary Research in Health – Universidade Católica Portuguesa, Viseu, Portugal

####### **Correspondence:** Nelio Veiga

*BMC Proceedings 2023,* **17(9):**P94

**Background**

Over the years, there has been an increasing and effective integration and participation of oral health in the concept of general health. The absence of educational interventions for more vulnerable children, as well as the application of behavioral strategies are still considered gaps in today's society and healthcare services. The main objective of this research was to define strategies of educational interventions in vulnerable children's and their families oral health.

**Materials and methods**

The interventive actions for the “Ser Criança” Project involves the development of a website - “Ser Criança – Aprender e Sorrir” for three target groups: children, parents and educators / teachers. With this goal, it is expected that children, families and school communities change behavioral habits related to oral health. Also the “Ser Criança” Project involves a specific protocol defining oral health literacy strategies and dental appointments among the children and families.

**Results**

It is crucial to emphasize that most recreational activities are beneficial in the transmission of motivational values. The use of educational games, exploration of macro dental models, theater and music are valid examples of these activities. Interventions based on digital media (applications or *“apps”* and the Internet) also prove to be a constant demonstration of success for children's personal and cognitive growth. The development of a digital tool aimed at pre-school and school education, through a web-based aspect allows, especially in the context of the difficult access to oral health services, an immeasurable access to multiple possibilities. A specific oral health appointment protocol was also developed and will be applied among children and their families. This protocol will permit to diagnose oral diseases by teledentistry method, formulating an on-line appointment. Both strategies will be applied among the most vulnerable communities in order to improve the oral health behaviors and diagnosis of oral diseases, mainly, among children.

**Conclusions**

Educating the next generation using attractive educational, didactic and, above all pedagogical interventions can revolutionize the current teaching landscape, especially in the field of oral health. These strategies shall be applied in the future to study the impact in the oral health and health literacy of vulnerable communities.

**Keywords**

Intervention, children, oral health literacy, digital education.

#### P95 - Understanding the barriers and opportunities for implementing serious games-based rehabilitation as a policy in Portuguese healthcare

##### Catarina Vieira^1,2^, André Perrotta^3^, João Novais^4^

###### ^1^ Universidade Católica Portuguesa, Research Center for Science and Technology of the Arts, 4169-005 Porto, Portugal; ^2^ Universidade Católica Portuguesa, Escola das Artes, 4169-005 Porto, Portugal; ^3^ André Perrotta, Research Center for Informatics and Systems, Informatics Engineering Department, Universidade de Coimbra, 3030-290 Coimbra, Portugal; ^4^ Católica Porto Business School, Universidade Católica Portuguesa, 4169-005 Porto, Portugal

####### **Correspondence:** Catarina Vieira (catarina.vieira.28@hotmail.com)

*BMC Proceedings 2023,* **17(9):**P95

**Background**

Serious games (SG) are known to have a wide range of applications. Physical rehabilitation is one of them. As the aging index of the Portuguese population steadily increases, it is necessary to improve physical rehabilitation (as a line of therapy for pathologies and incapacities related to old age). This paper aims to find a relationship between the barriers to SG implementation and the way the current and future contexts will bridge the gap between aspirations, necessity and financial viability.

**Materials and methods**

A PEST (political, economical, social and technological) market analysis of the Portuguese context was conducted, along with an *in loco* investigation achieved through a series of three interviews (to a neurology clinic, to a physiatry clinic and rehabilitation center located in the north of Portugal, and to a Professor and researcher from the Medical School of Universidade do Minho), and a 24-question questionnaire targeted at healthcare professionals (ranging from doctors to nurses, and from heterogeneous specialization areas). 59 subjects participated in the questionnaire. The study in question was submitted and approved by CES-UCP. All participants gave their informed consent before participation in the study.

**Results**

Portugal has an aged population that needs suitable healthcare, which englobes adequate physical rehabilitation when needed. However, there is a lack of human and material resources. When looking at SG as a potential tool, the industry has a good growth rate, and SG present themselves as a way of channeling human and monetary resources. The lack of knowledge about SG, lack of appropriate SG to use for therapeutic purposes as well as access to them, their cost, the age and social status of the patients, or a prevalent preference towards traditional methods are seen as the main barriers to a wider implementation of SG for physical rehabilitation in the Portuguese territory.

**Conclusions**

SG proved to be widely unknown among the healthcare sector in Portugal. Additionally, as older generations show little to no interest in the potential of videogames as a form of entertainment, it will make them resistant towards learning about the clinical benefits of using SG for physical rehabilitation. Socially and financially, the Portuguese context supports the implementation of SG as a complementary tool, allowing physical rehabilitation to reach more people at a lower cost while stimulating therapy adherence and supporting a sustainable allocation of funds.

#### P96 - Experience reported by the person submitted to a hip or knee arthroplasty, related to the continuity of nursing care

##### Patrícia Câmara^1,2^, Élvio H. Jesus^1^, Beatriz Araújo^1^

###### ^1^ Universidade Católica Portuguesa, Center for Interdisciplinary Research in Health, Institute of Health Sciences, Porto, Portugal; ^2^ Higher School of Nursing São José de Cluny, Funchal, Portugal

####### **Correspondence:** Patrícia Câmara (s-pmcamara@ucp.pt)

*BMC Proceedings 2023,* **17(9):**P96

**Background**

Considering the experience of the person submitted to a hip or knee arthroplasty, related to the continuity of nursing care, namely the preparation of their return home, is a key result of quality in health. Therefore, the evaluation of the experience “is seen as a support to improve the quality of health care, management and public responsibility” [1]. Thus, the goal of this study is to identify the dimensions related to the person’s experience who has been submitted to a hip or knee arthroplasty, towards nursing care, in the preparation of the return home.

**Materials and methods**

This is a qualitative, exploratory-descriptive study. Non-probabilistic convenience sampling. The participants were people submitted to a hip or knee arthroplasty, who found themselves at their home and freely agreed to participate in the study. The data was collected through a semi-structured interviews guide and analyzed through a deductive method [2], using the content analysis technique [3] and the cycle coding technique [4], supported by NVIVO1.7® software. Approved by the Ethics Committee - Document of the SESARAM Ethics Committee, EPE n. °: 29/2019.

**Results**

Eight dimensions were identified: prevention of complications and control of symptoms; prescribed therapeutic regimes; adaptability to changes; communication; accessibility; quality of life; participation and shared decision-making; feelings and emotions. These results show the real experience’s dimensions, reported these people themselves, allowing to contribute to good nursing practices development, in order to guarantee a high level of self-experience, safety and health’s quality of life.

**Conclusion**

Studying the person's experience, reported by themselves, means to place the person at the center of health care. As such, it proves to be pressing to investigate, in order to obtain metrics that allow not only to improve the person's own experience, but also to offer new answers, in a *continuum* of improvement, of nursing care in the preparation of returning home. Nurses, embedded in the healthcare team, are valued by people, in their continuum of care.

The authors declare under their responsibility that this article is original, has never been sent to another publication and in its elaboration all scientific, methodological and ethical rigor was taken into account (Document of the SESARAM Ethics Committee, EPE n. °: 29/2019). Furthermore, they declare that they don’t have other conflicts of interest. Informed consent was obtained for publication.

**Keywords**

Continuity of Patient Care; Nursing Care; Arthroplasty; Quality of Life; Safety; Patient experience; Returning home preparation.

**References**

1. Organisation for Economic Co-operation Development Issuing Body. Caring for quality in health: lessons learnt from 15 reviews of health care quality. Paris: OECD. 2017. https://www.oecd.org/els/health-systems/Caring-for-Quality-in-Health-Final-report.pdf.

2. Mayring P. Qualitative content analysis: theoretical foundation, basic procedures and software solution. 2014. https://nbn-resolving.org/urn:nbn:de:0168-ssoar-395173.

3. Bardin, L. Análise de Conteúdo. São Paulo: Edições 70, 2016.

4. Saldaña, J. The coding manual for qualitative researchers. 2ª edition. Londres: Sage, 2013.

